# Current knowledge and perspectives of *Paenibacillus*: a review

**DOI:** 10.1186/s12934-016-0603-7

**Published:** 2016-12-01

**Authors:** Elliot Nicholas Grady, Jacqueline MacDonald, Linda Liu, Alex Richman, Ze-Chun Yuan

**Affiliations:** 1 London Research and Development Centre, Agriculture & Agri-Food Canada, 1391 Sandford Street, London, ON N5V 4T3 Canada; 2 Department of Microbiology & Immunology, Schulich School of Medicine & Dentistry, University of Western Ontario, Dental Science Building Rm. 3014, London, ON N6A 5C1 Canada

**Keywords:** Antimicrobials, Biocontrol, Biofertilizer, Biological nitrogen fixation, Biopesticide, *Paenibacillus*, PGPR, Plant growth promotion, Biomass degradation, Bioproducts

## Abstract

Isolated from a wide range of sources, the genus *Paenibacillus* comprises bacterial species relevant to humans, animals, plants, and the environment. Many *Paenibacillus* species can promote crop growth directly via biological nitrogen fixation, phosphate solubilization, production of the phytohormone indole-3-acetic acid (IAA), and release of siderophores that enable iron acquisition. They can also offer protection against insect herbivores and phytopathogens, including bacteria, fungi, nematodes, and viruses. This is accomplished by the production of a variety of antimicrobials and insecticides, and by triggering a hypersensitive defensive response of the plant, known as induced systemic resistance (ISR). *Paenibacillus*-derived antimicrobials also have applications in medicine, including polymyxins and fusaricidins, which are nonribosomal lipopeptides first isolated from strains of *Paenibacillus polymyxa*. Other useful molecules include exo-polysaccharides (EPS) and enzymes such as amylases, cellulases, hemicellulases, lipases, pectinases, oxygenases, dehydrogenases, lignin-modifying enzymes, and mutanases, which may have applications for detergents, food and feed, textiles, paper, biofuel, and healthcare. On the negative side, *Paenibacillus larvae* is the causative agent of American Foulbrood, a lethal disease of honeybees, while a variety of species are opportunistic infectors of humans, and others cause spoilage of pasteurized dairy products. This broad review summarizes the major positive and negative impacts of *Paenibacillus*: its realised and prospective contributions to agriculture, medicine, process manufacturing, and bioremediation, as well as its impacts due to pathogenicity and food spoilage. This review also includes detailed information in Additional files 1, 2, 3 for major known *Paenibacillus* species with their locations of isolation, genome sequencing projects, patents, and industrially significant compounds and enzymes. *Paenibacillus* will, over time, play increasingly important roles in sustainable agriculture and industrial biotechnology.

**Electronic supplementary material:**

The online version of this article (doi:10.1186/s12934-016-0603-7) contains supplementary material, which is available to authorized users.

## Introduction to *Paenibacillus*

Bacteria belonging to the genus *Paenibacillus* have been isolated from a variety of environments, with many of the species being relevant to humans, animals, plants, and the environment. The majority of them are found in soil, often associated with plants roots: these rhizobacteria promote plant growth and can be exploited for use in agriculture. Many species of *Paenibacillus* produce antimicrobial compounds that are useful in medicine or as pesticides, and many yield enzymes that could be utilized for bioremediation or to produce valuable chemicals. Some species are pathogens to honeybees or other invertebrates; while others are occasional opportunistic infectors of humans. In fact, many of these pertinent characteristics overlap within the same species. In accordance with their diverse characteristics, members of *Paenibacillus* have been discovered in disparate habitats, from polar regions to the tropics, and from aquatic environments to the driest of deserts (Additional file 1).

Species of *Paenibacillus* were originally included in the genus *Bacillus*, which historically was defined based on morphological characteristics in common with the type species *Bacillus subtilis*, isolated in 1872. Any bacterium was classified as *Bacillus* if it was rod-shaped, aerobic or facultatively anaerobic, and could form endospores allowing it to remain dormant in inhospitable conditions. However, these characteristics are actually very ancient and not suitable for grouping species into a single genus [1]. A study in 1988 using numerical taxonomy based on 188 unit characters suggested a framework for splitting *Bacillus* into several genera [2]. A more accurate representation of phylogenetic relationships among these bacteria was attained in 1991, when 16S rRNA gene sequences were determined for standard strains of 51 species then defined as *Bacillus* [2, 3]. Phylogenetic analyses showed that these sequences segregated into at least five distinct clusters, one of which was reassigned to the novel genus *Paenibacillus* in 1993 [4] and includes the type species *Paenibacillus polymyxa* [5]. The name *Paenibacillus* is derived from the Latin adverb *paene*, meaning almost; almost a *Bacillus* [4].

Not long after the creation of the genus, organisms previously thought of as separate *Paenibacillus* species were re-classified as equaivalent: for example, *Paenibacillus gordonae* was determined to be a synonym of *Paenibacillus validus*, and *Paenibacillus pulvifaciens* was determined to be a subspecies and later a synonym for *Paenibacillus larvae* [6, 7]. Several species were also reclassified into the genus *Paenibacillus*, including *Clostridium durum*, *Bacillus alginolyticus*, *Bacillus chondroitinus*, *Bacillus curdlanolyticus*, *Bacillus glucanolyticus*, *Bacillus kobensis*, and *Bacillus thiaminolyticus* [8, 9]. In 1997, a proposed emendation described *Paenibacillus* as having 16S rDNA sequences with more than 89.6% similarity, being motile by means of peritrichous flagella (projecting in all directions), and being non-pigmented on nutrient agar, among other characteristics. Species of this genus can be gram positive, gram negative, or gram variable, in addition to sharing the basal characteristics ascribed to *Bacillus* [8].

The number of useful or otherwise relevant *Paenibacillus* species has spawned genome sequencing of 212 strains representing 82 species, as well as 79 uncharacterized strains (Additional file 2), and a variety of patents (Table 1). Genome size ranges from 3.02 Mbp (for *P. darwinianus* Br, isolated from Antarctic soil [10]) to 8.82 Mbp (for *P. mucilaginosus* K02, implicated in silicate mineral weathering [11]) and genes number from 3064 (*P. darwinianus* Br) to 8478 (*P. sophorae* S27, a rhizobacterium). Like *P. darwinianus*, the insect pathogens *P. larvae* and *P. popilliae* have genomes on the smaller side (4.51 and 3.83 Mbp, respectively), perhaps reflecting their niche specialization. The DNA G + C content of *Paenibacillus* ranges from 39 to 59 mol% [12].

**Table 1:** Selection of active patents

Patent number	Patenting country	Patenting agency	Patent title	Issue date
US9017692B2	United States	Ohio State Innovation Foundation	Antimicrobial agent, bacterial strain, biosynthesis, and methods of use	28-Apr-15
US8822179B2	United States	University of Florida Research Foundation, Inc.	Nucleic acid compositions and the encoding proteins	21-Feb-12
US8652819B2	United States	University of Georgia Research Foundation, Inc.	*Paenibacillus* spp. and methods for fermentation of lignocellulosic materials	18-Feb-14
US8329430B2	United States	Korea Research Institute of Bioscience and Biotechnology	Polymyxin synthetase and gene cluster thereof	11-Dec-12
US8084418B2	United States	Dow AgroSciences LLC	Methods of inhibiting insects by treatment with a complex comprising a *Photorhabdus* insecticidal protein and one or two Xenorhabdus enhancer proteins	27-Dec-11
US7935335B2	United States	Kaken Pharmaceutical Co., Ltd.	Strains belonging to the genus *Paenibacillus* and method of controlling plant disease by using these strains or culture thereof	03-May-11
CN104255816A	China	Qingdao Jinxiu Shuiyuan Commerce And Trade Co Ltd	Non-polluted antibacterial peptide biopesticide	07-Jan-15
KR1020140115022	Korea	MOS Co., Ltd	Novel *Paenibacillus* peoriae gsoil 1119 having an excellent antifungal effect against plant pathological fungi, and microorganism preparation for controlling containing same	30-Sep-14
WO2014146881A1	International	Wacker Chemie AG	Microorganism strain and method for the fermentative production Of C4 compounds from C5 sugars	27-Feb-14
CN201410255003	China	Stanley Fertilizer Stock Co., Ltd.	Special functional biological slow-release fertilizer for chilies	13-Aug-14
CN201410181469	China	Shandong University	Mixed flora microbial preparation and application thereof in sewage treatment	16-Jul-14
KR1020130055820	Korea	Ecodream Farm Service Corporation	*Paenibacillus* Kribbesis T9 having clubroot control effect for cabbage	04-Jul-14
WO2014099525A1	International	Danisco US Inc	*Paenibacillus* curdlanolyticus amylase, and methods of use, thereof	26-Jun-14
CN201210538699	China	Henan Academy Of Agricultural Sciences	Bio-control bacterium for preventing and treating sesame wilt disease, separation method, inoculant, and application of the inoculant	18-Jun-14
CN201410006084	China	Northeastern University	Method for removing heavy metal ions in water by using fermentation broth of bacterium producing flocculant	04-Jun-14
CN201410018001	China	Yancheng Institute Of Technology	Gene engineering bacteria for producing ultrahigh-optical purity R,R-2,3-butanediol as well as construction method and application thereof	30-Apr-14
CN201310579323	China	Tianjin Wuqing District Plant Protection Station	Biological straw decomposition agent and preparation method thereof	05-Mar-14
KR1020120076529	Korea	Korea Research Institute Of Bioscience And Biotechnology	Method for increasing potato production using novel *Paenibacillus* Sp.	22-Jan-14
KR1020120064809	Korea	Geon-Nong Co., Ltd.	Deodorant for decreasing bad smell of septic tank, garbage dump, sewerage, and compost producing apparatus using complex microbial agent	27-Dec-13
KR1020120022400	Korea	Moon, Byung Woo Yeongam Fig Cluster Agency	Nutrient solution composition for box culturing ficus carica capable of easily adjusting electrical conductivity (ec) and ph according to the amount of the nutrient composition and using method thereof	13-Sep-13
CN201210212246	China	Taicang Zhoushi Chemical Product Co., Ltd.	Method for producing ethanol by adopting mixed culture organism by means of glycerol fermentation	03-Oct-12
KR1020100133442	Korea	Konkuk University Industrial Cooperation Corp.	Novel strain *Paenibacillus* xylanexedens Sk2925 With excellent activity of decomposing xylan	03-Jul-12
JP2009004013	Japan	Yan; Sun Chan	Wastewater treatment method	22-Jul-10
JP2004059241	Japan	Kubota Corp	Method for cleaning contamination caused by polychlorinated biphenyls/polycyclic aromatic hydrocarbons	15-Sep-05
EP1490468	Germany	Aladin Gesellscaft für Innovative mikrobiologische Systeme GmbH	Biological pipe cleaner	29-Dec-04
DE19706598A1	Germany	Knoell Hans Forschung EV Univ Schiller Jena	Macrolide antibiotic apidiothricin	27-Aug-98
WO2016020371A1	Germany	BASF SE	Antifungal *Paenibacillus* strains, fusaricidin-type compounds, and their use	11-Feb-16
WO2016019480A1	Chile	Universidad de Santiago de Chile	*Paenibacillus* polymyxa SCHC 33 bacterial strain, and use thereof to combat phytopathogenic fungi in fruits, vegetables, or plants	11-Feb-16
WO2016019480A1	United States	Danisco US Inc.	*Paenibacillus* and *bacillus* spp. mannanases	17-Mar-16
EP2871073A1	Germany	Roche Diagnostics Gmbh	Recombinantly produced neutral protease originating from *Paenibacillus polymyxa*	16-Jan-14
WO2015153956A1	United States	The Board of Regents of the Nevada System of Higher Education on Behalf of the University of Nevada, Las Vegas	*Paenibacillus larvae* treatment with phage lysin for American Foulbrood Disease	08-Aug-15
WO2015092548A3	Denmark	Dupont Nutrition Biosciences Aps	*Paenibacillus* strains and compositions thereof that inhibit microorganisms	17-Sep-15
WO2015101116A1	China	Bright Dairy & Food Co., LTD	New *Paenibacillus* sp. strain, as well as culture method and use thereof	07-Jul-15
US9085789B2	United States	University of Georgia Research Foundation Inc.	*Paenibacillus* spp. and methods for fermentation of lignocellulosic materials	18-Feb-2014
EP2999801A1	United States	Biowish Technologies, Inc.	Microbial-based waste water treatment compositions and methods of use thereof	30-Mar-2016
EP2999340A1	Japan	Hisataka Goda	Drug-resistant microbe and variant microbe disinfectant containing chlorous acid aqueous solution	30-Mar-2016
WO2016054176A1	United States	Danisco US Inc.	Compositions comprising beta-mannanase and methods of use	07-Apr-2016
US8956842B2	Korea	Korea Research Institute of Chemical Technology	*Paenibacillus* sp. HPL-3 strain producing xylanase having heat-resistance, a wide range of optimum pH and high activity, a novel xylanase separated from the strain, and a method for mass-production of the same using the transformant originated from the strain	17-Feb-2015
EP2929022A1	United States	Danisco US Inc.	Compositions and methods of use	14-Oct-2015
EP2759600A4	Japan	Hayashibara Co	Production method for powder containing crystalline, -trehalose dehydrate	08-Jul-2015
EP2556835A1	Japan	Japan Eco-Science Co. Ltd.	Mixture, dissolving solution and pharmaceutical agent each comprising thermophilic microorganism	13-Feb-2013
WO2015114552A1	South Africa	University of Pretoria	Plant growth promoting rhizobacterial strains and their uses	06-Aug-2015
WO2015123752A1	Canada	Green On Industries, Inc.	Composition and method for hydrocarbon and lipid degradation and dispersal	27-Aug-2015
WO2015109766A1	China	Bright Dairy & Food Co., Ltd.	Method for preparing fermentation broth extracts having chymosin activity and products thereof	30-Jul-2015
WO2015113175A1	Chile	Universidad Del Desarrollo	Biocide composition for controlling pests affecting European honey bees, comprising a water-soluble *Olea europaea* extract	06-Aug-2015
WO2015023662A1	United States	Bio-Cat Microbials Llc	Compositions comprising bacillus strains and methods of use to suppress the activities and growth of fungal plant pathogens	19-Feb-2015
US9187381B1	United States	Bio AG Corp.	Composition and method for formulating a biofertilizer and biopesticide	17-Nov-2015
US9113605B2	United States	Core Intellectual Properties Holdings, Llc	Methods and compositions to aggregate algae	25-Aug-2015

Patents were searched at the World Intellectual Property Organization (WIPO), European Patent Office (EPO), United States Patent and Trademark Office (USPTO), and Japan Patent Office (JPO)

Currently, *Paenibacillus* is one of eight genera included in the family Paenibacillaceae. However, a phylogram of the family suggests that *Paenibacillus* is paraphyletic, with the other genera (*Aneurinibacillus*, *Brevibacillus*, *Cohnella*, *Fontibacillus*, *Oxalophagus*, *Saccharibacillus*, and *Thermobacillus*) forming subsidiary clades. The genus *Paenibacillus* is therefore expected to undergo significant taxonomic subdivision in the future [1]. Conversely, the number of novel species being identified as *Paenibacillus*, and established species being reclassified as such, continues to grow, and the genus currently comprises around 200 species (Additional file 1). Despite this complexity, the present review attempts to summarize all members currently classified as *Paenibacillus* with respect to the characteristics—both positive and negative—that are most relevant to humankind.

## Plant growth promotion

The genus *Paenibacillus* contains many species which are known to promote the growth of plants including maize [13], *Populus* [14], pumpkin [15], rice [16], switchgrass [17], and many others. Like other plant growth-promoting bacteria, they accomplish this through various facets. Plant-associated species of *Paenibacillus* can directly influence plant growth by producing indole-3-acetic acid (IAA) and other auxin phytohormones, solubilizing inaccessible phosphorous into form that can be taken up by plant roots, and some species can also fix atmospheric nitrogen [18]. In addition, *Paenibacillus* helps to control phytopathogens by triggering induced systemic resistance (ISR) and/or producing a variety of biocidal substances (see “Biocontrol” and “Antimicrobial peptides” sections).

While some plant growth promoting bacteria, including *P. macerans*, are used in commercial biofertilizers, their use is currently limited. The establishment and performance of these microorganisms in the field can be affected by numerous environmental variables, such as soil pH, salinity, moisture content, and temperature [19]. Despite these limitations, continuing research may enable more widespread use of these biofertilizers. One benefit of inoculating fields with endospore-forming bacteria such as *Paenibacillus* is their capacity to survive for long periods in the soil under adverse environmental conditions [20].

### Nitrogen fixation

Atmospheric nitrogen (N_2_) is relatively inert, and must be fixed to a usable chemical form before being incorporated into amino acids, nucleotides, and other metabolites. As eukaryotes do not have the ability to fix their own nitrogen, its bioavailability in the soil is a major limiting factor for plant growth, and farmers routinely apply nitrogen fertilizers to ensure crop productivity. Commercial nitrogen fertilizers are produced by the Haber-Bosch process through reducing nitrogen gas, which uses fossil fuels for the energy needed, and the resulting carbon dioxide emissions and pollution contributing to global warming and adverse effects on human health. Additionally, over 50% of synthetic nitrogen fertilizer is not taken up by crops, and is instead lost to the environment where it contributes to eutrophication, greenhouse gas production, and acid rain [21]. Such nitrogen pollution is considered to be the second most important driver of anthropogenically induced global change, next to the perturbation of the carbon cycle [22]. However, its effects can be lessened by inoculating fields or crops with microorganisms, including some strains of *Paenibacillus*, that fix nitrogen in or around plant roots, where it is actually needed.

Nitrogen-fixing (diazotrophic) bacteria and archaea primarily use a molybdenum (Mo)-dependent nitrogenase (Nif) to catalyze the reduction of N_2_ to bioavailable NH_3_. Synthesis of this enzyme requires a minimum of three structural genes and three genes for FeMo-cofactor biosynthesis [23]. However, optimum activity requires the presence of additional genes, as the specific activity of a nitrogenase expressed in *Escherichia coli* was only about 10% of that observed in the *Paenibacillus* species from which it was derived; and improvements were made with additional transgenes [24]. Alternatives to Nif have active site cofactors that lack Mo, instead containing both vanadium and iron (Vnf), or iron only (Anf) [23].

More than 20 *Paenibacillus* species can fix nitrogen [25], with single species comprising both diazotrophic and non-diazotrophic strains [26, 27]. The *nif* gene cluster is highly conserved among nitrogen-fixing *Paenibacillus*, with most clusters containing 9 genes within 10.5–12 kb, and exhibiting over 80% identity [25]. Most of the Nifs appear monophyletic, having likely been derived from a single horizontal gene transfer, followed by duplications and gene cluster loss in some lineages [27, 28]. At least two strains, *P. riograndensis* SBR5^T^ and *P. durus* DSMZ1735, have additional *nif*s from a potentially independent, more recent horizontal transfer, although these genes may not be functional [28]. The alternative nitogenase Vnf is encoded in the genomes of *P. zanthoxyli* JH29 and *P. azotofixans* ATCC 35681, while Anf is encoded by *P. sophorae* S27, *P. forsythia* T98 [25], and *P. riograndensis* SBR5^T^; all of which also contain the *nif* cluster. Having Vnf or Anf, in addition to Nif, may provide an selective advantage in certain circumstances, as *anf* shows higher expression under molybdenum-depleted conditions [28].

The ability of organisms to fix nitrogen can be identified by growth on nitrogen-free medium, while reliable estimates of nitrogenase activity can be obtained using ^15^N_2_ fixing assays. However, acetylene reduction assays are less costly than ^15^N_2_ and are sufficient to estimate relative nitrogenase activities. These assays are based on the ability of nitrogenases to also reduce acetylene (C_2_H_2_) to ethylene (C_2_H_4_). Such assays have found a wide range of relative activities among nitrogen-fixing *Paenibacillus* species, for example, with *P. zanthoxyli* DSM 18202 producing 140 times more activity than *P. peoriae* DSM 8320 [29].

### Phosphate solubilization

Next to nitrogen, phosphorus is the second most important element limiting plant growth and productivity. Although it is abundant in soils, only 0.1% exists in a soluble form that can be taken up by plant roots; the remainder forms insoluble mineral complexes, or is immobilized in organic matter. Chemical phosphorus fertilizers are therefore used to supplement soluble phosphorus on most agricultural soils, but these are costly and adversely impact the environment [19].

Unlike nitrogen, phosphorus fertilizer is a finite resource obtained from rock phosphate, and once high-quality deposits are used, the shift to lower grade rock will result in even higher costs. The manufacturing process emits poisonous hydrogen fluoride gas. Once applied to the field, less than 30% of the chemical fertilizer is typically used by the plant, with much of the rest incorporating into inorganic mineral complexes within the soil. The rise in insoluble phosphorus can disturb microbial diversity, eventually leading to reduced soil fertility; and trace amounts of heavy metal impurities in the fertilizer can accumulate over time. Eutrophication can also result as phosphorus-rich soil erodes into aquatic systems [19].

As is the case for nitrogen, the application of chemical phosphorus fertilizer could be reduced by inoculating fields with phosphorus-solubilizing microorganisms, such as *Paenibacillus*. While bacteria may have drawbacks when compared to phosphorus solubilizing fungi, such as lower activity and a tendency to lose activity after repeated sub-culturing [19], some may nonetheless become preferred bio fertilizers due to additional qualities that simultaneously benefit plant growth.

Phosphorus solubilizing microorganisms use a variety of mechanisms that make phosphorus available to plants, with the principal method being organic acid (especially gluconic acid) production. Such acids can directly dissolve mineral phosphorus through anion exchange, chelate metal ions, or lower soil pH to release phosphorus from the mineral complex. Microorganisms also release phosphorus from their substrates during enzymatic degradation, and from their own cells upon death [19].

Genomic analyses suggest that most *Paenibacillus* strains can solubilize phosphorus through gluconic acid production: a study of 35 strains comprising at least 18 species found that all but two strains have genes involved in gluconic acid production, encoding glucose-1-dehydrogenase and gluconic acid dehydrogenase. The strains apparently lacking these genes were *P. beijingensis* 1–18 and *P. terrae* HPL-003; while those having the genes included strains from *P. azotofixans*, *P. curdlanolyticus*, *P. dendritiformis*, *P. elgii*, *P. forsythia*, *P. graminis*, *P. lactis*, *P. massiliensis*, *P. mucilaginosus*, *P. peoriae*, *P. polymyxa*, *P. sabinae*, *P. sonchi*, *P. sophorae*, *P. vortex*, and *P. zanthoxyli* [27]. Genes for the uptake and degradation of phosphonates, which containing the highly stable C-P bond, and for a phosphate-specific transport system, were found in all analysed genomes [27]. Phosphorus solubilisation has been confirmed by *P. elgii* [30], *P. kribbensis* [31], *P. macerans* [32], *P. mucilaginosus* [33], *P. polymyxa* [32], *P. xylanilyticus* [34], and several unclassified strains.

### Iron acquisition

Like phosphorus, iron is abundant in soil in mainly in non-bioavailable form. Particularly in alkaline or chalky soils, it forms largely insoluble Fe^3+^ oxy-hydroxides, which are not readily used by either microorganisms or plants. Most microorganisms therefore reduce Fe^3+^ to Fe^2+^ using ferrireductases or solubilize it with extracellular, low molecular weight Fe^3+^ chelators called siderophores [35], which are released under iron limited conditions. The soluble Fe^3+^-siderophore complexes are available to plants as well as microorganisms [36], being recognized by specific membrane receptors and transported into cells [37].

Siderophores are synthesized mainly by nonribosomal peptide synthetases (NRPSs), which are encoded by gene clusters. These multienzyme complexes consist of various modules that each incorporate one or more specific amino acids into a peptide backbone (see also “Nonribosomal lipopeptides” section). The three types of siderophores are classified based on their functional groups, being catecholates, hydroxamates, and α-hydroxy carbolates [38]. However, few siderophores have thus far been characterized from *Paenibacillus*.

*Paenibacillus larvae* produces a catecholate -type siderophore called bacillibactin, which is also made by *Bacillus subtilis* and members of the *Bacillus cereus* sensu lato group [38]. Bacillibactin is a cyclic trimeric lactone of 2,3-dihydroxybenzoate (DHB)-glycine-threonine. Similar to bacillibactin, paenibactin is a cyclic trimeric lactone of 2,3-DHB-alanine-threonine produced by *P. elgii* B69, the difference being the amino acid inserted between the DHB and threonine units [37]. Catecholates have not been detected in cultures of *P. polymyxa* SQR-21, which instead produces hydroxamate-type siderophores at low concentrations in the late log phase [35]. Siderophore synthesis gene clusters are present only in some strains of some *Paenibacillus* species, and are thought to have been obtained from fairly recent events of horizontal gene transfer [26, 37].

Aside from siderophore production, *Paenibacillus* may promote iron uptake by plants via other mechanisms. *P. polymyxa* BFKC01 transcriptionally activates plant genes involved in iron deficiency responses, including the membrane bound ferric chelate reductase *fro2* and the divalent metal transporter *irt1*, as well as genes involved in the synthesis of iron-mobilizing phenolic compounds [39]. *Paenibacillus*-produced organic acids, such as oxalic acid, could also conceivably contribute to iron uptake, but release of such acids does not appear to be iron regulated [35].

### Phytohormone production

Auxins are hormones that are crucial regulators of gene expression and development throughout a plant’s life, participating in cell division, elongation, fruit development and senescence. There are multiple classes of auxins, but the first identified and most abundant in nature is indole-3-acetic acid (IAA) [40]. Although plants are able to produce their own phytohormones, they can also utilize foreign sources produced by other organisms.

In addition to plants, IAA is synthesized by fungi and bacteria, including *Paenibacillus* and most other plant-associated bacteria [20]. The production of IAA by *Paenibacillus* is thought to contribute to plant growth promotion [41]. However, this hormone has a complex relationship with plants, being produced by both plant-growth-promoting and phytopathogenic bacteria. Lower levels of exogenous IAA typically increase plant growth and productivity, while high levels lead to disease susceptibility [20]. Perhaps auspiciously, the tryptophan precursor of IAA is energetically costly, and considerable amounts are only made with an excess of tryptophan, which may be exuded from the plant, typically during the stationary phase of bacterial growth [41].

Three different enzymatic pathways have been identified that convert tryptophan to IAA; in bacteria, the most common is the indole-3-pyruvic acid pathway, which is also present in plants. In this pathway, an aminotransferase deaminates tryptophan to yield indole-3-pyruvic acid, which is then converted to indole-3-acetaldehyde by a decarboxylase in the rate-limiting step. Finally, indole-3-acetaldehyde is oxidized to IAA via an unknown enzyme [20, 41].

Genes encoding putative indolepyruvate decarboxylase (IpdC), a key enzyme in the indole-3-pyruvic acid pathway, are present in all analyzed *Paenibacillus* genomes, with over 96% amino acid identity between strains across 98% of the sequence [27]. IpdC belongs to a family of enzymes that exhibit some level of substrate promiscuity, and substitution of only a few amino acids in the active site can shift the primary affinity to another substrate. The active site must therefore be analysed in detail to confirm a gene’s identity as an *ipdC* homologue whose primary function is IAA synthesis [41]. However, the *Paenibacillus* genomic analysis did not identify genes involved in the other IAA pathways, that is, tryptophan monooxygenase or indole-3-acetamide hydrolase, suggesting that this genus most likely relies on the indole-3-pyruvic acid pathway [27].

## Biocontrol

Perhaps the most notable plant-growth promoting feature of *Paenibacillus* species comes from their numerous biocontrol capabilities. By inducing the plant’s own resistance mechanisms or by producing biocidal substances, *Paenibacillus* can neutralize a diverse variety of phytopathogens and insect herbivores. *P. polymyxa* alone has been shown to provide protection to cauliflower [42], pea [43], ginseng [44], cucumber [45], chickpea [46], peanut [47], soybean [48], pepper [49], and more. Other species of *Paenibacillus* that have biocontrol properties include *P. alvei* [50], *P. brasilensis* [51], *P. dendritiformis* [52], *P. ehimensis* [53], *P. elgii* [54], *P. kobensis* [55], *P. lentimorbus* [56], *P. macerans* [57], *P. peoriae* [58], and *P. thiaminolyticus* [59].

Tolerance of some *Paenibacillus* species to commercial fungicides and insecticides [60] indicates the possibility of using these microorganisms in combination with existing control solutions. However, when considering *Paenibacillus* or its compounds for biocontrol, it is important to note that competition can occur both ways. For example, while low levels of fusaric acid, a toxin produced by the fungus *Fusarium oxysporum*, increase production of the antifungal enzyme β-1,3-glucanase by *P. polymyxa* strains WR-2 and SQR-21, higher levels of fusaric acid actually decrease their production and result in reduced *P. polymyxa* growth [61]. Furthermore, *Paenibacillus* may compete with other beneficial organisms [62, 63].

### Induced systemic resistance

Many beneficial rhizobacteria and root-associated mutualists, including members of *Paenibacillus*, can trigger induced systemic resistance (ISR) when present in high enough population densities. ISR is a latent defense mechanism occurring in plant tissues that are spatially separated from the inducer, providing enhanced protection against a range of pathogens or pests. Rather than immediately activating a defensive state, ISR primes for faster and stronger defenses by hypersensitizing the plant to potential threats [64]. A variety of *Paenibacillus* species seem to elicit ISR against pathogenic bacteria [49, 65], fungi [50, 66–68], nematodes [69], and viruses [70].

The ISR pathway begins when the plant recognizes elicitors from the beneficial microorganism, such as structural proteins, enzymes, reactive oxygen species, or volatile organic compounds [64, 71]. ISR can lead to increased systemic levels of the plant hormone salicyclic acid (SA-dependent response), or to an SA-independent response. The latter can include increased transcription of genes that are regulated by the plant hormones jasmonic acid, or enhanced expression of genes that are responsive to jasmonic acid or ethylene, which are then induced upon attack. In addition, SA-independent ISR can prime for physical responses such as enhanced callose deposition at sites of pathogen entry, which is regulated by abscisic acid and creates a structural barrier against further attack. While there are exceptions, SA-dependent ISR typically induces mechanisms against biotrophic pathogens (those requiring that the host cells remain alive), while the jasmonic acid/ethylene pathway protects against cell death-provoking necrotrophs and against insect herbiviores [64].

Among *Paenibacillus* species, ISR has been demonstrated for *P. polymyxa*, *P. alvei*, *P. elgii*, and *P. lentimorbus*. For example, *P. polymyxa* strain KNUC265 was shown to protect against the bacterial pathogens *Xanthomonas axonopodis* and *Erwinia carotovora* in pepper and tobacco, respectively, using bacterial volatiles and diffusible metabolites as elicitors [49]. *P. polymyxa* E681 was shown to use volatile organic compound elicitors to protect *Arabidopsis thaliana* against the bacterium *Pseudomonas syringae* via primed transcription of salicylic acid, jasmonic acid, and ethylene signaling genes [65]. Consistent with the typical roles of SA-dependent and jasmonic acid/ethylene pathways, *Pseudomonas syringae* is described as a hemibiotrophic pathogen, with both biotrophic and necrotrophic stages [72]. Similarly, *Arabidopsis thaliana* is primed by *P. alvei* K165 against a hemibiotrophic fungus [54], *Verticillium dahlia*, by both salicylate and jasmonate-dependent pathways [68]; while cucumber is primed by *P. elgii* MM-B22 against the hemibiotrohic fungus [54] *Colletotrichum orbiculare*, responding to attack with defense-related enzymes and H_2_O_2_-induced cell death [67]. Priming of tobacco by *P. lentimorbus* B-30488 also results in accumulation of defense-related enzymes in response to cucumber mosaic virus infection [70].

### Insecticides

*Paenibacillus* species have been shown to kill larvae of pest insects including beetles [73] and lepidopterans [74]. *Paenibacillus popilliae*, which infects larvae of the Japanese beetle, *Popillia japonica*, was the first microbial control agent registered in the US for use against an insect. However, its use was never widespread due in part to the inability of this particular species to be cultured in synthetic media [75]. Nonetheless, research continues into the potential of *Paenibacillus* species for insect pest control, with the effective proteins including chitinase and crystal protein (Cry).

Chitinase enzymes produced by *Paenibacillus* hydrolyse chitin, which is a structural polysaccharide of insect exoskeletons and gut linings, leading to low feeding rates and death of infected insects. Both *Paenibacillus* sp. D1, from a seafood industry effluent treatment plant, and its isolated chitinase have been shown to cause concentration-dependent mortality of cotton bollworm (*Helicoverpa armigera*) when coated onto leaves fed to the larvae [76]. *Paenibacillus* sp. D1 and its chitinase are tolerant to common insecticidal chemicals, and the chitinase itself is also highly stable in the field at 40 °C [60], indicating the potential of the organism or its enzyme to be used as insecticide in the field.

Cry proteins are best known for conferring insecticidal activity to the bacterium *Bacillus thuringiensis* and to genetically modified crops. Following ingestion, these proteins form pores in the insect midgut epithelial cells, resulting in cell lysis and death [77]. Cry homologs from *Paenibacillus lentimorbus* strain Semadara, which was isolated from larvae of the beetle *Blitopertha orientalis*, have been shown to cause mortality of beetle larvae [78]. Genes encoding Cry have also been found in *P. popilliae* [79] and *Paenibacillus* spp. Kh3 [80]. In addition, *P. polymyxa* NMO10 has been genetically engineered with Cry1C from *B. thuringiensis* in order to combine the insecticidal activity of Cry and the growth promotion properties of *P. polymyxa*. The modified strain demonstrated greater toxicity than *B. thuringiensis* against lepidopteran insects [81, 82].

While insecticidal activities of *Paenibacillus* species may contribute to insect pest control, it is important to note that interactions between species in the field can be complex and non-target effects of biocontrol agents needs to be considered. For example, large populations of *Paenibacillus* species, or *P. polymyxa* alone, near the root system can actually increase the susceptibility of plants to aphids, possibly via increased levels of the plant growth promoting hormone IAA [83]. Furthermore, while various *Paenibacillus* species suppress parasitic nematodes, most notably the root-knot nematode *Meloidogyne incognita*, *P. nematophilus* has been found to impede dispersal of the beneficial nematode *Heterorhabditis megidis* and reduce its infectivity of moth larvae [63].

### Antimicrobials

Many *Paenibacillus* species compete with other microorganisms through the production of a wide range of antimicrobial compounds. In one study, 25 of 55 isolates from water and soil exhibited a broad inhibition spectrum against tested bacteria and pathogenic fungi Lorentz RH, Ártico S, Da Silveira AB, Einsfeld A and Corção G [84], suggesting that a good proportion of *Paenibacillus* species are likely to produce antimicrobials. Yet diversity exists even within the same species: of 25 strains of *P. polymyxa*, 15 were strongly inhibitory to the oomycete pathogen *Phytophthora capsici*, while 10 showed weak or no antimicrobial effect [85]; and genome sequencing confirms the diversity of antimicrobial-encoding gene clusters among *P. polymyxa* strains [26, 27].

Various *Paenibacillus* strains, or their isolated antimicrobial compounds, could therefore be useful in controlling phytopathogenic microorganisms, leading to lower usage of chemical biocides which can have negative environmental effects. Soilborne fungal pathogens, in particular, require high doses of chemical fungicides for control due to their wide host spectra and persistence in soil [53]. The antimicrobial activities of *Paenibacillus* may also be useful for post-harvest control of food-borne bacteria, such as *Salmonella*, that are pathogenic to humans [86].

The antimicrobials produced by *Paenibacillus* include peptides, enzymes, and volatile organic compounds (VOCs). While antimicrobial peptides are extremely significant for biocontrol in agriculture, the purified or synthesized peptides also have realised and potential uses in medicine and food processing, and are therefore discussed further in a separate section (see “Antimicrobial peptides” section).

Hydrolytic enzymes of *Paenibacillus* can attack the cell walls of fungal and oomycete competitors. Cell walls of filamentous fungi contain a large fraction of β-1,3-glucan and chitin, while those of oomycetes consist primarily of β-1,3-glucan, β-1,6-glucan and cellulose; and both contain up to 11% protein. Various species of soil-dwelling *Paenibacillus* have been found to produce glucanses, chitinases, cellulases, and proteases that are implicated in the destruction of eukaryote cell walls. For example, crude enzyme extract from *P. ehimensis* KWN38 was shown to deform hyphal morphology and prevent growth of the basidiomycete fungus *Rhizoctonia solani*, the ascomycete fungus *Fusarium oxysporum*, and the oomycete *Phytophthora capsici*, all of which are phytopathogens [53]. Glucanases and chitinases from strains including *P. ehimensis* IB-X, *P. ehimensis* MA2012, and *P. polymyxa* A21 have been shown to damage cell wall structures and/or inhibit *R. solani*, the oomycete *Pythium aphanidermatum*, and the ascomycetes *Alternaria alternata*, *Botrytis cinerea*, *Colletotrichum gloeosporioides*, and *Drechlera sorokiniana* [87–90]. A chitinase from *Paenibacillus* sp. D1 also exhibited high stability in presence of commonly used fungicides, suggesting the potential of some hydrolytic enzymes as additives to chemical fungicides [91].

VOCs can enhance interactions between soil-dwelling microorganisms, as these compounds diffuse through air-filled pores in the soil to reach physically separated organisms [92]. A large number of VOCs are produced by *Paenibacillus* species [93, 94] as well as other microorganisms [92]. For example, *P. polymyxa* WR-2 was found to produce 42 VOCs, over 30 of which had some degree of antifungal activity against *F. oxysporum*, including 13 that completely inhibited its growth. The compounds included benzenes, aldehydes, keytones, and alcohols, although some were produced in low quantities; with benzothiazole, benzaldehyde, undecanal, dodecanal, hexadecanal, 2-tridecanone and phenol being the main antifungal compounds [94]. Antimicrobial VOCs have application for biocontrol of agricultural pathogens, post-harvest diseases (particularly of fruit), and building molds [92].

In addition to the antimicrobials described above, a non-volatile organic compound, methyl 2,3-dihydroxybenzoate, from *P. elgii* HOA73 was found inhibited growth of *B. cinerea*, *F. oxysporum*. *P. capsici*, and *R. solani* [95].

## Antimicrobial peptides

Further to “Antimicrobials” section, *Paenibacillus* produces antimicrobial peptides with realised or potential applications in agriculture, medicine, and food processing. These peptides are of two types: ribosomally-synthesized bacteriocins, and non-ribosomally synthesized peptides, where amino acids are incorporated independently of messenger RNA.

### Bacteriocins

Bacteriocins are ribosomally synthesized, proteinaceous toxins that inhibit the growth of bacteria that are related to the producer. *Paenibacillus* species are known to produce at least two of the three classes of bacteriocins, being lantibiotics and pediocins.

Lantibiotics, also known as Class I bacteriocins, contain the non-coded amino acid lanthionine [96]. They are typically active against Gram-positive bacteria, as the outer membrane of Gram-negative bacteria presents a natural barrier. However, some gram negatives can be affected at high concentrations [97].

Lantibiotics are usually expressed at the late exponential phase or early stationary phase of bacterial growth, and are encoded in a cluster along with genes required for their extensive post-translational modifications [96]. In *P. polymyxa* OSY-DF, for example, the paenibacillin prepropeptide is encoded in a cluster that also contains putative genes for lantibiotic dehydratase, lantibiotic cyclase, acetylase, peptidase, and an ATP-binding cassette (ABC) transporter that may function for export to the extracellular space [96]. Other lantibiotics include paenicidin A produced by *P. polymyxa* NRRL B-30509 [98] and penisin produced by *Paenibacillus* sp. strain A3 [99].

Lantibiotics in *Paenibacillus* are fairly recent discoveries, with Paenibacillin first reported in 2007 [100]. However, the lantibiotic Nisin, produced by *Lactococcus lactis*, has been in commercial use since the 1950s, both as a food preservative and in veterinary medicine. Lantibiotics have low toxicity toward mammals and are poorly immunogenic, meaning there is little risk of adverse effects [97]. Paenibacillin has the advantages of pH and heat stability, as well as activity against a broad range of foodborne pathogens and spoilage bacteria, making it an attractive candidate for food preservation [101].

Compared to these lantibiotics, less research has been done on *Paenibacillus*-produced pediocins, also known as Class II bacteriocins. Pediocins are nonmodified, linear peptides, and include SRCAM 37 and SRCAM602 produced by *P. polymyxa* [101].

### Nonribosomal lipopeptides

In contrast to bacteriocins, many antimicrobial peptides produced by *Paenibacillus* are synthesized nonribosomally, independently of RNA. Here, the amino acid residues are pieced together by nonribosomal peptide synthetases (NRPSs), which are multienzyme complexes that can incorporate a mixture of d and l amino acids. Each module of an NRSP incorporates one or more specific amino acids into the peptide chain. Resulting peptides show great diversity in sequence and structure, and an enhanced resistance to proteolytic enzymes. The nonribosomal lipopeptides act primarily by disrupting membranes of the target cells, and because it is difficult for target organism to reorganize their membranes, development of resistance is usually slow [102].

Nonribosomal lipopeptides can be categorized as linear cationic, cyclic cationic, or cyclic noncationic. Although they are the most amenable to chemical synthesis, limited research has been conducted on linear cationic nonribosomal lipopeptides. In *Paenibacillus*, these include saltavalin, jolipeptin, and tridecaptins [102].

#### Cyclic cationic lipopeptides

The most thoroughly researched cyclic cationic lipopeptides from *Paenibacillus* are the polymyxins, first isolated from *P. polymyxa* in 1947, although they are also produced by strains of *P. alvei* [103], *P. kobensis* [55], and possibly other species. These are a family of peptides each consisting of a polycationic diaminobutyryl-containing heptapeptide ring and tripeptide side chain with a fatty acid derivative at the N-terminus [104]. Members of the family include polymyxin A, B, C, D, E (also called colistin), M (also called mattacin), P, S, and T [103–106]. These polymyxins differ from each other in amino acid composition at residues 3, 6, and 7, including D vs L stereochemistry of amino acids. Subgroups (e.g. polymyxin E_1_ and E_2_) differ in the lipid moiety and/or the amino acid at residue 7 [103]. Amino acid diversity is thought to arise from combinatorial chemistry, due to mixing and matching of alleles of the NRPS modular domains [104]. Polymyxin gene clusters found to date each encode three multi-modular NRPSs and two ABC transporters [103, 107].

Polymyxins bind to the lipid A component of lipopolysaccharide on the outer membrane of gram-negative bacteria to disrupt the outer membrane, then permeabilize and disrupt the inner membrane. Most cases of resistance occur in strains that have modified lipid A to reduce its net negative charge, thereby reducing affinity for polymyxin [102].

Polymyxins B and E are produced industrially from *P. polymyxa* strains [103]. They are used in ointments, such as the antibiotic creams Neosporin and Polysporin (both contain polymyxin B), for the treatment and prevention of topical skin infections; and as last-resort treatments for multidrug resistant internal infections [108]. While polymyxins were used extensively from the 1940s until the 1970s to treat gram-negative bacterial infections, their clinical uses are currently limited primarily because of toxicity to the human central nervous system and kidneys. While this toxicity may be less severe than previously reported [102], synthetic production with modifications can create new polymyxins with improved pharmacokinetic properties as well as activity against resistant bacteria [109].

Other cyclic cationic lipopeptides produced by *Paenibacillus* include octapeptins (e.g. battacin), paenibacterin, polypeptins (e.g. pelgipeptin), and gavaserin. Octapeptins have the structure of truncated polymyxins, but are active against both Gram-negative and Gram-positive bacteria, and are less toxic [102]. Paenibacterin is a cyclic 13-residue amino acid produced by *P. thiaminolyticus* OSY-SE, with activity against Gram negative and positive bacteria [59].

#### Cyclic noncationic lipopeptides

The cyclic noncationic lipopeptides found so far in *Paenibacillus* are fusaricidins, first reported in *P. polymyxa* KT-8 in 1996 [110]. These are hexapeptide rings that contain one or more ester bonds in addition to the amide bonds (depsipeptides), with an attached guanidinylated ß-hydroxy fatty acid [104, 111]. A single operon produces a variety of fusaricidins, differing in their incorporation of amino acids at three of the six positions in the peptide ring. The diversity here is due to relaxed substrate specificity of the NRPS [112], in contrast to the modular mixing of polymyxin synthases. Fusaricidins are active against fungi, including many important phytopathogens, and a variety of gram-positive bacteria. Both naturally occurring structures and synthetic modifications can be chemically synthesized, creating improved stability and decreased nonspecific cytotoxicity toward human cells [111].

## Other medical applications

In addition to a diverse array of antimicrobials, *Paenibacillus* produce other compounds that may be useful in medicine and dentistry. Their exo-polysaccharides (EPS) have antioxidant and anti-tumour properties, while mutanase enzymes may help to reduce tooth decay.

Microbial EPSs are water-soluble polymers that attach to the cell surfaces or are released into the medium. Strains of *Paenibacillus* produce EPSs with varying characteristics that may be medically useful. For example, those from *P. polymyxa* SQR-21 and *P. polymyxa* EJS-3 have superoxide scavenging activity and inhibit lipid peroxide [113, 114]. Some of these EPSs have been found to reduce oxidative stress in the livers of mice and inhibit in vitro growth of gastric cancer cells [114].

Mutanases, also called (1→3)-α-glucanases, from *Paenibacillus* may be useful to help prevent tooth decay. These enzymes break down branched (1→3),(1→6)-α-D-glucans (mutans) which are produced by commensal streptococci and which form a major component of dental biofilm (plaque) that can harbor cariogenic bacteria. In contrast to the other polysaccharides and proteins in the biofilm, mutans are resistant to enzymes produced by oral microorganisms, and are substantially rigid and water-insoluble, thus are not dissolved and washed away by oral fluid [115].

Mutanases are produced by fungi and bacteria, including *P. curdlanolyticus*, *P. glycanilyticus*, and *P. humicus*. However, these enzymes are not widespread in nature and are not produced by oral microorganisms, with producing bacteria usually isolated from soil. The bacterial enzymes are typically endo-(1→3)-α-glucanases, which cleave internal (1→3)-α-linkages at random sites along the glucan chain. These mutanases tend to be more stable than their fungal counterparts, and are active at a higher pH that is more consistent with the oral environment [115].

A marketed oral rinse, Biotene PBF (Laclede Professional Products), contained mutanase among other components, but the product’s effectiveness was not proven, which may be due to a variety of reasons. In many cases, mutanase production requires inducing (1→3)-α-glucans, which have been difficult to make in quantities large enough to support products with high enzyme concentrations. However, (1→3)-α-glucan components of fungal cell walls have more recently been shown to induce mutanase expression in *Paenibacillus* and other organisms [115].

## Process manufacturing

*Paenibacillus* strains produce a variety of enzymes with potential applications in industrial process manufacturing for detergents, food, textiles, paper, and biofuel; including amylases, cellulases, hemicellulases, lipases, pectinases, and lignin-modifying enzymes (Additional file 3). Although enzymes sourced from *Paenibacillus* are not presently used in these processes, the search is ongoing for enzymes that are highly active under industrially-relevant conditions, have improved stability, or can be produced at a lower cost than currently available alternatives.

The laundry and dish detergents industry is the primary consumer of industrial enzymes. Proteases, lipases, amylases, and sometimes hemicellulases, are used to break down food and other organic residues, such as blood and grass stains. Cellulases are also used in laundry detergents to restore the smooth look and feel cotton-based fabric, by removing small balls of fibers that form on the cloth during wearing and washing. The other major industry to use enzymes is food, feed, and beverages. Here, amylases convert starches into sugar sweeteners such as high-fructose corn syrup, and create precursors for brewing alcoholic beverages. Cellulases and pectinases are used to extract and clarify juices, and, along with hemicellulases, to improve the nutritional quality of animal feeds. In the textile industry, pectinases, proteases, and lipases remove impurities from cotton and enhance wettability for dyeing and finishing; amylases remove coating agents from yard after it is woven (desizing); and cellulases can produced a “stonewashed” denim finish. For paper products and cellulosic biofuel, microbial enzymes can help remove lignin that causes paper to yellow and reduces the availability of fibers for saccharification and fermentation to biofuels. After lignin removal, cellulases and hemicellulases can alter fiber properties for paper manufacturing, or hydrolyze fibers for bioethanol or biobutanol production. For an alternative biofuel, lipases can serve as transesterification catalysts for biodiesel production [116].

Cold-active enzymes are desirable for laundry detergents, as washing with cold water reduces energy consumption and fabric wear. Fittingly, cold-active protease, amylase, xylanase, and cellulase are efficiently produced by *P. terrae* [117]. For most other applications, thermostability and activity under harsh conditions are desired. Accordingly, a cellulase from *P. chitinolyticus* CKS1 has optimal activity at 80 °C and pH 4.8 [118]; while another from *P. tarimensis* retains high activity from pH 3.0 to 10.5, from 9 mM to 5 M NaCl, at 80 °C in high salt, and in the presence of organic solvents, EDTA, and heavy metals [119].

## Bioremediation

A variety of industries including petroleum, textiles, pulp and paper, and other chemical industries can unintentionally or intentionally release large amounts of organic pollutant compounds and heavy metals. *Paenibacillus* species may be utilized in the removal or degradation of these environmental pollutants, through bioflocculation or enzymatic activities.

Often used for wastewater treatment, flocculation is a process that removes suspended particles from liquids, frequently through the addition of chemicals (flocculants) that promote aggregation. Many microorganism including bacteria, fungi, and algae can produce biological flocculants consisting of polysaccharides, proteins, or other macromolecules. Strains of *P. jamilae*, *P. macerans*, *P. polymyxa*, and *P. validus* have been shown to promote bioflocculation of heavy metal ions or acid dyes; and *P. elgii* B69 produces an exopolysaccharide (EPS) bioflocculant that can remove multiple pollutants including heavy metal ions, dyes, and kaolin clay over a wide pH range [120]. *Paenibacillus* could therefore be used to help remove contaminants from a variety of wastewaters.

To degrade contaminants, either in wastewaters or at sites of environmental spills, *Paenibacillus* can produce various enzymes that metabolize aliphatic and aromatic organic pollutants, including oxygenases, dehydrogenases, and ligninolytic enzymes [121, 122]. The textiles industry produces multiple chemicals that can pollute natural waters and soils, most notably dyes that are released due to inadequate wastewater treatment [123]. Strains of *Paenibacillus*, either on their own or in concert with other bacteria, are able to degrade many textile dyes [124–126] and polyvinyl alcohol (PVA), which is used as a coating for textile and paper fibers [127]. Other hazardous effluents produced by pulp and paper mills can be decontaminated by *Paenibacillus* [128].

*Paenibacillus* strains can also degrade pollutants derived from extracting, refining, and transporting petroleum and coal tar, including crude oil [129], diesel fuel [130], bitumen [131], disulfide oils [132]; and the polycyclic aromatic hydrocarbons (PAHs) naphthalene [133], phenanthrene [134], and pyrene [135]. Strains can also degrade the chemical gasoline additives ethyl tert-butyl ether [136] and benzene [134], the latter of which is also used in the production of chemicals and plastics, including nylon.

## Pathogenicity

Some *Paenibacillus* species are known to infect various organisms, including honeybees and the parasite vector *Biomphalaria glabrata*, and occasionally present as opportunistic infections in humans.

### Honeybee disease

The most studied disease associated with *Paenibacillus* is American Foulbrood (AFB), caused by *P. larvae*. AFB afflicts honeybee (*Apis* species) colonies globally, and is the most destructive brood disease [137].

Although tylosin, lincomycin, and oxytetracycline [138] are effective antibiotics against *P. larvae*, antibiotics are poorly metabolized by honeybees, and their residues or those of their metabolites can be stable in honey for over a year. Elimination of the residues can only occur when the honeybees consume all of it or when the beekeeper removes the contaminated food [139]. Residual antibiotics or their metabolites cause issues when the honey is meant for human consumption, as they can trigger allergic reactions, harm healthy microbiota, and confer resistance to pathogenic bacterial strains. Additionally, *P. larvae* resistance to antibiotics such as oxytetracycline has become increasingly more common in recent years [140]. The current most typical solution to deal with an AFB affliction is to burn the entire hive.

AFB is caused by four strains of *P. larvae*, named ERIC I-IV based on the identities of their enterobacterial repetitive intergenic consensus (ERIC) sequences. ERIC I and II are the types typically isolated from afflicted hives, as ERIC III and IV are less virulent [38, 140–145]. While ERIC II is the most virulent strain, ERIC I is more prevalent globally, likely because it can infect all honeybee subspecies, whereas ERIC II is restricted to certain subspecies [137].

As the most virulent strain, *P. larvae* ERIC II has a larval LT_100_ (time it takes the entire colony’s larvae to die) of just 7 days [145]. This strain produces a unique functional S-layer protein which facilitates attachment of the bacteria to the peritrophic matrix that lines the honeybee’s midgut epithelium [146]. By contrast, *P. larvae* ERIC I has a larval LT_100_ of 12 days [145] and produces a toxin known as the C3larvin toxin which may contribute to its pathogenicity. Although its target substrate has not been determined, this toxin acts as an ADP-ribosyltransferase and is lethal when expressed in yeast in vitro [147].

Both ERIC I and ERIC II have an invasive spore stage and a non-invasive vegetative stage. Initial ingestion of spore-contaminated food by the larvae marks the beginning of the non-invasive phase. The spores travel to the midgut lumen and germinate, after which the bacteria proliferate rapidly. Honeybee larvae are most susceptible to infection within the first 36 h after hatching, and only a few spores are needed to initiate infection. After this window, the peritrophic matrix is too thick for the bacteria to penetrate and colonization is never achieved. Both strains use chitinases to degrade the peritrophic matrix for both nourishment and access to the midgut epithelium [146].

The S-layer protein expressed by ERIC II facilitates initial association of the bacteria to the peritrophic matrix, and ERIC I may have a similar protein. If the bacteria are successful in degrading the peritrophic matrix, they eventually penetrate into the midgut epithelium and the haemocoel using chitinases and proteases, marking the invasive phase. The host larvae dies soon after from bacteremia [145]. Adult honeybees are not susceptible to infection, and so in the process of cleaning contaminated cells they tend to transfer spores all over the hive. In this way the infection spreads horizontally. The infection can also spread vertically when a contaminated mother colony infects its daughter swarm upon establishment of a new colony. The horizontal mode of transmission is much more virulent and can occur both within and between hives [148]. Further attributing to the pathogenicity of *P. larvae* are the paenilimicins and paenilarvins that it produces. Paenilimicins are antimicrobials that fight ecological niche competitors and are not directly involved in killing the bee larvae, while paenilarvins are antifungal compounds which also negatively affect bee larvae [142, 143]. As such, paenilicmins and paenilarvins promote the survival and colonization of *P. larvae* within honeybee larvae, while paenilarvins have the added disadvantage of directly impacting the health of the bee. ERIC II produces four paenilimicins: A1, A2, B1, and B2. In addition, paenilarvins A and B are produced as a secondary metabolite during the infection of *P. larvae* [143]. Other nonribosomal peptides and peptide-polyketide hybrids are *P. larvae* secondary metabolites which are expected to have additional roles in the bacteria’s pathogenicity [149].

Several naturally isolated substances and compounds have shown potential in *P. larvae* inhibition. Both monofloral and polyfloral honey demonstrate in vitro activity against *P. larvae* strains, and this is likely due in part to the high concentration of sugar causing extreme osmotic stress for the bacteria. In addition, nectar contains several antimicrobial secondary plant metabolites whose presence can be detected in processed honey. Because these secondary metabolites are species-specific in their antagonism, polyfloral honey tends to confer more resistance than monofloral honey, as it contains the metabolites from several plant nectars [140]. Propolis, a substance derived from plant resins, is used by bees in the construction of their hives and also shows inhibitory activity against *P. larvae* [150, 151]. It was also discovered that multiple Hypericum extracts have the potential to inhibit *P. larvae* in vitro, including hyperforin, uliginosin A and B, 7-epiclusianone, albasidin AA, and drummondin E [144]. Other compounds demonstrating potential to control *P. larvae* infections include *Azadirachta indica, Vitex trifolia*, *Calendula officinalis*, and *Nasturtium officinale* extracts [152, 153], a collection of essential oils [154], and frozen as well as freeze-dried sunflower bee pollen [155]. Certain other bacterial species demonstrate antagonistic activity to *P. larvae*, including *Bacillus polymachus* which shows potential as a biocontrol agent [156]. Multiple *Enterococcus* species have demonstrated antagonistic activity to *P. larvae*, and this is believed to be due to the bacteriocin genes found in their genomes [157]. A novel *P. larvae* phage phiIBB-Pl23 produces an endolysin which effectively kills *P. larvae* while at the same time remains nontoxic to bees [158].

Different species of honeybees glycosylate their royal jelly proteins in distinct patterns, which seems to have an influence on their vulnerability to *P. larvae* infections. *Apis mellifera* lingustica’s royal jelly proteins display higher antihypertension activity than those produced by *Apis cerana* cerana and is active at lower levels. Interestingly, this difference in activity grants *Apis mellifera* lingustica optimized molecular functioning and enhanced immune activity, and so the strain is more resistant to *P. larvae* than is *Apis cerana* cerana in that larval exposure to the spores results in infection less frequently [159]. Although the strain tested was ERIC I, this may help to explain the specificity observed in *P. larvae* ERIC II infections. These findings have implications when exploring potential techniques to control AFB, as well as in discovering novel techniques for controlling human hypertensive disease.

Another species of bacteria believed to put the honeybee at risk is *P. apiarius* [160]. Although not the primary cause of disease, *P. alvei* acts as a secondary infector in cases of European Foulbrood, along with other species of pathogenic bacteria including *Enterococcus faecalis*, *Brevibacillus laterosporus*, *Bacillus pumilus*, *Achromobacter euridice* [140], and *P. dendritiformis* [161].

### Snail disease

*P. glabratella* was originally isolated from visible white nodules on snails in a population with high mortality. This species causes significant mortality in infected *Biomphalaria glabrata* snail populations, and is highly contagious among members of the population. Moreover, infected snails produce infected eggs causing decreased levels of hatching. These findings hold promise for *P. glabratella* as a potential biocontrol agent against the tropical parasitic disease Schistosomiasis which commonly uses these snails as vectors [162]. This has implications in developing countries where parasites are a significant cause of death. Schistosomiasis ranks second globally among parasitic diseases of public health and socio-economic importance and is endemic in Africa [163].

### Opportunistic infections of humans

Several *Paenibacillus* species have been isolated from humans globally (Additional file 1). Although the majority of these colonisations are not harmful to their host, some have demonstrated pathogenicity to humans. In almost every case, *Paenibacillus* infections are opportunistic and tend to infect immunocompromised people. Diseases or syndromes associated with *Paenibacillus* infection include chronic kidney disease [164], sickle cell disease [165], premature birth [166], Whipple’s disease [167], hydrocephalus [168], skin cancer, chronic interstitial nephropathy, and acute lymphoblastic leukemia [169]. In many of these cases it is unclear whether the relationship between the infection and the disease was correlated or causal, but it is likely that *Paenibacillus* was simply occupying suitable niches opportunistically. Many human-recovered *Paenibacillus* isolates come from elderly patients whose immune systems are generally weak.

A significant risk factor to *Paenibacillus* infection is the use of intravenous drugs. Intravenous drug use grants bacteria and other contaminants entry into the blood stream which would normally be unexposed to these pathogens. In one case, *P. amylolyticus* coinfection with *Lysinibacillus fusiformis* caused bacteremia leading to sepsis in an immunocompromised patient known to use intravenous heroin [170]. Additionally, several cases of *P. larvae* bacteremia have been caused by the use of intravenous methadone prepared with honey from hives infected with *P. larvae.* Some pharmacies are known to prepare methadone using a viscous substance such as honey to prevent patients from misusing it through injection. If residual *P. larvae* spores in the honey happen to be injected through intentional misuse, they can germinate under proper conditions and cause infection [171].

*Paenibacillus* isolated from humans have demonstrated a variety of strain-dependent drug resistances including resistances to norfloxacin, clindamycin, ampicillin, and ticarcillinclavulanic acid. Successful drug treatments have included cefotaxime, ceftrixone, and a treatment involving amikacin and zosyn followed by po Levofloxacin [164, 165, 172].

## Dairy spoilage

Another well-known negative aspect of *Paenibacillus* is its role in the spoilage of milk and other dairy products. *Paenibacillus* is among the most important bacterial genera that produce spoilage enzymes in the dairy industry, along with *Bacillus* and *Viridibacillus*. Endospores of these genera are able to survive extreme conditions including high heat, pressure, biocides, and UV irradiation, allowing them to withstand pasteurization and persist in industrial equipment. Small numbers of *Paenibacillus* spores can therefore be found in both raw and pasteurized milk [173].

Many *Paenibacillus* strains also grow well at refrigeration temperature. *Paenibacillus* represents more than 95% of bacteria in raw milk after 10 days of refrigerated shelf life [173], while spoilage of pasteurized milk due to *Paenibacillus* is delayed by the germination process of spores and usually occurs after 17–21 days [174]. Strains that are able to grow at low temperature (6 °C) share numerous genetic features including genes that encode peptidases with cold-adapted features and cold-adaptation related proteins [175].

While *Paenibacillus* enzymes can be beneficial to other industries (see “Process manufacturing” section), their proteases, lipases, and phospholipases negatively impact the texture of dairy products, such as curdling caused by proteases, as well as the flavour [173]. However, not all dairy-isolated strains produce these activities [174]. *Paenibacillus* species isolated from dairy products include *P. amylolyticus*, *P. lactis*, *P. lentimorbus*, *P. lucanolyticus*, *P. odorifer*, *P. peoriae*, and *P. stellifer* [173, 174, 176].

## Conclusions

*Paenibacillus* was separated from *Bacillus* in 1993, but is a paraphyletic genus that is likely to undergo further subdivision in the future. This diverse genus has relevance in many areas, spurring numerous genome sequencing projects and patents. Many species are well-known plant-growth promoters, with various strains capable of promoting plant nutrient uptake, controlling phytopathogens, and producing phytohormones. The usefulness of inoculating *Paenibacillus* in the field can be restricted by various environmental conditions, but further research into the establishment and performance of these species within complex soil ecosystems may allow for their widespread use as biofertilizer. In addition to agricultural applications, *Paenibacillus* produces a diversity of antimicrobials, enzymes, and exopolysaccharides with relevance in medicine, process manufacturing, and bioremediation, some of which have already been commercialized. However, the genus is not purely beneficial, with some species causing food spoilage, honeybee disease, or opportunistic infections in humans. Still, the future can expect more discoveries and optimizations that will allow *Paenibacillus* to contribute positively to health and sustainable processes.

## Additional files

**Additional file 1.** All discovered *Paenibacillus* species along with their countries, environments, and years of isolation.

**Additional file 2.** List of *Paenibacillus* genome sequencing projects and their progress.

**Additional file 3.** Secreted compounds and significant enzymes produced by members of the genus *Paenibacillus.*

## Abbreviations

ABC: ATP-binding cassette
AFB: American Foulbrood
Anf: iron-dependent nitrogenase
Cry: crystal protein
DHB: dihydroxybenzoate
EPS: exo-polysaccharide
ERIC: enterobacterial repetitive intergenic consensus
IAA: indole-3-acetic acid
IpdC: indolepyruvate decarboxylase
ISR: induced systemic resistance
LT_100_: time required to kill 100% of subjects
Nif: molybdenum-dependent nitrogenase
NRSP: nonribosomal peptide synthetase
PAH: polycyclic aromatic hydrocarbon
PVA: polyvinyl alcohol
Vnf: vanadium- and iron-dependent nitrogenase
VOC: volatile organic compound

## References

[1] Zeigler DR. The family Paenibacillacea. In: Strain catalog and reference. Columbus: Bacillus Genetic Stock Center; 2013. p. 1–32.

[2] Priest FG, Goodfellow M, Todd C (1988). A numerical classification of the genus *Bacillus*. J Gen Microbiol.

[3] Ash C, Farrow J, Wallbanks S, Collins M (1991). Phylogenetic heterogeneity of the genus *Bacillus* revealed by comparative analysis of small-subunit-ribosomal RNA sequences. Lett Appl Microbiol.

[4] Ash C, Priest F, Collins MD (1993). Molecular identification of rRNA group 3 bacilli (Ash, Farrow, Wallbanks and Collins) using a PCR probe test. Antonie Van Leeuwenhoek.

[5] Trüper HG (2005). The type species of the genus *Paenibacillus* Ash et al. 1994 is *Paenibacillus polymyxa*. Opinion 77. Int J Syst Evol Microbiol.

[6] Heyndrickx M, Vandemeulebroecke K, Scheldeman P, Hoste B, Kersters K, De Vos P (1995). *Paenibacillus* (Formerly *Bacillus*) *gordonae* (Pichinoty et. al. 1986) Ash et al. 1994 is a later subjective synonym of *Paenibacillus* (Formerly *Bacillus*) *validus* (Nakamura 1984) Ash et al. 1994: emended description of *P. validus*. Int J Syst Bacteriol.

[7] Heyndrickx M, Vandemeulebroecke K, Hoste B, Janssen P, Kersters K, De Vos P (1996). Reclassification of *Paenibacillus* (formerly *Bacillus*) *pulvifaciens* (Nakamura 1984) Ash et al. 1994, a later subjective synonym of *Paenibacillus* (formerly *Bacillus*) *larvae* (White 1906) Ash et al. 1994, as a subspecies of *P. larvae*, with emended descriptions of *P. larvae* as *P. larvae* subsp. larvae and *P. larvae* subsp. *pulvifaciens*. Int J Syst Bacteriol.

[8] Shida O, Takagi H, Kadowaki K, Nakamura I, Komagata K (1997). Transfer of *Bacillus alginolyticus*, *Bacillus chondroitinus, Bacillus curdlanolyticus, Bacillus glucanolyticus, Bacillus kobensis*, and *Bacillus thiaminolyticus* to the genus *Paenibacillus* and emended description of the genus *Paenibacillus*. Int J Syst Bacteriol.

[9] Collins MD, Lawson PA, Willems A, Cordoba JJ, Fernandez-Garayzabal J, Garcia P (1994). The phylogeny of the genus *Clostridium*: proposal of five new genera and eleven new species combinations. Int J Syst Bacteriol.

[10] Dsouza M, Taylor MW, Turner SJ, Aislabie J (2014). Genome-based comparative analyses of Antarctic and temperate species of *Paenibacillus*. PLoS ONE.

[11] Xiao B, Sun YF, Lian B, Chen TM (2016). Complete genome sequence and comparative genome analysis of the *Paenibacillus mucilaginosus* K02. Microb Pathog.

[12] Yao R, Wang R, Wang D, Su J, Zheng SX, Wang GJ (2014). *Paenibacillus selenitireducens* sp. nov., a selenite-reducing bacterium isolated from a selenium mineral soil. Int J Syst Evol Microbiol.

[13] Sheela T, Usharani P (2013). Influence of plant growth promoting rhizobacteria (PGPR) on the growth of maize (*Zea mays* L.). Gold Res Thoughts.

[14] Han Z, Zhang Z, Dong Y, Yang M (2014). Effects of endophytic bacteria P22 and S16 in *Populus* on the rooting and growth of the relative species plants. J Northeast For Univ.

[15] Fürnkranz M, Adam E, Müller H, Grube M, Huss H, Winkler J (2012). Promotion of growth, health and stress tolerance of Styrian oil pumpkins by bacterial endophytes. Eur J Plant Pathol.

[16] de Souza R, Meyer J, Schoenfeld R, da Costa PB, Passaglia LMP (2014). Characterization of plant growth-promoting bacteria associated with rice cropped in iron-stressed soils. Ann Microbiol.

[17] Ker K, Seguin P, Driscoll BT, Fyles JW, Smith DL (2014). Switchgrass establishment and seeding year production can be improved by inoculation with rhizosphere endophytes. Biomass Bioenergy.

[18] Weselowski B, Nathoo N, Eastman AW, MacDonald J, Yuan ZC (2016). Isolation, identification and characterization of *Paenibacillus polymyxa* CR1 with potentials for biopesticide, biofertilization, biomass degradation and biofuel production. BMC Microbiol.

[19] Sharma SB, Sayyed RZ, Trivedi MH, Gobi TA (2013). Phosphate solubilizing microbes: sustainable approach for managing phosphorus deficiency in agricultural soils. SpringerPlus.

[20] Duca D, Lorv J, Patten CL, Rose D, Glick BR (2014). Indole-3-acetic acid in plant-microbe interactions. Antonie Van Leeuwenhoek.

[21] Galloway JN, Cowling EB (2002). Reactive nitrogen and the world: 200 years of change. Ambio.

[22] Näsholm T, Kielland K, Ganeteg U (2009). Uptake of organic nitrogen by plants. New Phytol.

[23] Boyd ES, Peters JW (2013). New insights into the evolutionary history of biological nitrogen fixation. Front Microbiol.

[24] Li XX, Liu Q, Liu XM, Shi HW, Chen SF (2016). Using synthetic biology to increase nitrogenase activity. Microb Cell Fact.

[25] Xie JB, Du Z, Bai L, Tian C, Zhang Y, Xie JY (2014). Comparative genomic analysis of N2-fixing and non-N2-fixing *Paenibacillus* spp.: organization, evolution and expression of the nitrogen fixation genes. PLoS Genet.

[26] Eastman AW, Heinrichs DE, Yuan ZC (2014). Comparative and genetic analysis of the four sequenced *Paenibacillus polymyxa* genomes reveals a diverse metabolism and conservation of genes relevant to plant-growth promotion and competitiveness. BMC Genom.

[27] Xie J, Shi H, Du Z, Wang T, Liu X, Chen S (2016). Comparative genomic and functional analysis reveal conservation of plant growth promoting traits in *Paenibacillus polymyxa* and its closely related species. Sci Rep.

[28] Fernandes GDC, Trarbach LJ, De Campos SB, Beneduzi A, Passaglia LMP (2014). Alternative nitrogenase and pseudogenes: unique features of the *Paenibacillus riograndensis* nitrogen fixation system. Res Microbiol.

[29] Wang LY, Li J, Li QX, Chen SF (2013). *Paenibacillus beijingensis* sp. nov., a nitrogen-fixing species isolated from wheat rhizosphere soil. Antonie Van Leeuwenhoek.

[30] Das SN, Dutta S, Kondreddy A, Chilukoti N, Pullabhotla SVSRN, Vadlamudi S (2010). Plant growth-promoting chitinolytic *Paenibacillus elgii* responds positively to tobacco root exudates. J Plant Growth Regul.

[31] Marra LM, Sousa Soares CRF, de Oliveira SM, Ferreira PAA, Soares BL, de Carvalho RF (2012). Biological nitrogen fixation and phosphate solubilization by bacteria isolated from tropical soils. Plant Soil.

[32] Wang Y, Shi Y, Li B, Shan C, Ibrahim M, Jabeen A (2012). Phosphate solubilization of *Paenibacillus polymyxa* and *Paenibacillus macerans* from mycorrhizal and non-mycorrhizal cucumber plants. Afr J Micro Res.

[33] Hu X, Chen J, Guo J (2006). Two phosphate- and potassium-solubilizing bacteria isolated from Tianmu Mountain, Zhejiang, China. World J Microbiol Biotechnol.

[34] Pandya M, Rajput M, Rajkumar S (2015). Exploring plant growth promoting potential of non rhizobial root nodules endophytes of *Vigna radiata*. Microbiology.

[35] Raza W, Shen Q (2010). Growth, Fe3 + reductase activity, and siderophore production by *Paenibacillus polymyxa* SQR-21 under differential iron conditions. Curr Microbiol.

[36] Hayat R, Ahmed I, Sheirdil RA, Ahmad MSA, Aksoy A (2012). An overview of plant growth promoting rhizobacteria (PGPR) for sustainable agriculture. Crop production for agricultural improvement.

[37] Wen Y, Wu X, Teng Y, Qian C, Zhan Z, Zhao Y (2011). Identification and analysis of the gene cluster involved in biosynthesis of paenibactin, a catecholate siderophore produced by *Paenibacillus elgii* B69. Environ Microbiol.

[38] Hertlein G, Müller S, Garcia-Gonzalez E, Poppinga L, Süssmuth RD, Genersch E (2014). Production of the catechol type siderophore bacillibactin by the honey bee pathogen *Paenibacillus larvae*. PLoS ONE.

[39] Zhou C, Guo J, Zhu L, Xiao X, Xie Y, Zhu J (2016). *Paenibacillus polymyxa* BFKC01 enhances plant iron absorption via improved root systems and activated iron acquisition mechanisms. Plant Physiol Biochem.

[40] Delker C, Raschke A, Quint M (2008). Auxin dynamics: the dazzling complexity of a small molecule’s message. Planta.

[41] Patten CL, Blakney AJC, Coulson TJD (2013). Activity, distribution and function of indole-3-acetic acid biosynthetic pathways in bacteria. Crit Rev Microbiol.

[42] Pichard B, Thouvenot D (1999). Effect of *Bacillus polymyxa* seed treatments on control of black-rot and damping-off of cauliflower. Seed Sci Technol.

[43] Wakelin SA, Walter M, Jaspers M, Stewart A (2002). Biological control of *Aphanomyces euteiches* root-rot of pea with spore-forming bacteria. Australas Plant Pathol.

[44] Jeon YH, Chang SP, Hwang I, Kim YH (2003). Involvement of growth-promoting rhizobacterium *Paenibacillus polymyxa* in root rot of stored Korean ginseng. J Microbiol Biotechnol.

[45] Yang J, Kharbanda PD, Mirza M (2004). Evaluation of *Paenibacillus polymyxa* PKB1 for biocontrol of pythium disease of cucumber in a hydroponic system. Acta Hortic.

[46] Akhtar MS, Siddiqui ZA (2007). Biocontrol of a chickpea root-rot disease complex with *Glomus intraradices, Pseudomonas putida* and *Paenibacillus polymyxa*. Australas Plant Pathol.

[47] Haggag WM (2007). Colonization of exopolysaccharide-producing *Paenibacillus polymyxa* on peanut roots for enhancing resistance against crown rot disease. Afr J Biotechnol.

[48] Zhou K, Yamagishi M, Osaki M (2008). *Paenibacillus* BRF-1 has biocontrol ability against *Phialophora gregata* disease and promotes soybean growth. Soil Sci Plant Nutr.

[49] Phi QT, Park YM, Seul KJ, Ryu CM, Park SH, Kim JG (2010). Assessment of root-associated *Paenibacillus polymyxa* groups on growth promotion and induced systemic resistance in pepper. J Microbiol Biotechnol.

[50] Antonopoulos DF, Tjamos SE, Antoniou PP, Rafeletos P, Tjamos EC (2008). Effect of *Paenibacillus alvei*, strain K165, on the germination of *Verticillium dahliae* microsclerotia in planta. Biol Control.

[51] Von Der Weid I, Artursson V, Seldin L, Jansson JK (2005). Antifungal and root surface colonization properties of GFP-tagged *Paenibacillus brasilensis* PB177. World J Microbiol Biotechnol.

[52] Lapidot D, Dror R, Vered E, Mishli O, Levy D, Helman Y (2014). Disease protection and growth promotion of potatoes (*Solanum tuberosum* L.) by *Paenibacillus dendritiformis*. Plant Pathol.

[53] Naing KW, Anees M, Kim SJ, Nam Y, Kim YC, Kim KY (2014). Characterization of antifungal activity of *Paenibacillus ehimensis* KWN38 against soilborne phytopathogenic fungi belonging to various taxonomic groups. Ann Microbiol.

[54] Lo Presti L, Lanver D, Schweizer G, Tanaka S, Liang L, Tollot M (2015). Fungal effectors and plant susceptibility. Annu Rev Plant Biol.

[55] Martin NI, Hu H, Moake MM, Churey JJ, Whittal R, Worobo RW (2003). Isolation, structural characterization, and properties of mattacin (Polymyxin M), a cyclic peptide antibiotic produced by *Paenibacillus kobensis* M. J Biol Chem.

[56] Bae YS, Park K, Kim CH (2004). *Bacillus* spp. as biocontrol agents of root rot and phytophthora blight on ginseng. Plant Pathol J.

[57] Gulati MK, Koch E, Sikora RA, Zeller W (2001). Biological control of *Phytophthora* diseases on strawberry with rhizobacteria. Bull OILB/SROP.

[58] Jung TK, Kim JH, Song HG (2012). Antifungal activity and plant growth promotion by rhizobacteria inhibiting growth of plant pathogenic fungi. Kor J Microbiol.

[59] Huang E, Yousef AE (2014). The lipopeptide antibiotic paenibacterin binds to the bacterial outer membrane and exerts bactericidal activity through cytoplasmic membrane damage. Appl Environ Microbiol.

[60] Singh AK, Ghodke I, Chhatpar HS (2009). Pesticide tolerance of *Paenibacillus* sp. D1 and its chitinase. J Environ Manage.

[61] Raza W, Yuan J, Wu YC, Rajer FU, Huang Q, Qirong S (2015). Biocontrol traits of two *Paenibacillus polymyxa* strains SQR-21 and WR-2 in response to fusaric acid, a phytotoxin produced by *Fusarium* species. Plant Pathol.

[62] Montesinos E (2003). Development, registration and commercialization of microbial pesticides for plant protection. Int Microbiol.

[63] Enright MR, Griffin CT (2005). Effects of *Paenibacillus nematophilus* on the entomopathogenic nematode *Heterorhabditis megidis*. J Invertebr Pathol.

[64] Pieterse CMJ, Zamioudis C, Berendsen RL, Weller DM, Van Wees SCM, Bakker PAHM (2014). Induced systemic resistance by beneficial microbes. Annu Rev Phytopathol.

[65] Lee B, Farag MA, Park HB, Kloepper JW, Lee SH, Ryu CM (2012). Induced resistance by a long-chain bacterial volatile: elicitation of plant systemic defense by a C13 volatile produced by *Paenibacillus polymyxa*. PLoS ONE.

[66] Tjamos SE, Flemetakis E, Paplomatas EJ, Katinakis P (2005). Induction of resistance to *Verticillium dahliae* in *Arabidopsis thaliana* by the biocontrol agent K-165 and pathogenesis-related proteins gene expression. Mol Plant-Microbe Interact.

[67] Sang MK, Kim EN, Han GD, Kwack MS, Jeun YC, Kim KD (2014). Priming-mediated systemic resistance in cucumber induced by *Pseudomonas azotoformans* GC-B19 and *Paenibacillus elgii* MM-B22 against *Colletotrichum orbiculare*. Phytopathology.

[68] Gkizi D, Lehmann S, L’Haridon F, Serrano M, Paplomatas EJ, Métraux JP (2016). The innate immune signaling system as a regulator of disease resistance and induced systemic resistance activity against *Verticillium dahliae*. Mol Plant-Microbe Interact.

[69] Khan Z, Son S, Akhtar J, Gautam N, Kim Y (2012). Plant growth-promoting rhizobacterium (*Paenibacillus polymyxa*) induced systemic resistance in tomato (*Lycopersicon esculentum*) against root-knot nematode (*Meloidogyne incognita*). Indian J Agric Sci.

[70] Kumar S, Chauhan PS, Agrawal L, Raj R, Srivastava A, Gupta S (2016). *Paenibacillus lentimorbus* inoculation enhances tobacco growth and extenuates the virulence of cucumber mosaic virus. PLoS ONE.

[71] Farag MA, Zhang H, Ryu CM (2013). Dynamic chemical communication between plants and bacteria through airborne signals: induced resistance by bacterial volatiles. J Chem Ecol.

[72] Xin XF, He SY (2013). *Pseudomonas syringae* pv. tomato DC3000: a model pathogen for probing disease susceptibility and hormone signaling in plants. Annu Rev Phytopathol.

[73] Sharma A, Thakur DR, Kanwar S, Chandla VK (2013). Diversity of entomopathogenic bacteria associated with the white grub, *Brahmina coriacea*. J Pest Sci.

[74] Neung S, Nguyen XH, Naing KW, Lee YS, Kim KY (2014). Insecticidal potential of *Paenibacillus elgii* HOA73 and its combination with organic sulfur pesticide on diamondback moth, *Plutella xylostella*. J Kor Soc Appl Biol Chem.

[75] Klein MG (1988). Pest management of soil-inhabiting insects with microorganisms. Agric Ecosyst Environ.

[76] Singh AK, Singh A, Joshi P (2016). Combined application of chitinolytic bacterium *Paenibacillus* sp. D1 with low doses of chemical pesticides for better control of *Helicoverpa armigera*. Int J Pest Manage.

[77] Bravo A, Gill SS, Soberón M (2007). Mode of action of *Bacillus thuringiensis* Cry and Cyt toxins and their potential for insect control. Toxicon.

[78] Yokoyama T, Tanaka M, Hasegawa M (2004). Novel cry gene from *Paenibacillus lentimorbus* strain Semadara inhibits ingestion and promotes insecticidal activity in *Anomala cuprea* larvae. J Invertebr Pathol.

[79] Zhang J, Hodgman TC, Krieger L, Schnetter W, Schairer HU (1997). Cloning and analysis of the first cry gene from *Bacillus popilliae*. J Bacteriol.

[80] Gorashi N, Tripathi M, Kalia V, Gujar G (2014). Identification and characterization of the Sudanese *Bacillus thuringiensis* and related bacterial strains for their efficacy against *Helicoverpa armigera* and *Tribolium castaneum*. Indian J Exp Biol.

[81] Hussie AI, Nahed Ibrahim A, Hatem AE-S, Aldebis HK, Vargas-Osuna E (2011). Actividad insecticida y fijadora de nitrógeno de la bacteria transformada *Paenibacillus polymyxa* que expresa Cry1C. Rev Col Entomol.

[82] Ibrahim NAGA, Hussien AI, Hatem AES, Aldebis HK, Vargas-Osuna E (2014). Persistence of the transformed *Paenibacillus polymyxa* expressing CRY1C in the plant leaves and its effect on chlorophyll and carotenoid. Life Sci J.

[83] Kim B, Song GC, Ryu CM (2016). Root exudation by aphid leaf infestation recruits root-associated *Paenibacillus* spp. to lead plant insect susceptibility. J Microbiol Biotechnol.

[84] Lorentz RH, Ártico S, Da Silveira AB, Einsfeld A, Corção G (2006). Evaluation of antimicrobial activity in *Paenibacillus* spp. strains isolated from natural environment. Lett Appl Microbiol.

[85] Kim SG, Khan Z, Jeon YH, Kim YH (2009). Inhibitory effect of *Paenibacillus polymyxa* GBR-462 on *Phytophthora capsici* causing phytophthora blight in chili pepper. J Phytopathol.

[86] Allard S, Enurah A, Strain E, Millner P, Rideout SL, Brown EW (2014). In situ evaluation of *Paenibacillus alvei* in reducing carriage of *Salmonella enterica* serovar newport on whole tomato plants. Appl Environ Microbiol.

[87] Hong T-Y, Meng M (2003). Biochemical characterization and antifungal activity of an endo-1, 3-β-glucanase of *Paenibacillus* sp. isolated from garden soil. Appl Microbiol Biotechnol.

[88] Aktuganov G, Melentjev A, Galimzianova N, Khalikova E, Korpela T, Susi P (2008). Wide-range antifungal antagonism of *Paenibacillus ehimensis* IB-X-b and its dependence on chitinase and β-1,3-glucanase production. Can J Microbiol.

[89] Li J, Liu W, Luo L, Dong D, Liu T, Zhang T (2015). Expression of *Paenibacillus polymyxa* β-1, 3-1, 4-glucanase in *Streptomyces lydicus* A01 improves its biocontrol effect against *Botrytis cinerea*. Biol Control.

[90] Seo DJ, Lee YS, Kim KY, Jung WJ (2016). Antifungal activity of chitinase obtained from *Paenibacillus ehimensis* MA2012 against conidial of *Collectotrichum gloeosporioides* in vitro. Microb Pathog.

[91] Singh AK, Chhatpar HS (2011). Purification and characterization of chitinase from *Paenibacillus* sp. D1. Appl Biochem Biotechnol.

[92] Morath SU, Hung R, Bennett JW (2012). Fungal volatile organic compounds: a review with emphasis on their biotechnological potential. Fungal Biol Rev.

[93] Garbeva P, Hordijk C, Gerards S, De Boer W (2014). Volatile-mediated interactions between phylogenetically different soil bacteria. Front Microbiol.

[94] Raza W, Yuan J, Ling N, Huang Q, Shen Q (2015). Production of volatile organic compounds by an antagonistic strain *Paenibacillus polymyxa* WR-2 in the presence of root exudates and organic fertilizer and their antifungal activity against *Fusarium oxysporum* f. sp. niveum. Biol Control.

[95] Lee YS, Nguyen XH, Cho JY, Moon JH, Kim KY. Isolation and antifungal activity of methyl 2,3-dihydroxybenzoate from *Paenibacillus elgii* HOA73. Microb Pathog 2016. 10.1016/j.micpath.2016.01.007 26796297

[96] Huang E, Yousef AE (2015). Biosynthesis of paenibacillin, a lantibiotic with N-terminal acetylation, by *Paenibacillus polymyxa*. Microbiol Res.

[97] Barbosa J, Caetano T, Mendo S (2015). Class I and class II lanthipeptides produced by *Bacillus* spp.. J Nat Prod.

[98] Lohans CT, Huang Z, Van Belkum MJ, Giroud M, Sit CS, Steels EM (2012). Structural characterization of the highly cyclized lantibiotic paenicidin A via a partial desulfurization/reduction strategy. J Am Chem Soc.

[99] Baindara P, Chaudhry V, Mittal G, Liao LM, Matos CO, Khatri N (2016). Characterization of the antimicrobial peptide penisin, a class Ia novel lantibiotic from *Paenibacillus* sp. strain A3. Antimicrob Agents Chemother.

[100] He Z, Kisla D, Zhang L, Yuan C, Green-Church KB, Yousef AE (2007). Isolation and identification of a *Paenibacillus polymyxa* strain that coproduces a novel lantibiotic and polymyxin. Appl Environ Microbiol.

[101] Abriouel H, Franz CMAP, Omar NB, Galvez A (2011). Diversity and applications of *Bacillus* bacteriocins. FEMS Microbiol Rev.

[102] Cochrane SA, Vederas JC (2014). Lipopeptides from *Bacillus* and *Paenibacillus* spp.: a gold mine of antibiotic candidates. Med Res Rev.

[103] Tambadou F, Caradec T, Gagez A-L, Bonnet A, Sopéna V, Bridiau N (2015). Characterization of the colistin (polymyxin E1 and E2) biosynthetic gene cluster. Arch Microbiol.

[104] Mousa WK, Raizada MN (2015). Biodiversity of genes encoding anti-microbial traits within plant associated microbes. Front Plant Sci.

[105] Shoji J, Kato T, Hinoo I (1977). The structure of polymyxin S1. Studies on antibiotics from the genus *Bacillus*. XXI. J Antibiot.

[106] Shoji J, Kato T, Hinoo H (1977). The structure of polymyxin T1. Studies on antibiotics from the genus *Bacillus*. XXII. J Antibiot.

[107] Choi SK, Park SY, Kim R, Kim SB, Lee CH, Kim JF (2009). Identification of a polymyxin synthetase gene cluster of *Paenibacillus polymyxa* and heterologous expression of the gene in *Bacillus subtilis*. J Bacteriol.

[108] Velkov T, Thompson PE, Nation RL, Li J (2010). Structure-activity relationships of polymyxin antibiotics. J Med Chem.

[109] Velkov T, Roberts KD, Nation RL, Wang J, Thompson PE, Li J (2014). Teaching ‘old’ polymyxins new tricks: new-generation lipopeptides targeting gram-negative ‘superbugs’. ACS Chem Biol.

[110] Kajimura Y, Kaneda M, Fusaricidin A (1996). A new depsipeptide antibiotic produced by *Bacillus polymyxa* KT-8 taxonomy, fermentation, isolation, structure elucidation and biological activity. J Antibiot.

[111] Bionda N, Pitteloud JP, Cudic P (2013). Cyclic lipodepsipeptides: a new class of antibacterial agents in the battle against resistant bacteria. Future Med Chem.

[112] Han JW, Kim EY, Lee JM, Kim YS, Bang E, Kim BS (2012). Site-directed modification of the adenylation domain of the fusaricidin nonribosomal peptide synthetase for enhanced production of fusaricidin analogs. Biotechnol Lett.

[113] Liang TW, Wang SL (2015). Recent advances in exopolysaccharides from *Paenibacillus* spp.: production, isolation, structure, and bioactivities. Mar Drugs.

[114] Liu J, Luo J, Ye H, Zeng X (2012). Preparation, antioxidant and antitumor activities in vitro of different derivatives of levan from endophytic bacterium *Paenibacillus polymyxa* EJS-3. Food Chem Toxicol.

[115] Pleszczyńska M, Wiater A, Janczarek M, Szczodrak J (2015). (1 → 3)-α-d-Glucan hydrolases in dental biofilm prevention and control: a review. Int J Biol Macromol.

[116] Saxena N, Pore S, Arora P, Kapse N, Engineer A, Ranade DR (2015). Cultivable bacterial flora of Indian oil reservoir: isolation, identification and characterization of the biotechnological potential. Biologia.

[117] Yadav AN, Sachan SG, Verma P, Saxena AK (2016). Bioprospecting of plant growth promoting psychrotrophic bacilli from the cold desert of north western indian himalayas. Indian J Exp Biol.

[118] Mihajlovski KR, Carević MB, Dević ML, Šiler-Marinković S, Rajilić-Stojanović MD, Dimitrijević-Branković S (2015). Lignocellulosic waste material as substrate for Avicelase production by a new strain of *Paenibacillus chitinolyticus* CKS1. Int Biodeterior Biodegrad.

[119] Raddadi N, Cherif A, Daffonchio D, Fava F (2013). Halo-alkalitolerant and thermostable cellulases with improved tolerance to ionic liquids and organic solvents from *Paenibacillus tarimensis* isolated from the Chott El Fejej, Sahara desert, Tunisia. Bioresour Technol.

[120] Li O, Lu C, Liu A, Zhu L, Wang PM, Qian CD (2013). Optimization and characterization of polysaccharide-based bioflocculant produced by *Paenibacillus elgii* B69 and its application in wastewater treatment. Bioresour Technol.

[121] Abbasian F, Lockington R, Mallavarapu M, Naidu R (2015). A comprehensive review of aliphatic hydrocarbon biodegradation by bacteria. Appl Biochem Biotechnol.

[122] Haritash A, Kaushik C (2009). Biodegradation aspects of polycyclic aromatic hydrocarbons (PAHs): a review. J Hazard Mater.

[123] Spadaro JT, Isabelle L, Renganathan V (1994). Hydroxyl radical mediated degradation of azo dyes: evidence for benzene generation. Environ Sci Technol.

[124] Ramya M, Anusha B, Kalavathy S (2008). Decolorization and biodegradation of Indigo carmine by a textile soil isolate *Paenibacillus larvae*. Biodegradation.

[125] Moosvi S, Kher X, Madamwar D (2007). Isolation, characterization and decolorization of textile dyes by a mixed bacterial consortium JW-2. Dyes Pigment.

[126] Watanapokasin RY, Boonyakamol A, Sukseree S, Krajarng A, Sophonnithiprasert T, Kanso S (2009). Hydrogen production and anaerobic decolorization of wastewater containing reactive blue 4 by a bacterial consortium of *Salmonella subterranea* and *Paenibacillus polymyxa*. Biodegradation.

[127] Choi K, Park C, Kim S, Lyoo W, Lee SH, Lee J (2004). Polyvinyl alcohol degradation by *Microbacterium barkeri* KCCM 10507 and *Paenibacillus amylolyticus* KCCM 10508 in dyeing wastewater. J Microbiol Biotechnol.

[128] Raj A, Kumar S, Haq I, Singh SK (2014). Bioremediation and toxicity reduction in pulp and paper mill effluent by newly isolated ligninolytic *Paenibacillus* sp.. Ecol Eng.

[129] Almeida PF, Moreira RS, Almeida RCC, Guimarães AK, Carvalho AS, Quintella C (2004). Selection and application of microorganisms to improve oil recovery. Eng Life Sci.

[130] Ganesh A, Lin J (2009). Diesel degradation and biosurfactant production by gram-positive isolates. Afr J Biotechnol.

[131] Adebayo EA, Oloke JK, Aina DA (2009). Effect of culture parameters of a bacterial consortium on biodegradation of bitumen. Adv Environ Biol.

[132] Esmaeili Taheri H, Hatamipour MS, Emtiazi G, Beheshti M (2008). Bioremediation of DSO contaminated soil. Process Saf Environ Prot.

[133] Haggblom MM (2005). Microbe degrades naphthalene. Ind Bioprocess.

[134] Al-Saleh E, Obuekwe C (2014). Crude oil biodegradation activity in potable water. Int Biodeterior Biodegrad.

[135] Deora A, Giri R, Suneja S, Goyal S, Kukreja K (2012). Isolation and characterization of pyrene degrading bacteria. Pollut Res.

[136] Guisado IM, Purswani J, Gonzalez-Lopez J, Pozo C (2015). Physiological and genetic screening methods for the isolation of methyl tert-butyl ether-degrading bacteria for bioremediation purposes. Int Biodeterior Biodegrad.

[137] Morrissey BJ, Helgason T, Poppinga L, Fünfhaus A, Genersch E, Budge GE (2015). Biogeography of *Paenibacillus larvae*, the causative agent of American foulbrood, using a new multilocus sequence typing scheme. Environ Microbiol.

[138] Reynaldi FJ, Albo GN, Alippi AM (2008). Effectiveness of tilmicosin against *Paenibacillus larvae*, the causal agent of American Foulbrood disease of honeybees. Vet Microbiol.

[139] Reybroeck W, Daeseleire E, De Brabander HF, Herman L (2012). Antimicrobials in beekeeping. Vet Microbiol.

[140] Erler S, Denner A, Bobiş O, Forsgren E, Moritz RFA (2014). Diversity of honey stores and their impact on pathogenic bacteria of the honeybee, *Apis mellifera*. Ecol Evol.

[141] Garcia-Gonzalez E, Müller S, Ensle P, Süssmuth RD, Genersch E (2014). Elucidation of sevadicin, a novel non-ribosomal peptide secondary metabolite produced by the honey bee pathogenic bacterium *Paenibacillus larvae*. Environ Microbiol.

[142] Müller S, Garcia-Gonzalez E, Mainz A, Hertlein G, Heid NC, Mösker E (2014). Paenilamicin: structure and biosynthesis of a hybrid nonribosomal peptide/polyketide antibiotic from the bee pathogen *Paenibacillus larvae*. Angew Chem Int Ed.

[143] Sood S, Steinmetz H, Beims H, Mohr KI, Stadler M, Djukic M (2014). Paenilarvins: Iturin family Lipopeptides from the honey bee pathogen *Paenibacillus larvae*. ChemBioChem.

[144] Hernández-Lõpez J, Crockett S, Kunert O, Hammer E, Schuehly W, Bauer R (2014). In vitro growth inhibition by Hypericum extracts and isolated pure compounds of *Paenibacillus larvae*, a lethal disease affecting honeybees worldwide. Chem Biodivers.

[145] Djukic M, Brzuszkiewicz E, Fünfhaus A, Voss J, Gollnow K, Poppinga L (2014). How to kill the honey bee larva: genomic potential and virulence mechanisms of *Paenibacillus larvae*. PLoS ONE.

[146] Garcia-Gonzalez E, Genersch E (2013). Honey bee larval peritrophic matrix degradation during infection with *Paenibacillus larvae*, the aetiological agent of American foulbrood of honey bees, is a key step in pathogenesis. Environ Microbiol.

[147] Krska D, Ravulapalli R, Fieldhouse RJ, Lugo MR, Merrill AR (2015). C3larvin toxin, an ADP-ribosyltransferase from *Paenibacillus larvae*. J Biol Chem.

[148] Jatulan EO, Rabajante JF, Banaay CGB, Fajardo AC, Jose EC (2015). A mathematical model of intra-colony spread of American foulbrood in European honeybees (Apis mellifera L.). PLoS ONE.

[149] Müller S, Garcia-Gonzalez E, Genersch E, Süssmuth RD (2015). Involvement of secondary metabolites in the pathogenesis of the American foulbrood of honey bees caused by *Paenibacillus larvae*. Nat Prod Rep.

[150] Wilson MB, Brinkman D, Spivak M, Gardner G, Cohen JD (2015). Regional variation in composition and antimicrobial activity of US propolis against *Paenibacillus larvae* and *Ascosphaera apis*. J Invertebr Pathol.

[151] Boonsai P, Phuwapraisirisan P, Chanchao C (2014). Antibacterial activity of a cardanol from thai *Apis mellifera* propolis. Int J Med Sci.

[152] Anjum SI, Ayaz S, Shah AH, Khan S, Khan SN (2015). Controlling honeybee pathogen by using neem and Barbaka plant extracts. Biotechnol Biotechnol Equip.

[153] Piana M, De Brum TF, Boligon AA, Alves CF, De Freitas RB, Nunes LT (2015). In vitro growth-inhibitory effect of brazilian plants extracts against *Paenibacillus larvae* and toxicity in bees. An Acad Bras Cienc.

[154] Ansari MJ, Al-Ghamdi A, Usmani S, Al-Waili N, Nuru A, Sharma D (2016). In vitro evaluation of the effects of some plant essential oils on *Paenibacillus larvae*, the causative agent of American foulbrood. Biotechnol Biotechnol Equip.

[155] Fatrcová-Šramková K, Nôžková J, Máriássyová M, Kačániová M (2016). Biologically active antimicrobial and antioxidant substances in the *Helianthus annuus* L. bee pollen. J Environ Sci Health, Part B.

[156] Nguyen TM, Kim J (2015). *Bacillus polymachus* sp. nov., with a broad range of antibacterial activity, isolated from forest topsoil samples by using a modified culture method. Int J Syst Evol Microbiol.

[157] Jaouani I, Abbassi MS, Alessandria V, Bouraoui J, Ben Salem R, Kilani H (2014). High inhibition of *Paenibacillus larvae* and *Listeria monocytogenes* by enterococcus isolated from different sources in tunisia and identification of their bacteriocin genes. Lett Appl Microbiol.

[158] Oliveira A, Leite M, Kluskens LD, Santos SB, Melo LDR, Azeredo J (2015). The first *Paenibacillus larvae* bacteriophage endolysin (PlyPl23) with high potential to control American foulbrood. PLoS ONE.

[159] Feng M, Fang Y, Han B, Xu X, Fan P, Hao Y (2015). In-depth N-Glycosylation reveals species-specific modifications and functions of the royal jelly protein from western (*Apis mellifera*) and eastern honeybees (*Apis cerana*). J Proteome Res.

[160] Katznelson H (1955). *Bacillus apiarius*, n. sp., an aerobic spore-forming organism isolated from honeybee larvae. J Bacteriol.

[161] Gaggìa F, Baffoni L, Stenico V, Alberoni D, Buglione E, Lilli A (2015). Microbial investigation on honey bee larvae showing atypical symptoms of European foulbrood. Bull Insectol.

[162] Duval D, Galinier R, Mouahid G, Toulza E, Allienne JF, Portela J (2015). A novel bacterial pathogen of *Biomphalaria glabrata*: a potential weapon for schistosomiasis control?. PLoS Negl Trop Dis.

[163] Isaac O (2009). Prevalence of snail vectors of schistosomiasis and their infection rates in two localities within Ahmadu Bello University (ABU) Campus, Zaria, Kaduna State, Nigeria. J Cell Anim Biol.

[164] Padhi S, Dash M, Sahu R, Panda P (2013). Urinary tract infection due to *Paenibacillus alvei* in a chronic kidney disease: a rare case report. J Lab Phys.

[165] Reboli AC, Bryan CS, Farrar WE (1989). Bacteremia and infection of a hip prosthesis caused by *Bacillus alvei*. J Clin Microbiol.

[166] DeLeon SD, Welliver RC (2016). *Paenibacillus alvei* sepsis in a neonate. Pediatr Infect Dis J.

[167] Roux V, Fenner L, Raoult D (2008). *Paenibacillus provencensis* sp. nov., isolated from human cerebrospinal fluid, and *Paenibacillus urinalis* sp. nov., isolated from human urine. Int J Syst Evol Microbiol.

[168] Bosshard PP, Zbinden R, Altwegg M (2002). *Paenibacillus turicensis* sp. nov., a novel bacterium harbouring heterogeneities between 16S rRNA genes. Int J Syst Evol Microbiol.

[169] Roux V, Raoult D (2004). *Paenibacillus massiliensis* sp. nov. *Paenibacillus sanguinis* sp. nov. and *Paenibacillus timonensis* sp. nov., isolated from blood cultures. Int J Syst Evol Microbiol.

[170] Wenzler E, Kamboj K, Balada-Llasat JM (2015). Severe sepsis secondary to persistent *Lysinibacillus sphaericus, Lysinibacillus fusiformis* and *Paenibacillus amylolyticus* bacteremia. Int J Infect Dis.

[171] Rieg S, Bauer TM, Peyerl-Hoffmann G, Held J, Ritter W, Wagner D (2010). *Paenibacillus larvae* bacteremia in injection drug users. Emerg Infect Dis.

[172] Ouyang J, Pei Z, Lutwick L, Dalai S, Yang L, Cassai N (2008). Case report: *Paenibacillus thiaminolyticus*: a new cause of human infection, inducing bacteremia in a patient on hemodialysis. Ann Clin Lab Sci.

[173] Gopal N, Hill C, Ross PR, Beresford TP, Fenelon MA, Cotter PD (2015). The prevalence and control of *Bacillus* and related spore-forming bacteria in the dairy industry. Front Microbiol.

[174] Trmčić A, Martin NH, Boor KJ, Wiedmann M (2015). A standard bacterial isolate set for research on contemporary dairy spoilage. J Dairy Sci.

[175] Moreno Switt AI, Andrus AD, Ranieri ML, Orsi RH, Ivy R, Den Bakker HC (2014). Genomic comparison of sporeforming bacilli isolated from milk. BMC Genom.

[176] Sattin E, Andreani NA, Carraro L, Fasolato L, Balzan S, Novelli E (2016). Microbial dynamics during shelf-life of industrial Ricotta cheese and identification of a *Bacillus* strain as a cause of a pink discolouration. Food Microbiol.

