# Supplementary material for: Current knowledge and perspectives of Paenibacillus: a review
Source: Microb Cell Fact. 2016 Dec 1;15:203. doi: 10.1186/s12934-016-0603-7 (PMC5134293; doi:10.1186/s12934-016-0603-7)
Supplement: Supplementary file 1 — Additional file 1. All discovered Paenibacillus species along with their countries, environments, and years of isolation. [file 12934_2016_603_MOESM1_ESM.docx]

**Additional File 1.**All discovered *Paenibacillus* species along with their countries, environments, and years of isolation.

| **Species name** | **Year of initial isolation** | **Countries of isolation** | **Environments of isolation** | **References** |
| --- | --- | --- | --- | --- |
| P. abyssi | 2014 | India | Indian Ocean sediment sample | [1] |
| P. aestuarii | 2010 | Korea | Estaurine wetlands of Han river | [2] |
| P. agarexedens | 1941 | Germany | Meadow soil in Göttingen | [3] |
| P. agaridevorans | 2003 | Mexico | Volcanic soil, Paricutin Volcano | [3] |
| P. alba  P. algeriensis | 2015  2012 | Russia  Algeria | Peat soil  Hypersaline soil | [4]  [5] |
| P. alginolyticus | 1987 | United States | Soil | [6] |
| P. algorifonticola | 2011 | China | Cold spring water sample | [7] |
| P. alkaliterrae | 2005 | Korea | Alkaline soil in Kwangchun | [8] |
| P. alvei | 1885 | Germany, United States, India, Malaysia | Infected Honeybee colonies, soil, milk, human kidney, fermented tomato fruit | [9-13] |
| P. amylolyticus | 1984 | Denmark, Germany | Plant rhizospheres | [14] |
| P. anaericanus | 2005 | Germany, Korea | Gut of the earthworm *Aporrectodea caliginosa,* ginseng field soil | [15, 16] |
| P. antarcticus  P. antibioticophila | 2004  2015 | Antarctica  France | Sediment collected in Chlorite Lake  Human Feces | [17]  [18] |
| P. apiarius | 1955 | Canada | Honeybee larvae | [19] |
| P. assamensis | 2005 | India | Warm spring located in a reserve forest in Assam | [20] |
| P. azoreducens | 2001 | Ireland | Textile industry wastewate | [21] |
| P. barcinonensis | 2005 | Spain | Ebro's river delta | [22-25] |
| P. barengoltzii | 2006 | United States | Space craft assembly facility | [26] |
| P. beijingensis | 2013 | China | Wheat soil rhizosphere, Jujube garden soil | [27, 28] |
| P. borealis  P. bovis | 2001  2016 | Finland  Tibet | Spruce forest humus  Raw yak milk | [29]  [30] |
| P. brasilensis | 2002 | Brazil | Rhizospheres of maize and *Kalanchoe brasilensis* | [31, 32] |
| P. brassicae | 2012 | China | Cabbage rhizosphere | [33] |
| P. camelliae  P. camerounensis | 2008  2016 | Korea  Cameroon | Fermented leaves of *Camellia sinensis* (Pu'er tea)  Wild gorilla stool | [34]  [35] |
| P. campinasensis | 1998 | Brazil | Soil | [36] |
| P. castaneae | 2008 | Spain | Phyllosphere of *Castanea sativa* | [37] |
| P. catalpa  P. cathormii  P. cavernae | 2013  2016  2016 | China  Thailand  Korea | Rhizosphere soil of *Catalpa speciose*  Tree bark  Natural cave soil | [38]  [39]  [40] |
| P. cellulositrophicus | 2009 | Thailand | Soil | [41] |
| P. cellulosilyticus | 2006 | Spain | Bract phyllosphere of *Phoenix dactylifera* | [42] |
| P. chartarius | 2012 | Sweden | Paper mill | [43] |
| P. chibensis  P. chinensis | 1997  2016 | Japan  China | Soil  Maize seeds | [44]  [45] |
| P. chinjuensis | 2002 | Korea | Soil sample from Chinju | [46] |
| P. chitinolyticus | 1996 | Iran, Japan | Garden soil | [47, 48] |
| P. chondroitinus | 1987 | United States | Soil | [6] |
| P. chungangensis | 2011 | Korea | Tidal-flat sediment of DaeYiJac Island | [49] |
| P. cineris | 2004 | Antarctica, Brazil | Soil taken from an active fumarole, lungs of a Cystic Fibrosis patient | [50, 51] |
| P. contaminans | 2009 | China | Contaminated laboratory plate | [52] |
| P. cookii | 2004 | Antarctica, Belgium | Soil taken from an active fumarole, gelatin processing plant | [50] |
| P. cucumis | 2014 | Korea | Cucumber greenhouse soil in Cheongsong | [53] |
| P. curdlanotyticus | 1995 | Thailand, Japan | Anaerobic digester which was fed pineapple waste, soil | [6, 54, 55] |
| P. daejeonensis  P. dakarensis | 2002  2016 | Korea  Senegal | Alkaline soil in Daejeon  Sixteen month old child blood | [56]  [57] |
| P. darwinianus | 2012 | Antarctica | Gamma-irradiated soils of the Britannia drift, Lake Wellman Region | [58] |
| P. dauci | 2014 | China | Carrot samples | [59] |
| P. dendritiformis | 1999 | Israel, United States | Laboratory | [60-62] |
| P. dongdonensis | 2014 | Korea | *Elymus tsukushiensis* rhizosphere in Dongdo | [63] |
| P. donghaensis | 2008 | Korea | Deep-sea sediment | [64] |
| P. doosanensis | 2014 | Korea | Rhizospheric soil of *Elymus tsukushiensis* | [65] |
| P. durus | 1974 | India, Brazil | Oil mill soil, rhizospheres of maize, sorghum, sugarcane, wheat, banana, forage grasses and from bulk soil | [66, 67] |
| P. edaphicus | 1998 | China, India | Rhizosphere of cotton and other soils | [68, 69] |
| P. ehimensis | 1996 | China, Japan, Russia, Korea | Dairy waste, chitin enriched soil, pepper field soil | [70-75] |
| P. elgii | 2004 | Korea | *Perilla frutescens* soil, tomato soil | [76, 77] |
| P. endophyticus  P. enshidis  P. etheri  P. faecis | 2013  2015  2016  2015 | Spain  China  Spain  France | *Cicer arietinum* root nodules  *Robinia pseudoacacia* L. nodules  Hydrocarbon-contaminated soil  Human Feces | [78]  [79]  [80]  [81] |
| P. favisporus | 2004 | Spain, Brazil | Cow feces, soil from sugarcane plantation | [82, 83] |
| P. ferrarius | 2015 | China | Iron mineral soil | [84] |
| P. filicis | 2010 | Korea | Rhizosphere of ferns in Daejeon | [85] |
| P. fonticola | 2007 | Taiwan | Warm spring in Jhonglun | [86] |
| P. forsythiae | 2008 | China | *Forsythia mira* rhizosphere soil | [87] |
| P. frigoriresistens | 2012 | China | Peat bog sample | [88] |
| P. gansuensis  P. gelatinilyticus | 2006  2015 | China  Korea | Desert-soil sample  Reclaimed soil | [89]  [90] |
| P. ginsengarvi | 2007 | Korea | Ginseng field soil | [91] |
| P. ginsengihumi | 2008 | Korea | Ginseng field soil | [92] |
| P. ginsengiterrae | 2014 | Korea | Ginseng field soil | [93] |
| P. glacialis | 2010 | India | Soil sample near Kafni glacier | [94] |
| P. glucanolyticus | 1989 | United States, Germany | Black liquor, recycled cardboard and paper products, garden soil | [95-97] |
| P. glycanilyticus | 2002 | Japan | Soil | [98, 99] |
| P. gorillae | 2011 | Cameroon | Gorilla fecal sample | [100] |
| P. graminis | 2002 | France | Soil, plant rhizospheres, plant roots and pasteurized pureed vegetables | [101] |
| P. granivorans | 2001 | The Netherlands | Laboratory reactor fed with potato starch waste water | [102] |
| P. guangzhouensis | 2014 | China | Forest soil | [103] |
| P. harenae  P. hemerocallicola  P. herberti | 2009  2015  2015 | China  Korea  Tibet | Desert sand  Roots of herbaceous plants  *Herbertus sendtneri* | [104]  [105]  [106] |
| P. hodogayensis | 2005 | Japan | Activated sludge inhabited by *Sphaerotilus natans* | [107] |
| P. hongkongensis | 2003 | China | Contaminated blood sample | [108] |
| P. hordei | 2012 | Korea | Naked barley | [109] |
| P. humi | 2014 | Russia | Peat soil near coalmine | [110] |
| P. humicus | 2007 | Portugal, Japan | Poultry litter compost, fermented soybeans | [111, 112] |
| P. hunanensis  P. ihumii | 2010  2016 | India, China  France | Soil, seeds of hybrid rice  Stool sample from morbidly obese patient | [113, 114]  [115] |
| P. illinoisensis  P. insulae | 1997  2015 | Spain  Dokdo Island | Spanish-style green olive fermentation  Soil | [116]  [117] |
| P. jamilae | 2001 | China, Spain, India | Plant rhizospheres, compost prepared with olive-mill wastewaters, midgut of fifth instar larvae of *Helicoverpa armigera* | [118-120] |
| P. jilunlii | 2011 | China | Rhizosphere soil of *Begonia semperflorens* | [121] |
| P. kobensis | 1995 | Japan, Germany, United states | Alluvial soil, recycled cardboard and paper products, soil | [55, 96, 97] |
| P. koleovorans | 2002 | Japan | Soil sample | [122] |
| P. konsidensis | 2008 | Korea | Patient blood sample | [123] |
| P. koreensis | 2000 | Korea | Compost | [124] |
| P. kribbensis  P. kyungheensis | 2003  2015 | Korea  Korea | Soil  Magnolia flowers | [125, 126]  [127] |
| P. lactis | 2004 | Belgium | Raw and heated milk | [128] |
| P. larvae | 1906 | Worldwide | Contaminated bee hives | [129] |
| P. lautus | 1984 | United States, India | Hot spring, oil sludge, soil | [96, 130, 131] |
| P. lemnae | 2015 | Thailand | Duckweed | [132] |
| P. lentimorbus | 1940 | Brazil, Japan | *Rhizospora mangle* rhizosphere, *Blitopertha orientalis* beetle larvae, cow milk | [133-135] |
| P. lentus | 1988 | United States, South America | Mixed soil samples | [136] |
| P. lupini | 2014 | Spain | *Lupinus albus* nodules | [137] |
| P. macerans | 1905 | India, United States | *Typha latifolia* rhizosphere, contaminated human blood sample | [138] |
| P. macquariensis  P. marchantiophytorum | 1966  2016 | Russia, India  Tibet | Boreal soil, soil mixed with petroleum products  *Herbertus sendtneri* liverwort | [139, 140]  [141] |
| P. marinisediminis | 2013 | Korea | Marine sediment | [142] |
| P. marinum | 2012 | Tunisia | Maine hot spring | [143] |
| P. massiliensis  P. medicaginis | 2002  2015 | France  Taiwan | Blood culture  Alfalfa root nodule | [144]  [145] |
| P. mendelii | 2005 | Czech Republic | Surface-sterilized garden pea seeds | [146] |
| P. montaniterrae | 2009 | Thailand, India | Soil, bauxite residue (red mud) | [147, 148] |
| P. motobuensis | 2005 | Japan | Compost sample | [149] |
| P. mucilaginosus | 1998 | China | Soil | [150] |
| P. nanensis | 2009 | Thailand | Soil | [151] |
| P. naphthalenovorans  P. nasutitermitis | 2002  2016 | United States  China | Salt marsh plant rhizosphere  Termite gut | [152]  [153] |
| P. nematophilus | 2003 | Ireland | Within nematode *Heterorhabditis* spp. | [154] |
| P. nicotianae | 2014 | China | Tobacco sample | [155] |
| P. oceanisediminis | 2013 | Korea | Marine sediment | [156] |
| P. odorifer  P. oenotherae | 2002  2015 | France  Korea | Soil, plant rhizospheres, plant roots and pasteurized pureed vegetables  Roots of herbaceous plants | [101]  [105] |
| P. pabuli | 1984 | Canada, Germany | Soil, recycled cardboard and paper products | [96, 97] |
| P. panacisoli  P. panaciterrae | 2006  2015 | Korea  Korea | Ginseng field soil  Ginseng-cultivated soil | [157]  [158] |
| P. pasadenensis | 2006 | United States | Space craft assembly facility | [26] |
| P. pectinilyticus | 2009 | Korea | Gut of *Diestrammena apicalis* | [159] |
| P. peoriae  P. periandrae | 1993  2016 | United States, Germany  Brazil | Soil, rotting leaves, recycled paper and coardboard products  *Periandra mediterranea* nodules | [96, 97]  [160] |
| P. phoenicis | 2005 | United States | NASA Phoenix Lander assembly facility, subsurface molybdenum mine | [161] |
| P. phyllosphaerae  P. physcomitrellae | 2005  2015 | Spain  China | Phyllosphere of *Phoenix dactylifera*  *Physcomitrella patens* moss | [162]  [163] |
| P. pinesoli | 2013 | Korea | Pine tree forest soil | [164] |
| P. pini | 2011 | Japan | Pine tree rhizosphere | [165] |
| P. pinihumi | 2010 | Korea | Rhizosphere of *Pinus densiflora* | [166] |
| P. pocheonensis | 2010 | Korea | Ginseng field soil | [167] |
| P. polymyxa | 1880 | China, Japan, Mexico, Paracel islands, Canada, Brazil, Korea, Egypt, Russia, Israel | Pine soil, Nono fruit, decaying sorghum straw, agricultural soil, corn root, sewage sludge, maize rhizosphere, winter barley root, wheat soil, rotten ginseng root, livestock probiotic feed supplement, turnip, pepper root/rhizosphere, intestinal tract of domestic Russian chicken, pine seedling stem, watermelon rhizosphere, apple orchard soil, blood sample of patient with bacteremia | [168-188] |
| P. popilliae  P. populi | 1940  2015 | United States, Japan  China | Variety of insect larvae, *Blitopertha orientalis* beetle larvae  *Populus alba* | [135, 189]  [190] |
| P. profundus | 2010 | Japan | Deep surface sediment sample | [191, 192] |
| P. prosopidis | 2010 | Tunisia | Root nodules of *Prosopis farcta* | [193] |
| P. provencensis | 2008 | France, Spawn | Human cerebrospinal fluid, contaminated homeopathic drugs | [194, 195] |
| P. pueri | 2009 | Korea | Pu'er tea made from *Camellia sinensi* leaves | [196] |
| P. puldeungensis | 2011 | Korea | Grassy sandbank | [197] |
| P. purispatii  P. qingshengii | 2011  2015 | Germany  China | Space craft assembly clean room  Lead-zinc tailing | [198]  [199] |
| P. quercus  P. radicis | 2014  2016 | China  China | *Quercas aliena* var. acuteserrata rhizosphere  Maize root | [200]  [201] |
| P. relictisesami | 2014 | Japan | Sesame oil cake | [202] |
| P. residui  P. rhizoryzae | 2010  2015 | Portugal  China | Urban waste compost  Rice rhizosphere | [203]  [204] |
| P. rhizosphaerae | 2005 | Spain | Rhizosphere of *Cicer arietinum* | [205] |
| P. rigui | 2011 | Korea | Freshwater collected from the Woopo wetland | [206] |
| P. riograndensis  P. ripae | 2010  2015 | Brazil  China | Rhizosphere of *Triticum aestivum*  Bank side soil | [207]  [208] |
| P. sabinae | 2007 | China | *Sabina squamata* rhizosphere | [209] |
| P. sacheonensis | 2011 | Korea | Tidal flat sediment from Sacheon Bay | [210] |
| P. sanguinis | 2002 | France | Blood culture | [144] |
| P. sediminis | 2012 | Korea | Tidal flat adjacent to Ganghwa Island | [211] |
| P. selenii | 2014 | China | Selenium mineral soil | [212] |
| P. selenitireducens | 2014 | China | Selenium mineral soil | [213] |
| P. senegalensis | 2012 | Senegal | Fecal sample from a healthy patient | [214] |
| P. septentrionalis | 2009 | Thailand | Soil | [147] |
| P. sepulcri | 2001 | Italy | Biodeteriorated mural paintings in the Servilia tomb of the Roman necropolis of Carmona | [215] |
| P. shenyangensis | 1996 | China | Peach tree soil | [216] |
| P. shirakamiensis | 1993 | Japan | Oak trunk surface | [217] |
| P. siamensis | 2009 | Thailand | Soil | [147] |
| P. soli | 2007 | Korea | Ginseng field soil | [218] |
| P. sonchi | 2009 | China | Rhizosphere soil of *Sonchus oleraceus* | [219] |
| P. sophorae | 2011 | China | Rhizosphere of *Sophora japonica* | [220] |
| P. sputi | 2010 | Korea | Sputum of patient with pulmonary disease | [221] |
| P. stellifer | 2003 | China, Finland | Plant rhizospheres, food-packaging paperboard | [118, 222] |
| P. susongensis | 2014 | China | Rock surfaces | [223] |
| P. swuensis | 2014 | Korea | Soil | [224] |
| P. taichungensis | 2008 | Korea, Taiwan | Arsenic tainted and non-tainted soils | [225, 226] |
| P. taihuensis | 2010 | China | Decomposing algal scum | [227] |
| P. taiwanensis | 2007 | Taiwan | Farmland soil in Wu-Feng | [228] |
| P. taohuashanense | 2012 | China | Rhizosphere soil sample of *Caragana kansuensis Pojark* | [229] |
| P. tarimensis | 2008 | China, Tunisia | Desert sand, sediment sample | [230, 231] |
| P. telluris | 2010 | Korea | Farm soil | [232] |
| P. terrae  P. terreus | 2003  2016 | China, Germany  China | Soil sample, gut of the earthworm *Aporrectodea caliginosa*  Forest soil | [15, 233]  [234] |
| P. terrigena | 2007 | Japan | Chiba coastal soil | [235] |
| P. tezpurensis | 2009 | India | Soil sample | [236] |
| P. thailandensis | 2009 | Thailand | Soil | [151] |
| P. thermoaerophilus | 2013 | Japan | Compost sample | [237] |
| P. thermophilus | 2012 | China | Hot spring sediment sample | [238] |
| P. thiaminolyticus | 1990 | United States | Soil Sample, blood sample from human with renal failure | [239, 240] |
| P. tianmuensis  P. tibetensis | 2011  2015 | China  Tibet | Soil samples of Tianmu Mountai  Alpine swamp meadow soil | [241]  [242] |
| P. timonensis | 2002 | France | Blood culture of woman with chronic interstitial nephropathy | [144] |
| P. tundrae | 2009 | United States (Alaska) | Soil beneath moist non-acidic and acidic tundra | [243] |
| P. turicensis  P. tylopili | 2002  2008 | Switzerland  Lithuania | Valve of a cerebrospinal fluid shunt of a 48-year-old man  *Tylopilus felleus* mycorhizosphere | [244]  [245] |
| P. typhae | 2013 | China | Roots of *Typha angustifolia L.* | [241] |
| P. tyraminigenes | 2007 | Korea | Myeolchi-jeotgal, a traditional Korean salted and fermented anchovy | [246] |
| P. uliginis | 2011 | Germany | Fen peat soil | [198] |
| P. urinalis | 2008 | France | Human urine of patient with pulmonary infection | [194] |
| P. validus  P. vini  P. vortex | 1984  2015  1994 | Iran, India, Antarctica, Spain  China  Netherlands | industrial effluent-polluted soil, geothermal soils on  Alcohol Fermentation Pit mud  Mount Melbourne, soil  Outgrowth from *Bacillus subtilis* colony | [96, 247-249]  [250]  [251] |
| P. vulneris  P. wenxiniae | 2006  2015 | Norway  China | Necrotic wound of a 35-year-old man  Maize | [252]  [253] |
| P. wooponensis | 2011 | Korea | Fresh water sample collected from Woopo wetland | [254] |
| P. woosongensis | 2008 | Korea, India | Forest soil, soil from chicken feather dumping site | [255, 256] |
| P. wulumuqiensis | 2014 | China | Cold spring | [59] |
| P. wynnii  P. xanthinilyticus | 2005  2015 | Antarctica  Korea | Soil from Mars Oasis  Agricultural soil | [257]  [258] |
| P. xinjiangensis | 2006 | China | Alkaline soil | [259] |
| P. xylanexedens | 2009 | United States | Soil beneath moist non-acidic and acidic tundra in northern Alaska | [243] |
| P. xylaniclasticus | 2011 | Thailand | Sludge in an anerobic digester fed with pineapple waste | [260] |
| P. xylanisolvens | 2011 | Thailand | Soil | [261] |
| P. xylanilyticus | 2005 | Spain | Spanish-style green olive fermentation | [116] |
| P. yonginensis  P. yunnanensis | 2014  2015 | Korea  China | Humus soil of Yongin forest  Pu’er tea | [262]  [263] |
| P. zanthoxyli  P. zeae | 2007  2015 | China  Korea | *Zanthoxylum simulans* Rhizosphere  Maize seeds | [264]  [265] |

1. Huang XF, Wang FZ, Zhang W, Li J, Ling J, Yang J, et al. Paenibacillus abyssi sp. nov., isolated from an abyssal sediment sample from the Indian Ocean. Antonie Van Leeuwenhoek Int J Gen Mol Microbiol. 2014;106:1089-95.

2. Bae JY, Kim KY, Kim JH, Lee K, Cho JC, Cha CJ. Paenibacillus aestuarii sp. nov., isolated from an estuarine wetland. Int J Syst Evol Microbiol. 2010;60:644-7.

3. Uetanabaro AP, Wahrenburg C, Hunger W, Pukall R, Spröer C, Stackebrandt E, et al. Paenibacillus agarexedens sp. nov., nom. rev., and Paenibacillus agaridevorans sp. nov. Int J Syst Evol Microbiol. 2003;53:1051-7.

4. Kim HS, Srinivasan S, Lee SS. Paenibacillus alba nov., Isolated from Peat Soil. Curr Microbiol. 2015;70:865-70.

5. Bendjama E, Loucif L, Diene SM, Michelle C, Gacemi-Kirane D, Rolain J-M. Non-contiguous finished genome sequence and description of Paucisalibacillus algeriensis sp nov. Stand Genomic Sci. 2014;9:1352-65.

6. Nakamura LK. Bacillus alginolyticus sp. nov. and Bacillus chondroitinus sp. nov., Two Alginate-Degrading Species. Int J Syst Bacteriol. 1987;37:284-6.

7. Tang QY, Yang N, Wang J, Xie YQ, Ren B, Zhou YG, et al. Paenibacillus algorifonticola sp. nov., isolated from a cold spring. Int J Syst Evol Microbiol. 2011;61:2167-72.

8. Yoon JH, kang SJ, Yeo SH, Oh TK. Paenibacillus alkaliterrae sp. nov., isolated from an alkaline soil in Korea. Int J Syst Evol Microbiol. 2005;55:2339-44.

9. Erler S, Denner A, Bobiş O, Forsgren E, Moritz RFA. Diversity of honey stores and their impact on pathogenic bacteria of the honeybee, Apis mellifera. Ecology and Evolution. 2014;4:3960-7.

10. Yi T, Huang Y, Chen Y. Production of an antimicrobial peptide an5-1 in Escherichia coli and its dual mechanisms against bacteria. Chem Biol Drug Des. 2015;85:598-607.

11. Padhi S, Dash M, Sahu R, Panda P. Urinary Tract Infection due to Paenibacillus alvei in a Chronic Kidney Disease: A Rare Case Report. Journal of Laboratory Physicians. 2013;5:133-5.

12. Anandaraj B, Vellaichamy A, Kachman M, Selvamanikandan A, Pegu S, Murugan V. Co-production of two new peptide antibiotics by a bacterial isolate Paenibacillus alvei NP75. Biochem Biophys Res Commun. 2009;379:179-85.

13. Alkotaini B, Anuar N, Kadhum AAH, Sani AAA. Detection of secreted antimicrobial peptides isolated from cell-free culture supernatant of Paenibacillus alvei AN5. J Ind Microbiol Biotechnol. 2013;40:571-9.

14. Validov S, Kamilova F, Qi S, Stephan D, Wang JJ, Makarova N, et al. Selection of bacteria able to control Fusarium oxysporum f. sp. radicis-lycopersici in stonewool substrate. J Appl Microbiol. 2007;102:461-71.

15. Horn MA, Ihssen J, Matthies C, Schramm A, Acker G, Drake HL. Dechloromonas denitrificans sp. nov., Flavobacterium denitrificans sp. nov., Paenibacillus anaericanus sp. nov. and Paenibacillus terrae strain MH72, N2O-producing bacteria isolated from the gut of the earthworm Aporrectodea caliginosa. Int J Syst Evol Microbiol. 2005;55:1255-65.

16. Lee M, Ten LN, Baek SH, Im WT, Aslam Z, Lee ST. Paenibacillus ginsengisoli sp. nov., a novel bacterium isolated from soil of a ginseng field in Pocheon province, South Korea. Antonie Van Leeuwenhoek Int J Gen Mol Microbiol. 2007;91:127-35.

17. Montes MJ, Mercadé E, Bozal N, Guinea J. Paenibacillus antarcticus sp. nov., a novel psychrotolerant organism from the Antarctic environment. Int J Syst Evol Microbiol. 2004;54:1521-6.

18. Dubourg G, Cimmino T, Senkar SA, Lagier JC, Robert C, Flaudrops C, et al. Noncontiguous finished genome sequence and description of Paenibacillus antibioticophila sp. nov. GD11T, the type strain of Paenibacillus antibioticophila. New Microbes New Infect. 2015;8:137-47.

19. Katznelson H. Bacillus apiarus, n. sp., an aerobic spore-forming organism isolated from honeybee larvae. J Bacteriol. 1955;70:635-6.

20. Saha P, Mondal AK, Mayilraj S, Krishnamurthi S, Bhattacharya A, Chakrabarti T. Paenibacillus assamensis sp. nov., a novel bacterium isolated from a warm spring in Assam, India. Int J Syst Evol Microbiol. 2005;55:2577-81.

21. Meehan C, Bjourson AJ, McMullan G. Paenibacillus azoreducens sp. nov., a synthetic azo dye decolorizing bacterium from industrial wastewater. Int J Syst Evol Microbiol. 2001;51:1681-5.

22. Sainz-Polo MA, Valenzuela SV, Pastor FJ, Sanz-Aparicio J. Crystallization and preliminary X-ray diffraction analysis of Xyn30D from Paenibacillus barcinonensis. Acta Crystallogr Sect F Struct Biol Commun. 2014;70:963-6.

23. Infanzón B, Valenzuela SV, Fillat A, Pastor FIJ, Diaz P. Unusual carboxylesterase bearing a GGG(A)X-type oxyanion hole discovered in Paenibacillus barcinonensis BP-23. Biochimie. 2014;104:108-16.

24. Valenzuela SV, Diaz P, Pastor FIJ. Xyn11E from Paenibacillus barcinonensis BP-23: A LppX-chaperone-dependent xylanase with potential for upgrading paper pulps. Appl Microbiol Biotechnol. 2014;98:5949-57.

25. Ciolacu D, Chiriac AI, Pastor FIJ, Kokol V. The influence of supramolecular structure of cellulose allomorphs on the interactions with cellulose-binding domain, CBD3b from Paenibacillus barcinonensis. Bioresour Technol. 2014;157:14-21.

26. Osman S, Satomi M, Venkateswaran K. Paenibacillus pasadenensis sp. nov. and Paenibacillus barengoltzii sp. nov., isolated from a spacecraft assembly facility. Int J Syst Evol Microbiol. 2006;56:1509-14.

27. Wang LY, Li J, Li QX, Chen SF. Paenibacillus beijingensis Sp. Nov., a nitrogen-fixing species isolated from wheat rhizosphere soil. Antonie Van Leeuwenhoek Int J Gen Mol Microbiol. 2013;104:675-83.

28. Gao M, Xie LQ, Wang YX, Chen J, Xu J, Zhang XX, et al. Paenibacillus beijingensis sp. nov., a novel nitrogen-fixing species isolated from jujube garden soil. Antonie Van Leeuwenhoek Int J Gen Mol Microbiol. 2012;102:689-94.

29. Elo S, Suominen I, Kämpfer P, Juhanoja J, Salkinoja-Salonen M, Haahtela K. Paenibacillus borealis sp. nov., a nitrogen-fixing species isolated from spruce forest humus in Finland. Int J Syst Evol Microbiol. 2001;51:535-45.

30. Gao C, Han J, Liu Z, Xu X, Hang F, Wu Z. Paenibacillus bovis sp. nov., isolated from raw yak (Bos grunniens) milk. Int J Syst Evol Microbiol. 2016;66:1413-8.

31. von der Weid I, Duarte GF, van Elsas JD, Seldin L. Paeninacillus brasilensis sp. nov., a novel nitrogen-fixing species isolated from the maize rhizosphere in Brazil. Int J Syst Evol Microbiol. 2002;52:2147-53.

32. Fortes TO, Alviano DS, Tupinambá G, Padrón TS, Antoniolli A, Alviano CS, et al. Production of an antimicrobial substance against Cryptococcus neoformans by Paenibacillus brasilensis Sa3 isolated from the rhizosphere of Kalanchoe brasiliensis. Microbiol Res. 2008;163:200-7.

33. Gao M, Yang H, Zhao J, Liu J, Sun YH, Wang YJ, et al. Paenibacillus brassicae sp. nov., isolated from cabbage rhizosphere in Beijing, China. Antonie Van Leeuwenhoek Int J Gen Mol Microbiol. 2013;103:647-53.

34. Oh HW, Kim BC, Lee KH, Kim DY, Park DS, Park HM, et al. Paenibacillus camelliae sp. nov., isolated from fermented leaves of Camellia sinensis. J Microbiol. 2008;46:530-4.

35. Keita MB, Padhmanabhan R, Robert C, Delaporte E, Raoult D, Fournier PE, et al. Non-contiguous-Finished Genome Sequence and Description of Paenibacillus camerounensis sp. nov. Microb Ecol. 2016;71:990-8.

36. Yoon J-H, Yim DK, Lee J-S, Shin K-S, Sato HH, Lee ST, et al. Paenibacillus campinasensis sp. nov., a cyclodextrin-producing bacterium isolated in Brazil. Int J Syst Bacteriol. 1998;48:833-7.

37. Valverde A, Peix A, Rivas R, Velázquez E, Salazar S, Santa-Regina I, et al. Paenibacillus castaneae sp. nov., isolaled from the phyllosphere of Castanea sativa Miller. Int J Syst Evol Microbiol. 2008;58:2560-4.

38. Zhang J, Wang ZT, Yu HM, Ma Y. Paenibacillus catalpae sp. nov., isolated from the rhizosphere soil of Catalpa speciosa. Int J Syst Evol Microbiol. 2013;63:1776-81.

39. Sitdhipol J, Paek J, Sin Y, Park IS, Thamacharoensuk T, Wannissorn B, et al. Paenibacillus cathormii sp. nov., isolated from tree bark. Int J Syst Evol Microbiol. 2016;66:1187-92.

40. Dong Lee S. Paenibacillus cavernae sp. Nov., Isolated from soil of a natural cave. Int J Syst Evol Microbiol. 2016;66:598-603.

41. Akaracharanya A, Lorliam W, Tanasupawat S, Lee KC, Lee JS. Paenibacillus cellulositrophicus sp. nov., a cellulolytic bacterium from Thai soil. Int J Syst Evol Microbiol. 2009;59:2680-4.

42. Rivas R, Garciá-Fraile P, Mateos PF, Martínez-Molina E, Velázquez E. Paenibacillus cellulosilyticus sp. nov., a cellulolytic and xylanolytic bacterium isolated from the bract phyllosphere of Phoenix dactylifera. Int J Syst Evol Microbiol. 2006;56:2777-81.

43. Kämpfer P, Falsen E, Lodders N, Martin K, Kassmannhuber J, Busse HJ. Paenibacillus chartarius sp. nov., isolated from a paper mill. Int J Syst Evol Microbiol. 2012;62:1342-7.

44. Shida O, Takagi H, Kadowaki K, Nakamura LK, Komagata K. Emended Description of Paenibacillus amylolyticus and Description of Paenibacillus illinoisensis sp. nov. and Paenibacillus chibensis sp. nov. Int J Syst Bacteriol. 1997;47:299-306.

45. Liu Y, Zhao R, Wang R, Yao S, Zhai L, Zhang X, et al. Paenibacillus chinensis sp. nov., isolated from maize (Zea mays L.) seeds. Antonie Van Leeuwenhoek Int J Gen Mol Microbiol. 2016;109:207-13.

46. Yoon JH, Seo WT, Shin YK, Kho YH, Kang KH, Park YH. Paenibacillus chinjuensis sp. nov., a novel exopolysaccharide-producing bacterium. Int J Syst Evol Microbiol. 2002;52:415-21.

47. Jami Al Ahmadi K, Tabatabaei Yazdi M, Fathi Najafi M, Shahverdi AR, Faramarzi MA, Zarrini G, et al. Isolation and characterization of a chitionolytic enzyme producing microorganism, Paenibacillus chitinolyticus JK2 from Iran. Res J Microbiol. 2008;3:395-404.

48. Kuroshima K-I, Sakane T, Takata R, Yokota A. Bacillus ehimensis sp. nov. and Bacillus chitinolyticus sp. nov., new chitinolytic members of the genus Bacillus. Int J Syst Bacteriol. 1996;46:76-80.

49. Park MH, Traiwan J, Jung MY, Nam YS, Jeong JH, Kim W. Paenibacillus chungangensis sp. nov., isolated from a tidal-flat sediment. Int J Syst Evol Microbiol. 2011;61:281-5.

50. Logan NA, De Clerck E, Lebbe L, Verhelst A, Goris J, Forsyth G, et al. Paenibacillus cineris sp. nov. and Paenibacillus cookii sp. nov., from Antarctic volcanic soils and a gelatin-processing plant. Int J Syst Evol Microbiol. 2004;54:1071-6.

51. Leão RS, Pereira RHV, Ferreira AG, Lima AN, Albano RM, Marques EA. First report of Paenibacillus cineris from a patient with cystic fibrosis. Diagn Microbiol Infect Dis. 2009;66:101-3.

52. Chou JH, Lee JH, Lin MC, Chang PS, Arun AB, Young CC, et al. Paenibacillus contaminans sp. nov., isolated from a contaminated laboratory plate. Int J Syst Evol Microbiol. 2009;59:125-9.

53. Ahn JH, Kim BC, Kim BY, Kim SJ, Song J, Kwon SW, et al. Paenibacillus cucumis sp. nov. isolated from greenhouse soil. J Microbiol. 2014;52:460-4.

54. Sermsathanaswadi J, Pianwanit S, Pason P, Waeonukul R, Tachaapaikoon C, Ratanakhanokchai K, et al. The C-terminal region of xylanase domain in Xyn11A from Paenibacillus curdlanolyticus B-6 plays an important role in structural stability. Appl Microbiol Biotechnol. 2014;98:8223-33.

55. Mera N, Iwasaki K. Use of plate-wash samples to monitor the fates of culturable bacteria in mercury- and trichloroethylene-contaminated soils. Appl Microbiol Biotechnol. 2007;77:437-45.

56. Lee J-S, Lee KC, Chang Y-H, Hong SG, Oh HW, Pyun Y-R, et al. Paenibacillus daejeonensis sp. nov., a novel alkaliphilic bacterium from soil. Int J Syst Evol Microbiol. 2002;52:2107-11.

57. Lo CI, Sankar SA, Fall B, Sambe-Ba B, Mediannikov O, Robert C, et al. High-quality genome sequence and description of Paenibacillus dakarensis sp. nov. New Microbes New Infect. 2016;10:132-41.

58. Dsouza M, Taylor MW, Ryan J, MacKenzie A, Lagutin K, Anderson RF, et al. Paenibacillus darwinianus sp. nov., isolated from gamma-irradiated Antarctic soil. Int J Syst Evol Microbiol. 2014;64:1406-11.

59. Zhu J, Wang W, Li SH, Song SQ, Xie YQ, Tang QY, et al. Paenibacillus wulumuqiensis sp. nov. and Paenibacillus dauci sp. nov., two novel species of the genus Paenibacillus. Arch Microbiol. 2015;197:489-95.

60. Tcherpakov M, Ben-Jacob E, Gutnick DL. Paenibacillus dendritiformis sp. nov., proposal for a new pattern-forming species and its localization within a phylogenetic cluster. Int J Syst Bacteriol. 1999;49:239-46.

61. Be'er A, Ariel G, Kalisman O, Helman Y, Sirota-Madi A, Zhang HP, et al. Lethal protein produced in response to competition between sibling bacterial colonies. Proc Natl Acad Sci U S A. 2010;107:6258-63.

62. Be'er A, Zhang HP, Florin EL, Payne SM, Ben-Jacob E, Swinney HL. Deadly competition between sibling bacterial colonies. Proc Natl Acad Sci U S A. 2009;106:428-33.

63. Son JS, Kang HU, Ghim SY. Paenibacillus dongdonensis sp. nov., isolated from rhizospheric soil of Elymus tsukushiensis. Int J Syst Evol Microbiol. 2014;64:2865-70.

64. Choi JH, Im WT, Yoo JS, Lee SM, Moon DS, Kim HJ, et al. Paenibacillus donghaensis sp. nov., a Xylan-degrading and nitrogen-fixing bacterium isolated from east sea sediment. J Microbiol Biotechnol. 2008;18:189-93.

65. Kim JH, Kang H, Kim W. Paenibacillus doosanensis sp. nov., isolated from soil. Int J Syst Evol Microbiol. 2014;64:1271-7.

66. Seldin L, Rosado AS, Cruz DWd, Nobrega A, Elsas JDv, Paiva E. Comparison of Paenibacillus azotofixans strains isolated from rhizoplane, rhizosphere, and non-root-associated soil from maize planted in two different Brazilian soils. Appl Environ Microbiol. 1998;64:3860-8.

67. Hungund B, Shyama VS, Patwardhan P, Saleh AM. Production of polyhydroxyalkanoate from paenibacillus durus BV-1 isolated from oil mill soil. J Microb Biochem Technol. 2013;5:13-7.

68. Sheng XF, Jiang CY, He LY. Characterization of plant growth-promoting Bacillus edaphicus NBT and its effect on lead uptake by Indian mustard in a lead-amended soil. Can J Microbiol. 2008;54:417-22.

69. Meena VS, Maurya BR, Verma JP. Does a rhizospheric microorganism enhance K+ availability in agricultural soils? Microbiol Res. 2014;169:337-47.

70. Aktuganov G, Jokela J, Kivelä H, Khalikova E, Melentjev A, Galimzianova N, et al. Isolation and identification of cyclic lipopeptides from Paenibacillus ehimensis, strain IB-X-b. J Chromatogr B Anal Technol Biomed Life Sci. 2014;973:9-16.

71. Aktuganov G, Melentjev A, Galimzianova N, Khalikova E, Korpela T, Susi P. Wide-range antifungal antagonism of Paenibacillus ehimensis IB-X-b and its dependence on chitinase and β-1,3-glucanase production. Can J Microbiol. 2008;54:577-87.

72. De Araújo NK, De Assis CF, Dos Santos ES, De Macedo GR, De Farias LF, Arimatéia Jr H, et al. Production of enzymes by paenibacillus chitinolyticus and paenibacillus ehimensis to obtain chitooligosaccharides. Appl Biochem Biotechnol. 2013;170:292-300.

73. Huang Z, Hu Y, Shou L, Song M. Isolation and partial characterization of cyclic lipopeptide antibiotics produced by Paenibacillus ehimensis B7. BMC Microbiol. 2013;13:87.

74. Naing KW, Nguyen XH, Anees M, Lee YS, Kim YC, Kim SJ, et al. Biocontrol of Fusarium wilt disease in tomato by Paenibacillus ehimensis KWN38. World J Microbiol Biotechnol. 2014;31:165-74.

75. Naing KW, Anees M, Kim SJ, Nam Y, Kim YC, Kim KY. Characterization of antifungal activity of Paenibacillus ehimensis KWN38 against soilborne phytopathogenic fungi belonging to various taxonomic groups. Ann Microbiol. 2014;64:55-63.

76. Nguyen XH, Naing KW, Lee YS, Moon JH, Lee JH, Kim KY. Isolation and characteristics of protocatechuic acid from Paenibacillus elgii HOA73 against Botrytis cinerea on strawberry fruits. J Basic Microbiol. 2015;55:625-34.

77. Kim DS, Bae CY, Jeon JJ, Chun SJ, Oh HW, Hong SG, et al. Paenibacillus elgii sp. nov., with broad antimicrobial activity. Int J Syst Evol Microbiol. 2004;54:2031-5.

78. Carro L, Flores-Félix JD, Cerda-Castillo E, Ramírez-Bahena MH, Igual JM, Tejedor C, et al. Paenibacillus endophyticus sp. nov., isolated from nodules of Cicer arietinum. Int J Syst Evol Microbiol. 2013;63:4433-8.

79. Yin J, He D, Li X, Zeng X, Tian M, Cheng G. Paenibacillus enshidis sp. nov., Isolated from the Nodules of Robinia pseudoacacia L. Curr Microbiol. 2015;71:321-5.

80. Guisado IM, Purswani J, González-López J, Pozo C. Paenibacillus etheri sp. nov., able to grow on media supplemented with methyl tert-butyl ether (MTBE) and isolated from hydrocarbon-contaminated soil. Int J Syst Evol Microbiol. 2016;66:862-7.

81. Clermont D, Gomard M, Hamon S, Bonne I, Fernandez JC, Wheeler R, et al. Paenibacillus faecis sp nov, isolated from human faeces. Int J Syst Evol Microbiol. 2015;65:4621-6.

82. Velázquez E, de Miguel T, Poza M, Rivas R, Rosselló-Mora R, Villa TG. Paenibacillus favisporous sp. nov., a xylanolytic bacterium isolated from cow faeces. Int J Syst Evol Microbiol. 2004;54:59-64.

83. Padilha IQM, Valenzuela SV, Grisi TCSL, Diaz P, Araujo DAMd, Pastor FIJ. A glucuronoxylan-specific xylanase from a new Paenibacillus favisporus strain isolated from tropical soil of Brazil. Int Microbiol. 2014;17:175-84.

84. Cao Y, Chen F, Li Y, Wei S, Wang G. Paenibacillus ferrarius sp. nov., isolated from iron mineral soil. Int J Syst Evol Microbiol. 2015;65:165-70.

85. Kim BC, Kim MN, Lee KH, Kwon SB, Bae KS, Shin KS. Paenibacillus filicis sp. nov., isolated from the rhizosphere of the fern. J Microbiol. 2009;47:524-9.

86. Chou JH, Chou YJ, Lin KY, Sheu SY, Sheu DS, Arun AB, et al. Paenibacillus fonticola sp. nov., isolated from a warm spring. Int J Syst Evol Microbiol. 2007;57:1346-50.

87. Ma YC, Chen SF. Paenibacillus forsythiae sp. nov., a nitrogen-fixing species isolated from rhizosphere soil of Forsythia mira. Int J Syst Evol Microbiol. 2008;58:319-23.

88. Ming H, Nie GX, Jiang HC, Yu TT, Zhou EM, Feng HG, et al. Paenibacillus frigoriresistens sp. nov., a novel psychrotroph isolated from a peat bog in heilongjiang, northern china. Antonie Van Leeuwenhoek Int J Gen Mol Microbiol. 2012;102:297-305.

89. Lim J-M, Jeon CO, Lee J-C, Xu L-H, Jiang C-L, Kim C-J. Paenibacillus gansuensis sp. nov., isolated from desert soil of Gansu Province in China. Int J Syst Evol Microbiol. 2006;56:2131-4.

90. Padakandla SR, Lee GW, Chae JC. Paenibacillus gelatinilyticus sp. nov. a psychrotolerant bacterium isolated from a reclaimed soil and amended description of Paenibacillus shenyangensis. Antonie Van Leeuwenhoek Int J Gen Mol Microbiol. 2015;108:1197-203.

91. Yoon M-H, Ten LN, Im W-T. Paenibacillus ginsengarvi sp. nov., isolated from soil from ginseng cultivation. Int J Syst Evol Microbiol. 2007;57:1810-4.

92. Kim MK, Kim YA, Park MJ, Yang DC. Paenibacillus ginsengihumi sp. nov., a bacterium isolated from soil in a ginseng field. Int J Syst Evol Microbiol. 2008;58:1164-8.

93. Huq MA, Kim YJ, Hoang VA, Siddiqi MZ, Yang DC. Paenibacillus ginsengiterrae sp. nov., a ginsenoside-hydrolyzing bacteria isolated from soil of ginseng field. Arch Microbiol. 2015;197:389-96.

94. Kishore KH, Begum Z, Pathan AAK, Shivaji S. Paenibacillus glacialis sp. nov., isolated from the Kafni glacier of the Himalayas, India. Int J Syst Evol Microbiol. 2010;60:1909-13.

95. Mathews SL, Pawlak JJ, Grunden AM. Isolation of Paenibacillus glucanolyticus from pulp mill sources with potential to deconstruct pulping waste. Bioresour Technol. 2014;164:100-5.

96. Shida O, Takagi H, Kadowaki K, Nakamura I, Komagata K. Transfer of Bacillus alginolyticus, Bacillus chondroitinus, Bacillus curdlanolyticus, Bacillus glucanolyticus, Bacillus kobensis, and Bacillus thiaminolyticus to the Genus Paenibacillus and Emended Description of the Genus Paenibacillus. Int J Syst Bacteriol. 1997;47:289-98.

97. Suihko ML, Stackebrandt E. Identification of aerobic mesophilic bacilli isolated from board and paper products containing recycled fibres. J Appl Microbiol. 2003;94:25-34.

98. Suyotha W, Yano S, Itoh T, Fujimoto H, Hibi T, Tachiki T, et al. Characterization of α-1,3-glucanase isozyme from Paenibacillus glycanilyticus FH11 in a new subgroup of family 87 α-1,3-glucanase. J Biosci Bioeng. 2014;118:378-85.

99. Dasman A, Kajiyama S, Kawasaki H, Yagi M, Seki T, Fukusaki EI, et al. Paenibacillus glycanilyticus sp. nov., a novel species that degrades heteropolysaccharide produced by the cyanobacterium Nostoc commune. Int J Syst Evol Microbiol. 2002;52:1669-74.

100. Keita MB, Padhmananabhan R, Caputo A, Robert C, Delaporte E, Raoult D, et al. Non-contiguous finished genome sequence and description of Paenibacillus gorillae sp nov. Stand Genomic Sci. 2014;9:1031-45.

101. Berge O, Guinebretiere MH, Achouak W, Normand P, Heulin T. Paenibacillus graminis sp. nov. and Paenibacillus odorifer sp. nov., isolated from plant roots, soil and food. Int J Syst Evol Microbiol. 2002;52:607-16.

102. van der Maarel MJEC, Veen A, Wijbenga DJ. Paenibacillus granivorans sp. nov., a new Paenibacillus Species which Degrades Native Potato Starch Granules. Syst Appl Microbiol. 2000;23:344-8.

103. Li JB, Lu Q, Liu T, Zhou SG, Yang GQ, Zhao Y. Paenibacillus guangzhouensis sp. nov., An Fe(iii)-and humus-reducing bacterium from a forest soil. Int J Syst Evol Microbiol. 2014;64:3891-6.

104. Jeon CO, Lim JM, Lee SS, Chung BS, Park DJ, Xu LH, et al. Paenibacillus harenae sp. nov., isolated from desert sand in China. Int J Syst Evol Microbiol. 2009;59:13-7.

105. Kim TS, Han JH, Joung Y, Kim SB. Paenibacillus oenotherae sp. Nov. and Paenibacillus hemerocallicola sp. nov., isolated from the roots of herbaceous plants. Int J Syst Evol Microbiol. 2015;65:2717-25.

106. Guo GN, Zhou X, Zhao R, Chen XY, Chen ZL, Li XD, et al. Paenibacillus herberti sp. nov., an endophyte isolated from Herbertus sendtneri. Antonie Van Leeuwenhoek Int J Gen Mol Microbiol. 2015;108:587-96.

107. Takeda M, Suzuki I, Koizumi J-i. Paenibacillus hodogayensis sp. nov., capable of degrading the polysaccharide produced by Sphaerotilus natans. Int J Syst Evol Microbiol. 2005;55:737-41.

108. Teng JLL, Woo PCY, Leung KW, Lau SKP, Wong MKM, Yuen KY. Pseudobacteraemia in a patient with neutropenic fever caused by a novel paenibacillus species: Paenibacillus hongkongensis sp. nov. Molecular Pathology. 2003;56:29-35.

109. Kim JM, Lee SH, Lee SH, Choi EJ, Jeon CO. Paenibacillus hordei sp. nov., isolated from naked barley in Korea. Antonie Van Leeuwenhoek. 2013;103:3-9.

110. Kim HS, Lee SS. Paenibacillus humi sp. nov., isolated from peat-soil. J Gen Appl Microbiol. 2014;60:23-7.

111. Vaz-Moreira I, Faria C, Nobre MF, Schumann P, Nunes OC, Manaia CM. Paenibacillus humicus sp. nov., isolated from poultry litter compost. Int J Syst Evol Microbiol. 2007;57:2267-71.

112. Tsumori H, Shimamura A, Sakurai Y, Yamakami K. Substrate specificity of mutanase of Paenibacillus humicus from fermented food. J Health Sci. 2011;57:78-81.

113. Menezes G, Aruna K. Optimal production of exopolysaccharide by Paenibacillus Hunanensis strain Y-22 isolated from soil. Asian J Microbiol Biotechnol Environ Sci. 2014;16:189-99.

114. Liu Y, Liu L, Qiu F, Schumann P, Shi Y, Zou Y, et al. Paenibacillus hunanensis sp. nov., isolated from rice seeds. Int J Syst Evol Microbiol. 2010;60:1266-70.

115. Togo AH, Khelaifia S, Lagier JC, Caputo A, Robert C, Fournier PE, et al. Noncontiguous finished genome sequence and description of Paenibacillus ihumii sp. nov. strain AT5. New Microbes New Infect. 2016;10:142-50.

116. Lucena-Padrós H, Caballero-Guerrero B, Maldonado-Barragán A, Ruiz-Barba JL. Microbial diversity and dynamics of Spanish-style green table-olive fermentations in large manufacturing companies through culture-dependent techniques. Food Microbiol. 2014;42:154-65.

117. Cho SJ, Cho SH, Kim TS, Park SH, Kim SB, Lee GH. Paenibacillus insulae sp. nov., isolated from soil. J Microbiol. 2015;53:588-91.

118. Jin HJ, Tu R, Xu F, Chen SF. Identification of nitrogen-fixing Paenibacillus from different plant rhizospheres and a novel nifH gene detected in the P. stellifer. Microbiology. 2011;80:117-24.

119. Aguilera M, Quesada MT, del Águila VG, Morillo JA, Rivadeneyra MA, Ramos-Cormenzana A, et al. Characterisation of Paenibacillus jamilae strains that produce exopolysaccharide during growth on and detoxification of olive mill wastewaters. Bioresour Technol. 2008;99:5640-4.

120. Priya NG, Ojha A, Kajla MK, Raj A, Rajagopal R. Host plant induced variation in gut bacteria of Helicoverpa armigera. PLoS One. 2012;7:1.

121. Jin HJ, Zhou YG, Liu HC, Chen SF. Paenibacillus jilunlii sp. nov., a nitrogen-fixing species isolated from the rhizosphere of Begonia semperflorens. Int J Syst Evol Microbiol. 2011;61:1350-5.

122. Takeda M, Kamagata Y, Shinmaru S, Nishiyama T, Koizumi JI. Paenibacillus koleovorans sp. nov., able to grow on the sheath of Sphaerotilus natans. Int J Syst Evol Microbiol. 2002;52:1597-601.

123. Ko KS, Kim YS, Lee MY, Shin SY, Jung DS, Peck KR, et al. Paenibacillus konsidensis sp. nov., isolated from a patient. Int J Syst Evol Microbiol. 2008;58:2164-8.

124. Chung Y, Kim C, Hwang I, Chun J. Paenibacillus koreensis sp. nov., a new species that produces an iturin-like antifungal compound. Int J Syst Evol Microbiol. 2000;50:1495-500.

125. Xu SJ, Hong SJ, Choi W, Kim BS. Antifungal activity of Paenibacillus kribbensis strain T-9 isolated from soils against several plant pathogenic fungi. Plant Pathol J. 2014;30:102-8.

126. Yoon JH, Oh HM, Yoon BD, Kang KH, Park YH. Paenibacillus kribbensis sp. nov. and Paenibacillus terrae sp. nov., bioflocculants for efficient harvesting of algal cells. Int J Syst Evol Microbiol. 2003;53:295-301.

127. Siddiqi MZ, Siddiqi MH, Im WT, Kim YJ, Yang DC. Paenibacillus kyungheensis sp. Nov., isolated from flowers of magnolia. Int J Syst Evol Microbiol. 2015;65:3959-64.

128. Scheldeman P, Goossens K, Rodriguez-Diaz M, Pil A, Goris J, Herman L, et al. Paenibacillus lactis sp. nov., isolated from raw and heat-treated milk. Int J Syst Evol Microbiol. 2004;54:885-91.

129. Hernández-Lõpez J, Crockett S, Kunert O, Hammer E, Schuehly W, Bauer R, et al. In vitro growth inhibition by Hypericum extracts and isolated pure compounds of Paenibacillus larvae, a lethal disease affecting honeybees worldwide. Chem Biodiversity. 2014;11:695-708.

130. Widderich N, Hop̈pner A, Pittelkow M, Heider J, Smits SHJ, Bremer E. Biochemical properties of ectoine hydroxylases from extremophiles and their wider taxonomic distribution among microorganisms. PLoS One. 2014;9:4.

131. Das R, Kazy SK. Microbial diversity, community composition and metabolic potential in hydrocarbon contaminated oily sludge: Prospects for in situ bioremediation. Environ Sci Pollut Res. 2014;21:7369-89.

132. Kittiwongwattana C, Thawai C. Paenibacillus lemnae sp. nov., an endophytic bacterium of duckweed (Lemna aequinoctialis). Int J Syst Evol Microbiol. 2015;65:107-12.

133. Sá ALB, Dias ACF, Quecine MC, Cotta SR, Fasanella CC, Andreote FD, et al. Screening of endoglucanase-producing bacteria in the saline rhizosphere of Rhizophora mangle. Braz J Microbiol. 2014;45:193-7.

134. Chaudhry V, Chauhan PS, Mishra A, Goel R, Asif MH, Mantri SS, et al. Insights from the draft genome of Paenibacillus lentimorbus NRRL B-30488, a promising plant growth promoting bacterium. J Biotechnol. 2013;168:737-8.

135. Yokoyama T, Tanaka M, Fujiie A, Hasegawa M. A new strain of Paenibacillus lentimorbus isolated from larvae of the oriental beetle, Blitopertha orientalis (Coleoptera: Scarabaeidae), in Chiba Prefecture, Japan. Appl Entomol Zool. 2003;38:523-8.

136. Li YF, Calley JN, Ebert PJ, Helmes EB. Paenibacillus lentus sp. nov., a β-mannanolytic bacterium isolated from mixed soil samples in a selective enrichment using guar gum as the sole carbon source. Int J Syst Evol Microbiol. 2014;64:1166-72.

137. Carro L, Flores-Félix JD, Ramírez-Bahena MH, García-Fraile P, Martínez-Hidalgo P, Igual JM, et al. Paenibacillus lupini sp. nov., Isolated from nodules of Lupinus albus. Int J Syst Evol Microbiol. 2014;64:3028-33.

138. Tiwari S, Singh SN, Garg SK. Induced phytoremediation of metals from fly ash mediated by plant growth promoting rhizobacteria. J Environ Biol. 2013;34:717-27.

139. Hoshino T, Nakabayashi T, Hirota K, Matsuno T, Koiwa R, Fujiu S, et al. Paenibacillus macquariensis subsp. defensor subsp. nov., isolated from boreal soil. Int J Syst Evol Microbiol. 2009;59:2074-9.

140. Sharma M, Kumar A. Optimization of xylanase secretion from Paenibacillus macquariensis. Curr Trends Biotechnol Pharm. 2012;6:190-5.

141. Guo GN, Zhou X, Chen ZL, Yang ZW, Li XD, Li YH. Paenibacillus marchantiophytorum sp. nov., isolated from the liverwort herbertus sendtneri. Int J Syst Evol Microbiol. 2016;66:755-61.

142. Lee HW, Roh SW, Yim KJ, Shin NR, Lee J, Whon TW, et al. Paenibacillus marinisediminis sp. nov., a bacterium isolated from marine sediment. J Microbiol. 2013;51:312-7.

143. Bouraoui H, Rebib H, Aissa MB, Touzel JP, O'Donohue M, Manai M. Paenibacillus marinum sp. nov., a thermophilic xylanolytic bacterium isolated from a marine hot spring in Tunisia. J Basic Microbiol. 2013;53:877-83.

144. Roux V, Raoult D. Paenibacillus massiliensis sp. nov. Paenibacillus sanguinis sp. nov. and Paenibacillus timonensis sp. nov., isolated from blood cultures. Int J Syst Evol Microbiol. 2004;54:1049-54.

145. Lai WA, Asif A, Lin SY, Hung MH, Hsu YH, Liu YC, et al. Paenibacillus medicaginis sp. Nov. a chitinolytic endophyte isolated from a root nodule of alfalfa (medicago sativa l.). Int J Syst Evol Microbiol. 2015;65:3853-60.

146. Šmerda J, Sedláček I, Páčová Z, Durnová E, Smíšková A, Havel L. Paenibacillus mendelii sp. nov., from surface-sterilized seeds of Pisum sativum L. Int J Syst Evol Microbiol. 2005;55:2351-4.

147. Khianngam S, Tanasupawat S, Lee J-S, Lee KC, Akaracharanya A. Paenibacillus siamensis sp. nov., Paenibacillus septentrionalis sp. nov. and Paenibacillus montaniterrae sp. nov., xylanase-producing bacteria from Thai soils. Int J Syst Evol Microbiol. 2009;59:130-4.

148. Arora A, Krishna P, Malik V, Reddy MS. Alkalistable xylanase production by alkalitolerant Paenibacillus montaniterrae RMV1 isolated from red mud. J Basic Microbiol. 2014;54:1023-9.

149. Iida KI, Ueda Y, Kawamura Y, Ezaki T, Takade A, Yoshida SI, et al. Paenibacillus motobuensis sp. nov., isolated from a composting machine utilizing soil from Motobu-town, Okinawa, Japan. Int J Syst Evol Microbiol. 2005;55:1811-6.

150. Tang J, Qi S, Li Z, An Q, Xie M, Yang B, et al. Production, purification and application of polysaccharide-based bioflocculant by Paenibacillus mucilaginosus. Carbohydr Polym. 2014;113:463-70.

151. Khianngam S, Akaracharanya A, Tanasupawat S, Lee KC, Lee JS. Paenibacillus thailandensis sp. nov. and Paenibacillus nanensis sp. nov., xylanase-producing bacteria isolated from soil. Int J Syst Evol Microbiol. 2009;59:564-8.

152. Daane LL, Harjono I, Barns SM, Launen LA, Palleroni NJ, Haggblom MM. PAH-degradation by Paenibacillus spp. and description of Paenibacillus naphthalenovorans sp. nov., a naphthalene-degrading bacterium from the rhizosphere of salt marsh plants. Int J Syst Evol Microbiol. 2002;52:131-9.

153. Wang XM, Ma S, Yang SY, Peng R, Zheng Y, Yang H. Paenibacillus nasutitermitis sp. nov., isolated from a termite gut. Int J Syst Evol Microbiol. 2016;66:901-5.

154. Enright MR, McInerney JO, Griffin CT. Characterization of endospore-forming bacteria associated with entomopathogenic nematodes, Heterorhabditis spp., and description of Paenibacillus nematophilus sp. nov. Int J Syst Evol Microbiol. 2003;53:435-41.

155. Li QQ, Zhou XK, Dang LZ, Cheng J, Hozzein WN, Liu MJ, et al. Paenibacillus nicotianae sp. nov., isolated from a tobacco sample. Antonie Van Leeuwenhoek Int J Gen Mol Microbiol. 2014;106:1199-205.

156. Lee JN, Shin NR, Jung MJ, Roh SW, Kim MS, Lee JS, et al. Paenibacillus oceanisediminis sp. nov. isolated from marine sediment. Int J Syst Evol Microbiol. 2013;63:428-34.

157. Ten LN, Baek SH, Im WT, Lee M, Oh HW, Lee ST. Paenibacillus panacisoli sp. nov., a xylanolytic bacterium isolated from soil in a ginseng field in South Korea. Int J Syst Evol Microbiol. 2006;56:2677-81.

158. Nguyen NL, Kim YJ, Hoang VA, Kang JP, Singh P, Yang DC. Paenibacillus panaciterrae sp. Nov., isolated from ginseng-cultivated soil. Int J Syst Evol Microbiol. 2015;65:4080-6.

159. Park DS, Jeong WJ, Lee KH, Oh HW, Kim BC, Bae KS, et al. Paenibacillus pectinilyticus sp. nov., isolated from the gut of Diestrammena apicalis. Int J Syst Evol Microbiol. 2009;59:1342-7.

160. Menéndez E, Ramírez-Bahena MH, Carro L, Fernández-Pascua M, Klenk HP, Velázquez E, et al. Paenibacillus periandrae sp. nov., isolated from nodules of Periandra mediterranea. Int J Syst Evol Microbiol. 2016;66:1838-43.

161. Benardini JN, Vaishampayan PA, Schwendner P, Swanner E, Fukui Y, Osman S, et al. Paenibacillus phoenicis sp. nov., isolated from the Phoenix Lander assembly facility and a subsurface molybdenum mine. Int J Syst Evol Microbiol. 2011;61:1338-43.

162. Rivas R, Mateos PF, Martínez-Molina E, Velázquez E. Paenibacillus phyllosphaerae sp. nov., a xylanolytic bacterium isolated from the phyllosphere of Phoenix dactylifera. Int J Syst Evol Microbiol. 2005;55:743-6.

163. Zhou X, Guo GN, Wang LQ, Bai SL, Li CL, Yu R, et al. Paenibacillus physcomitrellae sp. Nov., isolated from the moss physcomitrella patens. Int J Syst Evol Microbiol. 2015;65:3400-6.

164. Moon JS, Kim JS. Isolation of Paenibacillus pinesoli sp. nov. from forest soil in Gyeonggi-Do, Korea. J Microbiol. 2014;52:273-7.

165. Yuki M, Oshima K, Suda W, Oshida Y, Kitamura K, Iida T, et al. Draft Genome Sequence of Paenibacillus pini JCM 16418T, Isolated from the Rhizosphere of Pine Tree. Genome Announcements. 2014;2:2.

166. Kim BC, Lee KH, Kim MN, Kim EM, Rhee MS, Kwon OY, et al. Paenibacillus pinihumi sp. nov., a cellulolytic bacterium isolated from the rhizosphere of Pinus densiflora. J Microbiol. 2009;47:530-5.

167. Baek SH, Yi TH, Lee ST, Im WT. Paenibacillus pocheonensis sp. nov., a facultative anaerobe isolated from soil of a ginseng field. Int J Syst Evol Microbiol. 2010;60:1163-7.

168. Bai X, Hu H, Chen H, Wei Q, Yang Z, Huang Q. Expression of a β-mannosidase from Paenibacillus polymyxa A-8 in Escherichia coli and characterization of the recombinant enzyme. PLoS One. 2014;9:11.

169. Rafigh SM, Yazdi AV, Vossoughi M, Safekordi AA, Ardjmand M. Optimization of culture medium and modeling of curdlan production from Paenibacillus polymyxa by RSM and ANN. Int J Biol Macromol. 2014;70:463-73.

170. Gastelum-Arellanez A, Paredes-López O, Olalde-Portugal V. Extracellular endoglucanase activity from Paenibacillus polymyxa BEb-40: production, optimization and enzymatic characterization. World J Microbiol Biotechnol. 2014;30:2953-65.

171. Xu Y, Liu Y, Yao S, Li J, Cheng C. Genome sequence of Paenibacillus polymyxa strain CICC 10580, isolated from the fruit of noni (Morinda citrifolia L.) grown in the Paracel Islands. Genome Announcements. 2014;2:4.

172. Dai JJ, Cheng JS, Liang YQ, Jiang T, Yuan YJ. Regulation of extracellular oxidoreduction potential enhanced (R,R)-2,3-butanediol production by Paenibacillus polymyxa CJX518. Bioresour Technol. 2014;167:433-40.

173. Eastman AW, Weselowski B, Nathoo N, Yuan Z. Complete genome sequence of Paenibacillus polymyxa CR1, a plant growth-promoting bacterium isolated from the corn rhizosphere exhibiting potential for biocontrol, biomass degradation, and biofuel production. Genome Announcements. 2014;2:1.

174. Khan Z, Kim SG, Jeon YH, Khan HU, Son SH, Kim YH. A plant growth promoting rhizobacterium, Paenibacillus polymyxa strain GBR-1, suppresses root-knot nematode. Bioresour Technol. 2008;99:3016-23.

175. Zhang CZ, Zhang WJ, Xu J. Isolation and identification of methanethiol-utilizing bacterium CZ05 and its application in bio-trickling filter of biogas. Bioresour Technol. 2013;150:338-43.

176. Phi QT, Park YM, Seul KJ, Ryu CM, Park SH, Kim JG, et al. Assessment of root-associated paenibacillus polymyxa groups on growth promotion and induced systemic resistance in pepper. J Microbiol Biotechnol. 2010;20:1605-13.

177. Lee B, Farag MA, Park HB, Kloepper JW, Lee SH, Ryu C-M. Induced Resistance by a Long-Chain Bacterial Volatile: Elicitation of Plant Systemic Defense by a C13 Volatile Produced by Paenibacillus polymyxa. PLoS One. 2012;7:11.

178. Xu S, Bai Z, Jin B, Xiao R, Zhuang G. Bioconversion of wastewater from sweet potato starch production to Paenibacillus polymyxa biofertilizer for tea plants. Sci Rep. 2014; doi:10.1038/srep04131.

179. Naghmouchi K, Hammami R, Fliss I, Teather R, Baah J, Drider D. Colistin A and colistin B among inhibitory substances of Paenibacillus polymyxa JB05-01-1. Arch Microbiol. 2012;194:363-70.

180. Yang S, Kim S, Ryu JH, Kim H. Inhibitory activity of Paenibacillus polymyxa on the biofilm formation of Cronobacter spp. on stainless steel surfaces. J Food Sci. 2013;78:M1036-M40.

181. Schmidt R, Köberl M, Mostafa A, Ramadan EM, Monschein M, Jensen KB, et al. Effects of bacterial inoculants on the indigenous microbiome and secondary metabolites of chamomile plants. Front Microbiol. 2014;5:64.

182. Stern NJ, Svetoch EA, Eruslanov BV, Kovalev YN, Volodina LI, Perelygin VV, et al. Paenibacillus polymyxa purified bacteriocin to control Campylobacter jejuni in chickens. J Food Prot. 2005;68:1450-3.

183. Anand R, Grayston S, Chanway C. N2-Fixation and Seedling Growth Promotion of Lodgepole Pine by Endophytic Paenibacillus polymyxa. Microb Ecol. 2013;66:369-74.

184. Xu SJ, Kim BS. Biocontrol of fusarium crown and root rot and promotion of growth of tomato by Paenibacillus strains isolated from soil. Mycobiology. 2014;42:158-66.

185. Mingchao M, Wang C, Ding Y, Li L, Shen D, Jiang X, et al. Complete genome sequence of Paenibacillus polymyxa SC2, a strain of plant growth-promoting rhizobacterium with broad-spectrum antimicrobial activity. J Bacteriol. 2011;193:311-2.

186. Li S, Zhang R, Wang Y, Zhang N, Shao J, Qiu M, et al. Promoter analysis and transcription regulation of fus gene cluster responsible for fusaricidin synthesis of Paenibacillus polymyxa SQR-21. Appl Microbiol Biotechnol. 2013;97:9479-89.

187. Wang S, Yang Y, Yang R, Zhang J, Chen M, Matsukawa S, et al. Cloning and Characterization of a Cold-Adapted Endo-1,5-α-l-arabinanase from Paenibacillus polymyxa and Rational Design for Acidic Applicability. J Agric Food Chem. 2014;62:8460-9.

188. Nasu Y, Nosaka Y, Otsuka Y, Tsuruga T, Nakajima M, Watanabe Y, et al. A case of Paenibacillus polymyxa bacteremia in a patient with cerebral infarction. Kansenshogaku zasshi The Journal of the Japanese Association for Infectious Diseases. 2003;77:844-8.

189. Fraimow H, Knob C, Herrero IA, Patel R. Putative VanRS-like two-component regulatory system associated with the inducible glycopeptide resistance cluster of Paenibacillus popilliae. Antimicrob Agents Chemother. 2005;49:2625-33.

190. Han TY, Tong XM, Wang YW, Wang HM, Chen XR, Kong DL, et al. Paenibacillus populi sp. nov., a novel bacterium isolated from the rhizosphere of Populus alba. Antonie Van Leeuwenhoek Int J Gen Mol Microbiol. 2015;108:659-66.

191. Romanenko LA, Tanaka N, Svetashev VI, Kalinovskaya NI. Paenibacillus profundus sp. nov., a deep sediment bacterium that produces isocoumarin and peptide antibiotics. Arch Microbiol. 2013;195:247-54.

192. Kalinovskay NI, Romanenko LA, Kalinovsky AI, Dmitrenok PS, Dyshlovoy SA. A new antimicrobial and anticancer peptide producing by the marine deep sediment strain "paenibacillus profundus" sp. nov. Sl 79. Nat Prod Commun. 2013;8:381-4.

193. Valverde A, Fterich A, Mahdhi M, Ramírez-Bahena MH, Caviedes MA, Mars M, et al. Paenibacillus prosopidis sp. nov., isolated from the nodules of Prosopis farcta. Int J Syst Evol Microbiol. 2010;60:2182-6.

194. Roux V, Fenner L, Raoult D. Paenibacillus provencensis sp. nov., isolated from human cerebrospinal fluid, and Paenibacillus urinalis sp. nov., isolated from human urine. Int J Syst Evol Microbiol. 2008;58:682-7.

195. Galmés-Truyols A, Giménez-Duran J, Bosch-Isabel C, Nicolau-Riutort A, Vanrell-Berga J, Portell-Arbona M, et al. An outbreak of cutaneous infection due to Mycobacterium abscessus associated to mesotherapy. Enferm Infecc Microbiol Clin. 2011;29:510-4.

196. Kim BC, Jeong WJ, Kim DY, Oh HW, Kim H, Park DS, et al. Paenibacillus pueri sp. nov., isolated from Pu'er tea. Int J Syst Evol Microbiol. 2009;59:1002-6.

197. Traiwan J, Park MH, Kim W. Paenibacillus puldeungensis sp. nov., isolated from a grassy sandbank. Int J Syst Evol Microbiol. 2011;61:670-3.

198. Behrendt U, Schumann P, Stieglmeier M, Pukall R, Augustin J, Spröer C, et al. Characterization of heterotrophic nitrifying bacteria with respiratory ammonification and denitrification activity - Description of Paenibacillus uliginis sp. nov., an inhabitant of fen peat soil and Paenibacillus purispatii sp. nov., isolated from a spacecraft assembly clean room. Syst Appl Microbiol. 2010;33:328-36.

199. Chen L, Wang L, Sheng XF. Paenibacillus qingshengii sp. nov., isolated from a lead–zinc tailing. Int J Syst Evol Microbiol. 2015;65:2161-6.

200. Wang DS, Jiang YY, Wei XM, Lai HX, Xue QH. Paenibacillus quercus sp. nov., isolated from rhizosphere of Quercus aliena var. acuteserrata. Antonie Van Leeuwenhoek Int J Gen Mol Microbiol. 2014;105:1173-8.

201. Gao JL, Yuan M, Wang XM, Qiu TL, Lv FY, Yang MM, et al. Paenibacillus radicis sp. Nov., an endophytic bacterium isolated from maize root. Int J Syst Evol Microbiol. 2016;66:807-11.

202. Shimoyama T, Johari NB, Tsuruya A, Nair A, Nakayama T. Paenibacillus relictisesami sp. nov., isolated from sesame oil cake. Int J Syst Evol Microbiol. 2014;64:1534-9.

203. Vaz-Moreira I, Figueira V, Lopes AR, Pukall R, Spröer C, Schumann P, et al. Paenibacillus residui sp. nov., isolated from urban waste compost. Int J Syst Evol Microbiol. 2010;60:2415-9.

204. Zhang L, Gao JS, Zhang S, Sheirdil RA, Wang XC, Zhang XX. Paenibacillus rhizoryzae sp. nov., isolated from rice rhizosphere. Int J Syst Evol Microbiol. 2015;65:3053-9.

205. Rivas R, Gutiérrez C, Abril A, Mateos PF, Martínez-Molina E, Ventosa A, et al. Paenibacillus rhizosphaerae sp. nov., isolated from the rhizosphere of Cicer arietinum. Int J Syst Evol Microbiol. 2005;55:1305-9.

206. Baik KS, Lim CH, Choe HN, Kim EM, Seong CN. Paenibacillus rigui sp. nov., isolated from a freshwater wetland. Int J Syst Evol Microbiol. 2011;61:529-34.

207. Beneduzi A, Costa PB, Parma M, Melo IS, Bodanese-Zanettini MH, Passaglia LMP. Paenibacillus riograndensis sp. nov., a nitrogen-fixing species isolated from the rhizosphere of Triticum aestivum. Int J Syst Evol Microbiol. 2010;60:128-33.

208. Sun Y, Guo Z, Zhao Q, Gao Q, Xie Q, Yang R, et al. Paenibacillus ripae sp. nov., isolated from bank side soil. Int J Syst Evol Microbiol. 2015;65:4757-62.

209. Li X, Deng Z, Liu Z, Yan Y, Wang T, Xie J, et al. The genome of Paenibacillus sabinae T27 provides insight into evolution, organization and functional elucidation of nif and nif-like genes. BMC Genomics. 2014;15:723.

210. Moon JC, Jung YJ, Jung JH, Jung HS, Cheong YR, Jeon CO, et al. Paenibacillus sacheonensis sp. nov., a xylanolytic and cellulolytic bacterium isolated from tidal flat sediment. Int J Syst Evol Microbiol. 2011;61:2753-7.

211. Wang L, Baek SH, Cui Y, Lee HG, Lee ST. Paenibacillus sediminis sp. nov., a xylanolytic bacterium isolated from a tidal flat. Int J Syst Evol Microbiol. 2012;62:1284-8.

212. Xiang WW, Wang GJ, Wang Yt, Yao R, Zhang FJ, Wang R, et al. Paenibacillus selenii sp. nov., isolated from selenium mineral soil. Int J Syst Evol Microbiol. 2014;64:2662-7.

213. Yao R, Wang R, Wang D, Su J, Zheng SX, Wang GJ. Paenibacillus selenitireducens sp. nov., a selenite-reducing bacterium isolated from a selenium mineral soil. Int J Syst Evol Microbiol. 2014;64:805-11.

214. Mishra AK, Lagier JC, Rivet R, Raoult D, Fournier PE. Non-contiguous finished genome sequence and description of Paenibacillus senegalensis sp. nov. Stand Genomic Sci. 2012;7:70-81.

215. Šmerda J, Sedláček I, Páčová Z, Krejčí E, Havel L. Paenibacillus sepulcri sp. nov., isolated from biodeteriorated mural paintings in the Servilia tomb. Int J Syst Evol Microbiol. 2006;56:2341-4.

216. Jiang B, Zhao X, Liu J, Fu L, Yang C, Hu X. Paenibacillus shenyangensis sp. nov., a bioflocculant-producing species isolated from soil under a peach tree. Int J Syst Evol Microbiol. 2015;65:220-4.

217. Tonouchi A, Tazawa D, Fujita T. Paenibacillus shirakamiensis sp. nov., isolated from the trunk surface of a japanese oak (quercus crispula). Int J Syst Evol Microbiol. 2014;64:1763-9.

218. Park MJ, Kim HB, An DS, Yang HC, Oh ST, Chung HJ, et al. Paenibacillus soli sp. nov., a xylanolytic bacterium isolated from soil. Int J Syst Evol Microbiol. 2007;57:146-50.

219. Hong YY, Ma YC, Zhou YG, Gao F, Liu HC, Chen SF. Paenibacillus sonchi sp. nov., a nitrogen-fixing species isolated from the rhizosphere of Sonchus oleraceus. Int J Syst Evol Microbiol. 2009;59:2656-61.

220. Jin HJ, Lv J, Chen SF. Paenibacillus sophorae sp. nov., a nitrogen-fixing species isolated from the rhizosphere of Sophora japonica. Int J Syst Evol Microbiol. 2011;61:767-71.

221. Kim KK, Lee KC, Yu H, Ryoo S, Park Y, Lee JS. Paenibacillus sputi sp. nov., isolated from the sputum of a patient with pulmonary disease. Int J Syst Evol Microbiol. 2010;60:2371-6.

222. Suominen I, Spröer C, Kämpfer P, Rainey FA, Lounatmaa K, Salkinoja-Salonen M. Paenibacillus stellifer sp. nov., a cyclodextrin-producing species isolated from paperboard. Int J Syst Evol Microbiol. 2003;53:1369-74.

223. Guo XQ, Gu JY, Yu YJ, Zhang WB, He LY, Sheng XF. Paenibacillus susongensis sp. nov., a mineral-weathering bacterium. Int J Syst Evol Microbiol. 2014;64:3958-63.

224. Lee JJ, Yang DH, Ko YS, Park JK, Im EY, Kim JY, et al. Paenibacillus swuensis sp. nov., a bacterium isolated from soil. J Microbiol. 2014;52:106-10.

225. Shagol CC, Krishnamoorthy R, Kim K, Sundaram S, Sa T. Arsenic-tolerant plant-growth-promoting bacteria isolated from arsenic-polluted soils in South Korea. Environ Sci Pollut Res. 2014;21:9356-65.

226. Lee FL, Tien CJ, Tai CJ, Wang LT, Liu YC, Chern LL. Paenibacillus taichungensis sp. nov., from soil in Taiwan. Int J Syst Evol Microbiol. 2008;58:2640-5.

227. Wu YF, Wu QL, Liu SJ. Paenibacillus taihuensis sp. nov., isolated from an eutrophic lake. Int J Syst Evol Microbiol. 2013;63:3652-8.

228. Lee F-L, Kuo H-P, Tai C-J, Yokota A, Lo C-C. Paenibacillus taiwanensis sp. nov., isolated from soil in Taiwan. Int J Syst Evol Microbiol. 2007;57:1351-4.

229. Xie JB, Zhang LH, Zhou YG, Liu HC, Chen SF. Paenibacillus taohuashanense sp. nov., a nitrogen-fixing species isolated from rhizosphere soil of the root of Caragana kansuensis Pojark. Antonie Van Leeuwenhoek Int J Gen Mol Microbiol. 2012;102:735-41.

230. Wang MX, Yang M, Zhou GL, Luo XS, Zhang L, Tang Y, et al. Paenibacillus tarimensis sp. nov., isolated from sand in Xinjiang, China. Int J Syst Evol Microbiol. 2008;58:2081-5.

231. Raddadi N, Cherif A, Daffonchio D, Fava F. Halo-alkalitolerant and thermostable cellulases with improved tolerance to ionic liquids and organic solvents from Paenibacillus tarimensis isolated from the Chott El Fejej, Sahara desert, Tunisia. Bioresour Technol. 2013;150:121-8.

232. Lee JC, Kim CJ, Yoon KH. Paenibacillus telluris sp. nov., a novel phosphate-solubilizing bacterium isolated from soil. J Microbiol. 2011;49:617-21.

233. Liang YL, Zhang Z, Wu M, Wu Y, Feng JX. Isolation, screening, and identification of cellulolytic bacteria from natural reserves in the subtropical region of China and optimization of cellulase production by Paenibacillus terrae ME27-1. BioMed Res Int. 2014; doi:10.1155/2014/512497.

234. Huang Z, Dai W, Zhou Z, Wang G, Lin G, Yan X, et al. Paenibacillus terreus sp. nov., isolated from forest soil. Int J Syst Evol Microbiol. 2016;66:243-7.

235. Xie CH, Yokota A. Paenibacillus terrigena sp. nov., isolated from soil. Int J Syst Evol Microbiol. 2007;57:70-2.

236. Rai SK, Roy JK, Mukherjee AK. Characterisation of a detergent-stable alkaline protease from a novel thermophilic strain Paenibacillus tezpurensis sp. nov. AS-S24-II. Appl Microbiol Biotechnol. 2010;85:1437-50.

237. Ueda J, Kurosawa N. Characterization of an extracellular thermophilic chitinase from Paenibacillus thermoaerophilus strain TC22-2b isolated from compost. World J Microbiol Biotechnol. 2015;31:135-43.

238. Zhou Y, Gao S, Wei DQ, Yang LL, Huang X, He J, et al. Paenibacillus thermophilus sp. nov., a novel bacterium isolated from a sediment of hot spring in Fujian province, China. Antonie Van Leeuwenhoek Int J Gen Mol Microbiol. 2012;102:601-9.

239. Huang E, Yousef AE. Paenibacterin, a novel broad-spectrum lipopeptide antibiotic, neutralises endotoxins and promotes survival in a murine model of Pseudomonas aeruginosa-induced sepsis. Int J Antimicro Agents. 2014;44:74-7.

240. Ouyang J, Pei Z, Lutwick L, Dalai S, Yang L, Cassai N, et al. Case report: Paenibacillus thiaminolyticus: A new cause of human infection, inducing bacteremia in a patient on hemodialysis. Ann Clin Lab Sci. 2008;38:393-400.

241. Kong BH, Liu QF, Liu M, Liu Y, Liu L, Li CL, et al. Paenibacillus typhae sp. nov., isolated from roots of Typha angustifolia L. Int J Syst Evol Microbiol. 2013;63:1037-44.

242. Han LL, He JZ, Zheng YM, Zeng J, Zhang LM. Paenibacillus tibetensis sp. nov., a psychrophilic bacterium isolated from alpine swamp meadow soil. Int J Syst Evol Microbiol. 2015;65:1583-6.

243. Nelson DM, Glawe AJ, Labeda DP, Cann IKO, Mackie RI. Paenibacillus tundrae sp. nov. and Paenibacillus xylanexedens sp. nov., psychrotolerant, xylan-degrading bacteria from Alaskan tundra. Int J Syst Evol Microbiol. 2009;59:1708-14.

244. Bosshard PP, Zbinden R, Altwegg M. Paenibacillus turicencis sp. nov., a novel bacterium harbouring heterogeneities between 16S rRna genes. Int J Syst Evol Microbiol. 2002;52:2241-9.

245. Kuisiene N, Raugalas J, Spröer C, Kroppenstedt RM, Stuknyte M, Chitavichius D. Paenibacillus tylopili sp.nov., a chitinolytic bacterium isolated from the mycorhizosphere of Tylopilus felleus. Folia Microbiol (Praha). 2008;53:433-7.

246. Mah JH, Chang YH, Hwang HJ. Paenibacillus tyraminigenes sp. nov. isolated from Myeolchi-jeotgal, a traditional Korean salted and fermented anchovy. Int J Food Microbiol. 2008;127:209-14.

247. Derikvand P, Etemadifar Z, Saber H. Sulfur removal from dibenzothiophene by newly isolated paenibacillus validus strain PD2 and process optimization in aqueous and biphasic (Model-Oil) systems. Pol J Microbiol. 2015;64:47-54.

248. Rawat M, Rai JPN. Adsorption of heavy metals by Paenibacillus validus strain MP5 isolated from industrial effluent-polluted soil. Biorem J. 2012;16:66-73.

249. Pepi M, Agnorelli C, Bargagli R. Iron demand by thermophilic and mesophilic bacteria isolated from an antarctic geothermal soil. Biometals. 2005;18:529-36.

250. Chen XR, Shao CB, Wang YW, He MX, Ma KD, Wang HM, et al. Paenibacillus vini sp. nov., isolated from alcohol fermentation pit mud in Sichuan Province, China. Antonie Van Leeuwenhoek Int J Gen Mol Microbiol. 2015;107:1429-36.

251. Ingham CJ, Jacob EB. Swarming and complex pattern formation in Paenibacillus vortex studied by imaging and tracking cells. BMC Microbiol. 2008;8:36.

252. Glaeser SP, Falsen E, Busse HJ, Kämpfer P. Paenibacillus vulneris sp. nov., isolated from a necrotic wound. Int J Syst Evol Microbiol. 2013;63:777-82.

253. Gao JL, Lv FY, Wang XM, Qiu TL, Yuan M, Li JW, et al. Paenibacillus wenxiniae sp. nov., a nifH gene -harbouring endophytic bacterium isolated from maize. Antonie Van Leeuwenhoek Int J Gen Mol Microbiol. 2015;108:1015-22.

254. Baik KS, Choe HN, Park SC, Kim EM, Seong CN. Paenibacillus wooponensis sp. nov., isolated from wetland freshwater. Int J Syst Evol Microbiol. 2011;61:2763-8.

255. Lee JC, Yoon KH. Paenibacillus woosongensis sp. nov., a xylanolytic bacterium isolated from forest soil. Int J Syst Evol Microbiol. 2008;58:612-6.

256. Paul T, Halder SK, Das A, Bera S, Maity C, Mandal A, et al. Exploitation of chicken feather waste as a plant growth promoting agent using keratinase producing novel isolate Paenibacillus woosongensis TKB2. Biocatal Agric Biotechnol. 2013;2:50-7.

257. Rodríguez-Díaz M, Lebbe L, Rodelas B, Heyrman J, De Vos P, Logan NA. Paenibacillus wynnii sp. nov., a novel species harbouring the nifH gene, isolated from Alexander Island, Antarctica. Int J Syst Evol Microbiol. 2005;55:2093-9.

258. Kim DU, Kim SG, Lee H, Chun J, Cho JC, Ka JO. Paenibacillus xanthinilyticus sp. nov., isolated from agricultural soil. Int J Syst Evol Microbiol. 2015;65:2937-42.

259. Lim JM, Jeon CO, Park DJ, Xu LH, Jiang CL, Kim CJ. Paenibacillus xinjiangensis sp. nov., isolated from Xinjiang province in china. Int J Syst Evol Microbiol. 2006;56:2579-82.

260. Tachaapaikoon C, Tanasupawat S, Pason P, Sornyotha S, Waeonukul R, Kyu KL, et al. Paenibacillus xylaniclasticus sp. nov., a xylanolytic-cellulolytic bacterium isolated from sludge in an anaerobic digester. J Microbiol. 2012;50:394-400.

261. Khianngam S, Tanasupawat S, Akaracharanya A, Kim KK, Lee KC, Lee JS. Paenibacillus xylanisolvens sp. nov., a xylan-degrading bacterium from soil. Int J Syst Evol Microbiol. 2011;61:160-4.

262. Sukweenadhi J, Kim YJ, Lee KJ, Koh SC, Hoang VA, Nguyen NL, et al. Paenibacillus yonginensis sp. nov., a potential plant growth promoting bacterium isolated from humus soil of Yongin forest. Antonie Van Leeuwenhoek. 2014;106:935-45.

263. Niu L, Tang T, Ma Z, Song L, Zhang K, Chen Y, et al. Paenibacillus yunnanensis sp. Nov., isolated from pu’er tea. Int J Syst Evol Microbiol. 2015;65:3806-11.

264. Ma YC, Zhang J, Chen SF. Paenibacillus zanthoxyli sp. nov., a novel nitrogen-fixing species isolated from the rhizosphere of Zanthoxylum simulans. Int J Syst Evol Microbiol. 2007;57:873-7.

265. Liu Y, Zhai L, Wang R, Zhao R, Zhang X, Chen C, et al. Paenibacillus zeae sp. Nov., isolated from maize (Zea mays L.) seeds. Int J Syst Evol Microbiol. 2015;65:4533-8.
