# Supplementary material for: Current knowledge and perspectives of Paenibacillus: a review
Source: Microb Cell Fact. 2016 Dec 1;15:203. doi: 10.1186/s12934-016-0603-7 (PMC5134293; doi:10.1186/s12934-016-0603-7)
Supplement: Supplementary file 2 — Additional file 2. List of Paenibacillus genome sequencing projects and their progress. [file 12934_2016_603_MOESM2_ESM.docx]

**Additional File 2.**List of *Paenibacillus*genome sequencing projects and their progress.

| **Strain name** | **Sequencing progress** | **References** |
| --- | --- | --- |
| P. algeriensis | Complete | [1] |
| P. alginolyticus DSM 5050 | Permanent draft | JGI GOLD |
| P. alginolyticus NBRC 15375 | Incomplete | JGI GOLD |
| P. algorifonticola CGMCC 1.10223 | Incomplete | JGI GOLD |
| P. algorifonticola  P. algorifonticola XJ259 | Draft  Permanent draft | [2]  JGI GOLD |
| P. alvei A6-6i | Draft | [3] |
| P. alvei DSM 29 | Draft | [4] |
| P. alvei E194 | Permanent draft | JGI GOLD |
| P. alvei NAS6G-6 | Incomplete | JGI GOLD |
| P. alvei TS-15 | Draft | [3] |
| P. antibioticophila GD11T  P. apiaries 32O-W | Complete  Incomplete | [5]  JGI GOLD |
| P. assamensis DSM 18201 | Permanent draft | JGI GOLD |
| P. barengoltzii CC33_002B | Incomplete | JGI GOLD |
| P. barengoltzii G22 | Permanent draft | JGI GOLD |
| P. barengoltzii J12 | Incomplete | JGI GOLD |
| P. barengoltzii NBRC 101215 | Incomplete | JGI GOLD |
| P. beijingensis 7188T | Complete | [6] |
| P. beijingensis DSM 24997  P. borealis DSM 13188 | Complete  Complete | JGI GOLD  JGI GOLD |
| P. camerouensis  P. chitinolyticus NBRC 15660 | Complete  Permanent draft | [7]  JGI GOLD |
| P. chondroitinus NBRC 15376 | Incomplete | JGI GOLD |
| P. chondroitinus OK414 | Incomplete | JGI GOLD |
| P. cineris HME8285 | Targeted | JGI GOLD |
| P. curdlanolyticus YK9 | Permanent draft | JGI GOLD |
| P. daejeonensis DSM 15491 | Permanent draft | JGI GOLD |
| P. darakensis  P. darwinianus Br | Draft  Draft | [8]  [9] |
| P. darwinianus CE1 | Draft | [9] |
| P. darwinianus MB1 | Draft | [9] |
| P. dauci H9  P. dendritiformis C | Permanent Draft  Incomplete | JGI GOLD  JGI GOLD |
| P. dendritiformis C454 | Draft | [10] |
| P. durus P3L-5 | Incomplete | JGI GOLD |
| P. durus VPI 6563-1 | Complete | JGI GOLD |
| P. ehimensis A2 | Permanent draft | JGI GOLD |
| P. ehimensis NBRC 15659 | Permanent Draft | JGI GOLD |
| P. elgii B69 | Draft | [11] |
| P. elgii JPL_MER157 | Incomplete | JGI GOLD |
| P. elgii NBRC 100335 | Incomplete | JGI GOLD |
| P. fonticola DSM 21315 | Permanent draft | JGI GOLD |
| P. forsythiae T98 | Permanent Draft | JGI GOLD |
| P. ginsengihumi DSM 21568 | Permanent draft | JGI GOLD |
| P. glucanolyticus | Incomplete | JGI GOLD |
| P. glucanolyticus NBRC 15330 | Incomplete | JGI GOLD |
| P. glycanilyticus NBRC 16618 | Incomplete | JGI GOLD |
| P. gorillae G1 | Complete | [12] |
| P. graminis DSM 15220 | Complete | JGI GOLD |
| P. graminis RSA19 | Permanent draft | JGI GOLD |
| P. harenae DSM 16969 | Permanent draft | JGI GOLD |
| P. humicus NBRC 102415 | Incomplete | JGI GOLD |
| P. ihumii AT5  P. illinoisensis NBRC 15959 | Complete  Incomplete | [13]  JGI GOLD |
| P. jamilae NS115  P. jilunlii CGMCC1.10239  P. kobensis NBRC 15729 | Incomplete  Incomplete  Incomplete | JGI GOLD  JGI GOLD  JGI GOLD |
| P. koleovorans NBRC 103111 | Incomplete | JGI GOLD |
| P. lactis 154 | Permanent draft | JGI GOLD |
| P. lactis CIP 108827 | Incomplete | JGI GOLD |
| P. larvae | Draft | [14] |
| P. larvae larvae 08-100, DSM 25719 | Permanent draft | JGI GOLD |
| P. larvae larvae 4-309, DSM 25430 | Complete | JGI GOLD |
| P. larvae larvae B-3650 | Permanent draft | JGI GOLD |
| P. larvae larvae BRL-230010 | Permanent draft | JGI GOLD |
| P. larvae MEX14  P. lautus NBRC 15380 | Permanent draft  Incomplete | JGI GOLD  JGI GOLD |
| P. lautus Y4.12MC10 | Complete | [15] |
| P. lentimorbus NRRL B-30488 | Draft | [16] |
| P. macerans 8244 | Incomplete | JGI GOLD |
| P. macerans NBRC 15307 | Incomplete | JGI GOLD |
| P. massiliensis 2301065 | Permanent draft | JGI GOLD |
| P. massiliensis T7 | Permanent draft | JGI GOLD |
| P. methanoliconsumens BL17-2  P. methanolutens BL13  P. mucilaginosus 3016 | Targeted  Targeted  Complete | JGI GOLD  JGI GOLD  [17] |
| P. mucilaginosus K02 | Complete | JGI GOLD |
| P. mucilaginosus KNP414 | Complete | [18] |
| P. odorifer DSM 15391 | Complete | JGI GOLD |
| P. pabuli NBRC 13638 | Incomplete | JGI GOLD |
| P. panacisoli DSM 21345 | Permanent draft | JGI GOLD |
| P. pasadenensis DSM 19293 | Permanent draft | JGI GOLD |
| P. peoriae KCTC 3763 | Draft | [19] |
| P. phyllostachyicola  P. phyllostachyos BL16  P. pini JCM 16418 | Incomplete  Targeted  Draft | JGI GOLD  JGI GOLD  [20] |
| P. pinihumi DSM 23905 | Permanent draft | JGI GOLD |
| P. pinihumi JCM 16419 | Incomplete | JGI GOLD |
| P. polymyxa ATCC 12321 | Draft | [21] |
| P. polymyxa ATCC 842 T | Draft | [22] |
| P. polymyxa CF05 | Complete | [23] |
| P. polymyxa CICC 10580 | Draft | [24] |
| P. polymyxa CR1 | Complete | [25] |
| P. polymyxa DSM 365 | Draft | [26] |
| P. polymyxa E681 | Complete | [27] |
| P. polymyxa EBL06 | Draft | [28] |
| P. polymyxa M-1 | Draft | [29] |
| P. polymyxa NRRL B-30509 | Draft | [30] |
| P. polymyxa OSY-DF | Draft | [31] |
| P. polymyxa Sb3-1 | Complete | [32] |
| P. polymyxa SC2 | Complete | [33] |
| P. polymyxa SQR-21 | Complete | [34] |
| P. popilliae ATCC 14706T | Draft | [35] |
| P. provencensis 4401170 | Complete plasmid  Incomplete | [35]  JGI GOLD |
| P. pseudosasae BL14  P. pseudosasicola BL27  P. riograndensis SBR5T | Targeted  Targeted  Complete | JGI GOLD  JGI GOLD  [36] |
| P. sabinae T27 | Complete | [37] |
| P. sanguinis 2301083 | Permanent draft | JGI GOLD |
| P. senegalensis JC66 | Complete | [38] |
| P. shenyangensis  P. sonchi X19-5 | Draft  Permanent draft | [39]  JGI GOLD |
| P. sophorae CGMCC 1.10238 | Incomplete | JGI GOLD |
| P. sophorae S27 | Permanent draft | JGI GOLD |
| P. stellifer DSM 14472 | Complete | JGI GOLD |
| P. swuensis | Incomplete | JGI GOLD |
| P. taihuensis CGMCC1.10966  P. taiwanensis DSM 18679 | Incomplete  Permanent draft | JGI GOLD  JGI GOLD |
| P. terrae HPL-003 | Complete | [40] |
| P. terrae NRRL B-30644 | Draft | [30] |
| P. terrigena DSM 21567 | Permanent draft | JGI GOLD |
| P. thiaminolyticus NBRC 15656 | Incomplete | JGI GOLD |
| P. tianmuensis CGMCC 1.8946 | Incomplete | JGI GOLD |
| P. timonensis 2301032 | Incomplete | JGI GOLD |
| P. uliginis N3/975 | Incomplete | JGI GOLD |
| P. urinalis 5402403 | Incomplete | JGI GOLD |
| P. validus NBRC 15382 | Incomplete | JGI GOLD |
| P. vortex V453 | Complete | [41] |
| P. wynnii DSM 18334 | Incomplete | JGI GOLD |
| P. xinjiangensis CGMCC 1.10439 | Incomplete | JGI GOLD |
| P. wulumuqiensis Y24  P. zanthoxyli JH29 | Permanent draft  Permanent draft | JGI GOLD  JGI GOLD |
| P. sp. PAMC 26794 | Draft | [42] |
| P. sp. OSY-SE | Draft | [43] |
| P. sp. ICGEB2008 (MTCC 5639) | Draft | [44] |
| P. sp. TCA20 | Draft | [45] |
| P. sp. JDR-2 | Complete | [46] |
| P. sp. D14 | Permanent Draft | JGI GOLD |
| P. sp. 4_7_47FAA | Incomplete | JGI GOLD |
| P. sp. HGF5 | Permanent Draft | JGI GOLD |
| P. sp. HGF7 | Permanent Draft | JGI GOLD |
| P. sp. J6 | Incomplete | JGI GOLD |
| P. sp. J10 | Incomplete | JGI GOLD |
| P. sp. HGH0039 | Permanent draft | JGI GOLD |
| P. sp. J14 | Permanent Draft | JGI GOLD |
| P. sp. HW567 | Permanent Draft | JGI GOLD |
| P. sp. Aloe-11 | Permanent Draft | [47] |
| P. sp. WLY78 | Permanent Draft | JGI GOLD |
| P. sp. FSL R5-192 | Permanent Draft | JGI GOLD |
| P. sp. FSL H8-237 | Permanent Draft | JGI GOLD |
| P. sp. FSL R7-277 | Permanent Draft | JGI GOLD |
| P. sp. FSL R7-269 | Permanent Draft | JGI GOLD |
| P. sp. FSL R5-808 | Permanent Draft | JGI GOLD |
| P. sp. FSL H7-689 | Permanent Draft | JGI GOLD |
| P. sp. FSL H8-457 | Permanent Draft | JGI GOLD |
| P. sp. GD6 | Incomplete | JGI GOLD |
| P. sp. 3M17 | Incomplete | JGI GOLD |
| P. sp. G4 | Permanent Draft | JGI GOLD |
| P. sp. URHA0014 | Permanent Draft | JGI GOLD |
| P. sp. A9 | Permanent Draft | [48] |
| P. sp. UNC181MFCol5.1 | Incomplete | JGI GOLD |
| P. sp. 1-18 | Permanent Draft | JGI GOLD |
| P. sp. 1-49 | Permanent Draft | JGI GOLD |
| P. sp. GD11 | Permanent Draft | JGI GOLD |
| P. sp. UNC217MF | Permanent Draft | JGI GOLD |
| P. sp. UNC451MF | Permanent Draft | JGI GOLD |
| P. sp. UNC496MF | Incomplete | JGI GOLD |
| P. sp. UNC499MF | Incomplete | JGI GOLD |
| P. sp. UNC80MF | Incomplete | JGI GOLD |
| P. sp. UNCCL52 | Permanent Draft | JGI GOLD |
| P. sp. MAEPY2 | Permanent Draft | [49] |
| P. sp. MAEPY1 | Permanent Draft | [49] |
| P. sp. JCM 10914 | Permanent Draft | [50] |
| P. sp. P1XP2 | Permanent Draft | [51] |
| P. sp. UNCCL117 | Incomplete | JGI GOLD |
| P. sp. JCM 16163 | Incomplete | JGI GOLD |
| P. sp. JCM 18996 | Incomplete | JGI GOLD |
| P. sp. JCM 9795 | Incomplete | JGI GOLD |
| P. sp. JCM 9796 | Incomplete | JGI GOLD |
| P. sp. MSt1 | Permanent Draft | [52] |
| P. sp. SH7 | Incomplete | JGI GOLD |
| P. sp. CL6Col | Targeted | JGI GOLD |
| P. sp. CL141A | Targeted | JGI GOLD |
| P. sp. CL130 | Targeted | JGI GOLD |
| P. sp. CL123 | Targeted | JGI GOLD |
| P. sp. St-s | Incomplete | JGI GOLD |
| P. sp. P22 | Draft | [53] |
| P. sp. 1_12 | Incomplete | JGI GOLD |
| P. sp. 276b | Incomplete | JGI GOLD |
| P. sp. NFR01 | Incomplete | JGI GOLD |
| P. sp. FSL H7-0357 | Complete | JGI GOLD |
| P. sp. FSL H7-0737 | Complete | JGI GOLD |
| P. sp. FSL P4-0081 | Complete | JGI GOLD |
| P. sp. FSL R5-0345 | Complete | JGI GOLD |
| P. sp. FSL R5-0912 | Complete | JGI GOLD |
| P. sp. FSL R7-0273 | Complete | JGI GOLD |
| P. sp. FSL R7-0331 | Complete | JGI GOLD |
| P. sp. CF126 | Incomplete | JGI GOLD |
| P. sp. Ov031 | Incomplete | JGI GOLD |
| P. sp. YR247 | Incomplete | JGI GOLD |
| P. sp. 181MFCol5.1 | Targeted | JGI GOLD |
| P. sp. 453MF | Targeted | JGI GOLD |
| P. sp. IHBB 10380 | Complete | [54] |
| P. sp. IHB B 3415  P. sp. 5 L8  P. sp. A2  P. sp. D9  P. sp. DMB20  P. sp. VKM B-2647  P. sp. FJAT-22460  P. sp. A59 | Draft  Draft  Draft  Permanent Draft  Permanent Draft  Permanent Draft  Permanent Draft  Permanent Draft | [55]  [56]  [57]  JGI GOLD  JGI GOLD  JGI GOLD  JGI GOLD  JGI GOLD |

1. Bendjama E, Loucif L, Diene SM, Michelle C, Gacemi-Kirane D, Rolain J-M. Non-contiguous finished genome sequence and description of Paucisalibacillus algeriensis sp nov. Stand Genomic Sci. 2014;9:1352-65.

2. Zhu L, Wu Q, Xu Q, Xu X, Jiang L, Huang H. Draft genome sequence of Paenibacillus algorifonticola sp. nov., an antimicrobial-producing strain. Genomics Data. 2015;5:302-8.

3. Luo Y, Wang C, Allard S, Strain E, Allard MW, Brown EW, et al. Draft genome sequences of Paenibacillus alvei A6-6i and TS-15. Genome Announcements. 2013;1:1.

4. Djukic M, Becker D, Poehlein A, Voget S, Daniel R. Genome sequence of paenibacillus alvei DSM 29, a secondary invader during european foulbrood outbreaks. J Bacteriol. 2012;194:6365.

5. Dubourg G, Cimmino T, Senkar SA, Lagier JC, Robert C, Flaudrops C, et al. Noncontiguous finished genome sequence and description of Paenibacillus antibioticophila sp. nov. GD11T, the type strain of Paenibacillus antibioticophila. New Microbes New Infect. 2015;8:137-47.

6. Kwak Y, Shin J-H. Complete genome sequence of Paenibacillus beijingensis 7188T (=DSM 24997T), a novel rhizobacterium from jujube garden soil. J Biotechnol. 2015;206:75-6.

7. Keita MB, Padhmanabhan R, Robert C, Delaporte E, Raoult D, Fournier PE, et al. Non-contiguous-Finished Genome Sequence and Description of Paenibacillus camerounensis sp. nov. Microb Ecol. 2016;71:990-8.

8. Lo CI, Sankar SA, Fall B, Sambe-Ba B, Mediannikov O, Robert C, et al. High-quality genome sequence and description of Paenibacillus dakarensis sp. nov. New Microbes New Infect. 2016;10:132-41.

9. Dsouza M, Taylor MW, Turner SJ, Aislabie J. Genome-based comparative analyses of antarctic and temperate species of Paenibacillus. PLoS One. 2014;9:10.

10. Sirota-Madi A, Olender T, Helman Y, Brainis I, Finkelshtein A, Roth D, et al. Genome sequence of the pattern-forming social bacterium Paenibacillus dendritiformis C454 chiral morphotype. J Bacteriol. 2012;194:2127-8.

11. Ding R, Li Y, Qian C, Wu X. Draft genome sequence of Paenibacillus elgii B69, A strain with broad antimicrobial activity. J Bacteriol. 2011;193:4537.

12. Keita MB, Padhmananabhan R, Caputo A, Robert C, Delaporte E, Raoult D, et al. Non-contiguous finished genome sequence and description of Paenibacillus gorillae sp nov. Stand Genomic Sci. 2014;9:1031-45.

13. Togo AH, Khelaifia S, Lagier JC, Caputo A, Robert C, Fournier PE, et al. Noncontiguous finished genome sequence and description of Paenibacillus ihumii sp. nov. strain AT5. New Microbes New Infect. 2016;10:142-50.

14. Qin X, Evans JD, Aronstein KA, Murray KD, Weinstock GM. Genome sequences of the honey bee pathogens Paenibacillus larvae and Ascosphaera apis. Insect Mol Biol. 2006;15:715-8.

15. Mead DA, Lucas S, Copeland A, Lapidus A, Cheng JF, Bruce DC, et al. Complete genome sequence of Paenibacillus strain Y4.12MC10, a novel Paenibacillus lautus strain isolated from obsidian hot spring in yellowstone national park. Stand Genomic Sci. 2012;6:366-85.

16. Chaudhry V, Chauhan PS, Mishra A, Goel R, Asif MH, Mantri SS, et al. Insights from the draft genome of Paenibacillus lentimorbus NRRL B-30488, a promising plant growth promoting bacterium. J Biotechnol. 2013;168:737-8.

17. Ma M, Wang Z, Li L, Jiang X, Guan D, Cao F, et al. Complete genome sequence of Paenibacillus mucilaginosus 3016, a bacterium functional as microbial fertilizer. J Bacteriol. 2012;194:2777-8.

18. Lu J-J, Wang J-F, Hu X-F. Genome Sequence of Growth-Improving Paenibacillus mucilaginosus Strain KNP414. Genome Announcements. 2013;1:5.

19. Jeong HY, Choi SK, Park SY, Kim SH, Park SH. Draft genome sequence of paenibacillus peoriae strain KCTC 3763 T. J Bacteriol. 2012;194:1237-8.

20. Yuki M, Oshima K, Suda W, Oshida Y, Kitamura K, Iida T, et al. Draft Genome Sequence of Paenibacillus pini JCM 16418T, Isolated from the Rhizosphere of Pine Tree. Genome Announcements. 2014;2:2.

21. Tong YJ, Ji XJ, Liu LG, Shen MQ, Huang H. Genome Sequence of Paenibacillus polymyxa ATCC 12321, a Promising Strain for Optically Active (R,R)-2,3-Butanediol Production. Genome Announc. 2013;1:4.

22. Jeong H, Park SY, Chung WH, Kim SH, Kim N, Park SH, et al. Draft genome sequence of the Paenibacillus polymyxa type strain (ATCC 842 T), a plant growth-promoting bacterium. J Bacteriol. 2011;193:5026-7.

23. Lei M, Lu P, Jin L, Wang Y, Qin J, Xu X, et al. Complete Genome Sequence of Paenibacillus polymyxa CF05, a Strain of Plant Growth-Promoting Rhizobacterium with Elicitation of Induced Systemic Resistance. Genome Announc. 2015;3:2.

24. Xu Y, Liu Y, Yao S, Li J, Cheng C. Genome sequence of Paenibacillus polymyxa strain CICC 10580, isolated from the fruit of noni (Morinda citrifolia L.) grown in the Paracel Islands. Genome Announcements. 2014;2:4.

25. Eastman AW, Weselowski B, Nathoo N, Yuan Z. Complete genome sequence of Paenibacillus polymyxa CR1, a plant growth-promoting bacterium isolated from the corn rhizosphere exhibiting potential for biocontrol, biomass degradation, and biofuel production. Genome Announcements. 2014;2:1.

26. Xie NZ, Li JX, Song LF, Hou JF, Guo L, Du QS, et al. Genome sequence of type strain Paenibacillus polymyxa DSM 365, a highly efficient producer of optically active (R,R)-2,3-butanediol. J Biotechnol. 2015;195:72-3.

27. Kim JF, Jeong H, Park SY, Kim SB, Park YK, Choi SK, et al. Genome sequence of the polymyxin-producing plant-probiotic rhizobacterium Paenibacillus polymyxa E681. J Bacteriol. 2010;192:6103-4.

28. Liang S, Jin D, Wang X, Fan H, Bai Z. Draft Genome Sequence of Paenibacillus polymyxa EBL06, a Plant Growth-Promoting Bacterium Isolated from Wheat Phyllosphere. Genome Announc. 2015;3:3.

29. Niu B, Rueckert C, Blom J, Wang Q, Borriss R. The genome of the plant growth-promoting rhizobacterium Paenibacillus polymyxa M-1 contains nine sites dedicated to nonribosomal synthesis of lipopeptides and polyketides. J Bacteriol. 2011;193:5862-3.

30. van Belkum MJ, Lohans CT, Vederas JC. Draft Genome Sequences of Paenibacillus polymyxa NRRL B-30509 and Paenibacillus terrae NRRL B-30644, Strains from a Poultry Environment That Produce Tridecaptin A and Paenicidins. Genome Announc. 2015;3:2.

31. Huang E, Yousef AE. Draft genome sequence of Paenibacillus polymyxa OSY-DF, which coproduces a lantibiotic, paenibacillin, and polymyxin E1. J Bacteriol. 2012;194:4739-40.

32. Rybakova D, Wetzlinger U, Muller H, Berg G. Complete Genome Sequence of Paenibacillus polymyxa Strain Sb3-1, a Soilborne Bacterium with Antagonistic Activity toward Plant Pathogens. Genome Announc. 2015;3:2.

33. Mingchao M, Wang C, Ding Y, Li L, Shen D, Jiang X, et al. Complete genome sequence of Paenibacillus polymyxa SC2, a strain of plant growth-promoting rhizobacterium with broad-spectrum antimicrobial activity. J Bacteriol. 2011;193:311-2.

34. Li S, Yang D, Qiu M, Shao J, Guo R, Shen B, et al. Complete genome sequence of Paenibacillus polymyxa SQR-21, a plant growth-promoting rhizobacterium with antifungal activity and rhizosphere colonization ability. Genome Announcements. 2014;2:2.

35. Iiyama K, Mori K, Mon H, Chieda Y, Lee JM, Kusakabe T, et al. Draft genome sequence of Paenibacillus popilliae ATCC 14706T. J Insect Biotechnol Sericology. 2013;82:45-8.

36. Brito LF, Bach E, Kalinowski J, Rückert C, Wibberg D, Passaglia LM, et al. Complete genome sequence of Paenibacillus riograndensis SBR5T, a Gram-positive diazotrophic rhizobacterium. J Biotechnol. 2015;207:30-1.

37. Li X, Deng Z, Liu Z, Yan Y, Wang T, Xie J, et al. The genome of Paenibacillus sabinae T27 provides insight into evolution, organization and functional elucidation of nif and nif-like genes. BMC Genomics. 2014;15:723.

38. Mishra AK, Lagier JC, Rivet R, Raoult D, Fournier PE. Non-contiguous finished genome sequence and description of Paenibacillus senegalensis sp. nov. Stand Genomic Sci. 2012;7:70-81.

39. Fu L, Jiang B, Liu J, Zhao X, Liu Q, Hu X. Genome sequence analysis of a flocculant-producing bacterium, Paenibacillus shenyangensis. Biotechnol Lett. 2016;38:447-53.

40. Shin SH, Kim S, Kim JY, Song HY, Cho SJ, Kim DR, et al. Genome sequence of Paenibacillus terrae HPL-003, a xylanase-producing bacterium isolated from soil found in forest residue. J Bacteriol. 2012;194:1266.

41. Sirota-Madi A, Olender T, Helman Y, Ingham C, Brainis I, Roth D, et al. Genome sequence of the pattern forming Paenibacillus vortex bacterium reveals potential for thriving in complex environments. BMC Genomics. 2010;11:710.

42. Park H, Kim D. Draft genome sequence of a humic substance-degrading Paenibacillus sp. isolated from the subarctic grasslands at low temperature. Genome Announcements. 2013;1:1.

43. Huang E, Guo Y, Yousef AE. Draft genome sequence of paenibacillus sp. strain OSY-SE, a bacterium producing the novel broad-spectrum lipopeptide antibiotic paenibacterin. J Bacteriol. 2012;194:6306.

44. Adlakha N, Kushwaha HR, Rajagopal R, Yazdani SS. Draft genome sequence of the Paenibacillus sp. strain ICGEB2008 (MTCC 5639) isolated from the gut of Helicoverpa armigera. Genome announcements. 2013;1:1.

45. Fujinami S, Takeda-Yano K, Onodera T, Satoh K, Sano M, Takahashi Y, et al. Draft genome sequence of calcium-dependent Paenibacillus sp. strain TCA20, isolated from a hot spring containing a high concentration of calcium ions. Genome Announcements. 2014;2:5.

46. Chow V, Nong G, St John FJ, Rice JD, Dickstein E, Chertkov O, et al. Complete genome sequence of Paenibacillus sp. strain JDR-2. Stand Genomic Sci. 2012;6:1-10.

47. Li NZ, Xia T, Xu YL, Qiu RR, Xiang H, He D, et al. Genome sequence of Paenibacillus sp. strain Aloe-11, an endophytic bacterium with broad antimicrobial activity and intestinal colonization ability. J Bacteriol. 2012;194:2117-8.

48. Jiang BH, Liu JL, Hu XM. Draft Genome Sequence of the Efficient Bioflocculant-Producing Bacterium Paenibacillus sp. Strain A9. Genome Announc. 2013;1:2.

49. Chua P, Yoo HS, Gan HM, Lee SM. Draft Genome Sequences of Two Cellulolytic Paenibacillus sp. Strains, MAEPY1 and MAEPY2, from Malaysian Landfill Leachate. Genome Announc. 2014;2:1.

50. Ohkuma M, Yuki M, Oshima K, Suda W, Oshida Y, Kitamura K, et al. Draft genome sequence of the alkaliphilic and xylanolytic Paenibacillus sp. strain JCM 10914, isolated from the gut of a soil-feeding termite. Genome Announcements. 2014;2:1.

51. Adelskov J, Patel BK. Draft Genome Sequence of Paenibacillus Strain P1XP2, a Polysaccharide-Degrading, Thermophilic, Facultative Anaerobic Bacterium Isolated from a Commercial Bioreactor Degrading Food Waste. Genome Announc. 2015;3:1.

52. Aw YK, Ong KS, Yule CM, Gan HM, Lee SM. Draft Genome Sequence of Paenibacillus sp. Strain MSt1 with Broad Antimicrobial Activity, Isolated from Malaysian Tropical Peat Swamp Soil. Genome Announc. 2014;2:5.

53. Hanak AM, Nagler M, Weinmaier T, Sun X, Fragner L, Schwab C, et al. Draft Genome Sequence of the Growth-Promoting Endophyte Paenibacillus sp. P22, Isolated from Populus. Genome Announc. 2014;2:2.

54. Pal M, Swarnkar MK, Thakur R, Kiran S, Chhibber S, Singh AK, et al. Complete Genome Sequence of Paenibacillus sp. Strain IHBB 10380 Using PacBio Single-Molecule Real-Time Sequencing Technology. Genome Announc. 2015;3:2.

55. Dhar H, Swarnkar MK, Gulati A, Singh AK, Kasana RC. Draft Genome Sequence of a Cellulase-Producing Psychrotrophic Paenibacillus Strain, IHB B 3415, Isolated from the Cold Environment of the Western Himalayas, India. Genome Announc. 2015;3:1.

56. Liu Y, Wang R, Cao Y, Chen C, Bai F, Xu T, et al. Identification and antagonistic activity of endophytic bacterial strain Paenibacillus sp. 5 L8 isolated from the seeds of maize (Zea mays L., Jingke 968). Ann Microbiol. 2015;66:653-60.

57. Zheng B, Zhang F, Dong H, Chai L, Shu F, Yi S, et al. Draft genome sequence of Paenibacillus sp. strain A2. Stand Genomic Sci. 2016;11:1.
