# Supplementary material for: Current knowledge and perspectives of Paenibacillus: a review
Source: Microb Cell Fact. 2016 Dec 1;15:203. doi: 10.1186/s12934-016-0603-7 (PMC5134293; doi:10.1186/s12934-016-0603-7)
Supplement: Supplementary file 3 — Additional file 3. Secreted compounds and significant enzymes produced by members of the genus Paenibacillus. [file 12934_2016_603_MOESM3_ESM.docx]

**Additional File 3.** Secreted compounds and significant enzymes produced by members of the genus *Paenibacillus.*

| **Species name** | **Secreted compounds and useful enzymes** | **References** |
| --- | --- | --- |
| P. aestuarii | Β-galactosidase, esterase, esterase lipase, leucine arylamidase, cystine arylamidase and naphthol-AS-BI-phosphohydrolase | [1] |
| P. agarexedens | Aminopeptidase | [2] |
| P. agaridevorans | Dextran degrading compound | [2] |
| P. alba  P. algeriensis | Β-galactosidase, α-glucosidase  alkaline phosphatase, esterase (C4), trypsin, α-glucosidase, esterase-lipase | [3]  [4] |
| P. alginolyticus | Xanthan lyase | [5] |
| P. alvei | AN5-1 antimicrobial peptide, antibiotic peptides Paenibacillus P and N, antimicrobial lipopeptides | [6-8] |
| P. amylolyticus | Polymyxin E | [9] |
| P. anaericanus | NO_2_ | [10] |
| P. azoreducens | Azo-dye degrading compound | [11] |
| P. barcinonensis | novel xylanase, esterase, cellulase, and pectinase, GH16 β-glucanase | [12-14] |
| P. barengoltzii | Exochitinase with beta-N-acetylglucosaminidase activity, novel chitinase, pullalanase | [15-17] |
| P. beijingensis | Β-galactosidase, urease | [18] |
| P. brasilensis | Antifungal compound | [19] |
| P. brassicae | alkaline phosphatase, esterase (C4), esterase lipase (C8), leucine arylamidase, cystine arylamidase, acid galactosidase, α-glucosidase, β-glucosidasephosphatase, naphthol-AS-BI-phosphohydrolase | [20] |
| P. campinasensis | Cyclodextrin, alkali-stable pectate lyase | [21, 22] |
| P. castaneae | Β-galactosidase | [23] |
| P. catalpae | esterase (C4), naphthol-AS-BI-phosphohydrolase, α-galactosidase and β-galactosidase; weakly positive for esterase lipase (C8) | [24] |
| P. cellulositrophicus | DNase, urease | [25] |
| P. cellulosilyticus | Cellulases, xylanases, amylases and β-galactosidase | [26] |
| P. chinjuensis | DNase, exopolysaccharides | [27] |
| P. chitinolyticus | Chitinase, cellulase, β-amylase | [28-30] |
| P. chondroitinus | Alginate-degrading compound | [5] |
| P. chungangensis | esterase (C4), leucine arylamidase, naphthol-AS-BI-phosphohydrolase, β-galactosidase and a-fucosidase | [31] |
| P. contaminans | DNase, C4 esterase, leucine arylamidase, β-galactosidase and N-acetyl-β-glucosaminidase | [32] |
| P. cucumis | Acetoin, β-galactosidase | [33] |
| P. curdlanotyticus | Novel xylanase, CMCase, exo-β-xylosidase, endo-xylanase | [5, 34, 35] |
| P. daejeonensis | Antifungal/Antibacterial enzyme SD22, β-galactosidase | [36, 37] |
| P. dendritiformis | IAA, subtilisin protease, lethal lysis protein, biosurfactant | [38-40] |
| P. dongdonensis | Β-galactosidase | [41] |
| P. donghaensis | Xylanase | [42] |
| P. doosanensis | Β-galactosidase | [43] |
| P. durus | Antimicrobial substances, polyhydroxyalkanoate | [44] |
| P. ehimensis | permitin/polypeptin peptide antibiotic production, bacillomycin L/fengycin/plipastatin/agrastatin lipopeptide production, IAA, Butanol, beta-1,3-glucanase, cellulase, chitinase | [45-48] |
| P. elgii | Protocatechuic acid [PCA], exopolysaccharide bioflocculant, broad spectrum antimicrobial compounds, 2,3-dihydroxybenzoate | [49-52] |
| P. endophyticus | Amylase, β-galactosidase | [53] |
| P. favisporus | Xylanases, cellulases, gelatinase, urease, amylase, β-galactosidase | [54, 55] |
| P. ferrarius | NH_3_, H_2_S, alkaline phosphatase, esterase (C4), esterase lipase (C8), leucine arylamidase, valine hydrolase, β-galactosidase, α-glucosidase, b-fucosidase, acid phosphatase, naphthol-AS-BI-phospho-hydrolase | [56] |
| P. filicis | N-acetyl-β-glucosaminidase, esterase lipase, β-glucosidase, leucine arylamidase | [57] |
| P. fonticola | Urease, C4 esterase, naphthol-AS-BI-phosphohydrolase, α-galactosidase and β-galactosidase | [58] |
| P. frigoriresistens | alkaline phosphatase, esterase lipase, leucine arylamidase, valine arylamdase, cystine arylamidase, acidic phosphatase, naphthol–AS–BI–phosphohydrolase, α-galactosidase, β-galactosidase and β-glucosidase | [59] |
| P. ginsengarvi | Β-galactosidase | [60] |
| P. ginsengihumi | esterase (C4), esterase (C8), β-galactosidase, β-glucosidase, naphthol-AS-BI-phosphohydrolase | [61] |
| P. ginsengiterrae | esterase (C4), esterase lipase (C8), leucine arylamidase, acid phosphatase, naphthol-AS-BI-phosphohydrolase, α-galactosidase, β-galactosidase, β-glucosidase | [62] |
| P. glucanolyticus | ethanol, succinic, propanoic, lactic, and malonic acids from black liquor degradation | [63] |
| P. glycanilyticus | two novel α-1,3-glucanases AGL-FH1 and AGL-FH2 | [64] |
| P. gorillae | β-glucuronidase, alkaline phosphatase, α-glucosidase, α-galactosidase, N-acetyl-β-glucosaminidase | [65] |
| P. graminis | Cyclodextrin | [66] |
| P. guangzhouensis | Ferric citrate, β-galactosidase, IAA | [67] |
| P. harenae | esterase (C4), leucine arylamidase, acid phosphatase, naphthol-AS-BI-phosphohydrolase, α-galactosidase, β-galactosidase, a-fucosidase | [68] |
| P. hodogayensis | Exopolysaccharide degrading compound | [69] |
| P. hongkongensis | Β-galactosidase | [70] |
| P. hordei | β-glucosidase, β-galactosidase, acetoin | [71] |
| P. humi | acid phosphatase, α-chymotrypsin, esterase (C4), β-galactosidase, β-glucosidase, leucine arylamidase, naphtol-AS-BI-phosphohydrolase | [72] |
| P. humicus | Mutanase, β-galactosidase | [73, 74] |
| P. hunanensis | Exopolysaccharides, IAA | [75] |
| P. illinoisensis | Cyclodextrin glucanotransferase, chitinases | [76, 77] |
| P. jamilae  P. kobensis | Exopolysaccharides, nitrogenase  Polymyxin M | [78-81] |
| P. koleovorans | Exopolysaccharide degrading compound | [82] |
| P. konsidensis | β-glucosidase, β-galactosidase and N-acetylglucosamine | [83] |
| P. koreensis | Iturin-like antifungal compound, arginine dihydrolase, ornithine decarboxylase, chitin and chitosan degrading compounds | [84] |
| P. kribbensis | Antifungal compound | [85] |
| P. larvae | Paenilarvins, paenilimicins, sevadicin, chitin degrading protein PICBP49 | [86-88] |
| P. lautus | Ectoine hydroxylases, pullulanase | [89, 90] |
| P. lemnae | Β-glucosidase, gelatinase | [91] |
| P. lentimorbus | Endoglucanase | [92] |
| P. lupini | Amylases, caseinase, β-galactosidase | [93] |
| P. macerans | Cyclodextrin glycosyltransferase, 2-O-D-glucopyranosyl-L-ascorbic acid | [94] |
| P. macquariensis | Xylanase, β-glucosidase, β-galactosidase | [95, 96] |
| P. marinisediminis | esterase lipase (C8), lipase (C14), leucine arylamidase, valine arylamidase, α-chymotrypsin, acid phosphatase, naphthol-AS-BI-phosphohydrolase, β-galactosidase, and β-glucosidase | [97] |
| P. marinum | IAA, H2S, alkaline phosphatase, C4 esterase, C8 lipase, C14 lipase, leucine arylamidase, acid phosphatase, naphthol-AS-BI-phosphohydrolase, α-glucosidase, β-glucosidase, a-chymotrypsin, α-galactosidase, β-galactosidase, β-glucuronisidase, β-glucosaminidase | [98] |
| P. mendelii | Lecithinase, β-galactosidase | [99] |
| P. montaniterrae | Xylanase, urease | [100, 101] |
| P. mucilaginosus | Exopolysaccharide bioflocculants | [102] |
| P. nanensis | Xylanase, urease | [103] |
| P. naphthalenovorans | Urease, acetoin, PAH-degrading compound | [104, 105] |
| P. oceanisediminis | alkaline phosphatase, esterase (C4), esterase lipase (C8), acid phosphatase, naphthol-AS-BI-lactosidase, β-glucosidase, leucine arylamidase, β-glucurphosphohydrolase, α-galactosidase, β-galactosidase, β-glucosidase, leucine arylamidase, β-glucuronidase, α-glucosidase | [106] |
| P. pabuli | β-cyclodextrin glycosyltransferase, chitinolytic enzymes | [107, 108] |
| P. panacisoli | Xylan-degrading compound, gelatin-degrading compound | [109] |
| P. pasadenensis | Β-galactosidase | [110] |
| P. pectinilyticus | Gelatin-degrading compound, pectin-degrading compond, β-galactosidase, lysine decarboxylase, ornithine decarboxylase | [111] |
| P. peoriae | Broad spectrum antibacterial and antifungal compounds, chitinases and protease | [112] |
| P. phoenicis | Anti-bacterial compound, anti-fungal compond, chitinases, proteases | [113] |
| P. phyllosphaerae | Xylanase, cellulase, amylase and β-galactosidase | [114] |
| P. pini | Β-galactosidases, β-glucosidases, amylases, mannosidases, chitinases | [115] |
| P. pinihumi | alkaline phosphatase, esterase lipase, leucine arylamidase, α-galactosidase, β-galactosidase, ortho-nitrophenyl-beta-D-galactopyranosidase, acetoin | [116] |
| P. polymyxa | EPS, HCN, NH_3_, flavin reductase, cold-adapted pullulanase, endoarabinase, exo-inulase dibenzothiophene-degrading compound, curdlan cum, β-glycan, endoglucanase, cellulase, alpha-amylase, protease, IAA and other auxins, butanol, (R, R)-2,3-butanediol, acetoin, fusaricidins A/B, novel fusaricidins, lantibiotics, protocatechouic acid, polyketide, colistin A/B, di-n-butyl pthalate, bacteriocins, LI-F homologs, lipopeptide tridecaptin. | [117-142] |
| P. popilliae | Insecticidal proteins | [143] |
| P. profundus | Isocoumarin and novel peptide antibiotics, H2S, alkaline phosphatase, esterase (C4), esterase lipase (C8), acid phosphatase, naphtol-As-BI-phosphohydrolase, β-galactosidase | [144] |
| P. prosopidis | Β-galactosidase, acetoin. Cellulose, starch, arbutin, aesculin and xylan degrading compounds | [145] |
| P. provencensis | Β-galactosidase | [146] |
| P. pueri | Gelatin and starch-degrading compounds, acetoin, β-galactosidase, lysine decarboxylase, ornithine decarboxylase | [147] |
| P. puldeungensis | leucine arylamidase, valine arylamidase, cystine arylamidase, trypsin, a-chymotrypsin, α-galactosidase, β-glucuronidase, α-glucosidase, β-glucosidase | [148] |
| P. purispatii | H_2_S, β-galactosidase | [149] |
| P. relictisesami | Gelatin-degrading compound, sesaminol | [150] |
| P. residui | Urease, acid and alkaline phosphatase, esterase C4, esterase lipase C8, leucine and valine arylamidase, trypsin, a-chymotrypsin, naphthol-AS-BI-phosphohydrolase, β-galactosidase, α-glucosidase, β-glucosidase, N-acetyl-β-glucosaminidase and a-fucosidase | [151] |
| P. rhizosphaerae | Β-galactosidase | [152] |
| P. rigui | a-galactosidase, β-galactosidase, α-glucosidase and leucine arylamidase | [153] |
| P. riograndensis | IAA | [154] |
| P. sacheonensis | Β-galactosidase, xylanolytic compound | [155] |
| P. sediminis | Xylanolytic compound | [156] |
| P. selenii | esterase (C4), esterase lipase (C8), leucine arylamidase, naphthol-AS-BI-phosphohydrolase, β-galactosidase, β-glucosidase, arginine dihydrolase | [157] |
| P. selenitireducens | alkaline phosphatase, esterase lipase (C8), α-galactosidase, b-fucosidase, leucine arylamidase, acid phosphatase, naphthol-AS-BI-phosphohydrolase, β-galactosidase, α-glucosidase and β-glucosidase | [158] |
| P. senegalensis | alkaline phosphatase, esterase lipase, acid phosphatase and naphtol-AS-BI-phosphohydrolase | [159] |
| P. septentrionalis | urease | [100] |
| P. sepulcri | lecithinase, β-galactosidase | [160] |
| P. shenyangensis | Β-galactosidase, gelatinase, caseinase | [161] |
| P. shirakamiensis | alkaline phosphatase, C4 esterase, acid phosphatase, C8 esterase lipase, leucine arylamidase, valine arylamidase, naphthol-AS-BI-phosphohydrolase, β-galactosidase, α-glucosidase, β-glucosidase | [162] |
| P. siamensis | Urease | [100] |
| P. soli | IAA, xylanolytic compound | [163] |
| P. sonchi | Casein-degrading compound | [164] |
| P. sputi | H_2_S, lipase, β-galactosidase | [165] |
| P. stellifer | Nitrogenase, cyclodextrins | [78, 166] |
| P. susongensis | Casein-degrading compound, alkaline phosphatase, esterase (C4), esterase lipase (C8), acid phosphatase, N-acetyl-β-glucuronidase, β-galactosidase, β-glucuronidase, naphthol-AS-BI-phosphohydrolase | [167] |
| P. swuensis | Casein-degrading compound, acid phosphatase, alkaline phosphatase, α-chymotrypsin, esterase (C4), esterase (C8), α-galactosidase, β-galactosidase, naphtol-AS-BI-phosphohydrolase, valine arylamidase | [168] |
| P. taichungensis | IAA, acetoin, alkaline phosphatase, esterase, esterase lipase, leucine arylamidase, valine arylamidase, a-chymotrypsin, acid phosphatase, naphthol-AS-BI-phosphohydrolase, α-galactosidase, β-galactosidase, α-glucosidase, β-glucosidase, N-acetyl-β-glucosaminidase | [169, 170] |
| P. taihuensis | CM-cellulose, aesculin, and starch degrading compounds, H_2_S, alkaline phosphatase, esterase lipase (C8), leucine arylamidase, valine arylamidase, cystine arylamidase, trypsin, a-chymotrypsin, acid phosphatase, naphthol-AS-BI-phosphohydrolase, α-galactosidase, β-galactosidase, α-glucosidase, β-glucosidase | [171] |
| P. taiwanensis | Casein and starch-degrading compounds, β-galactosidase | [172] |
| P. taohuashanense | Β-galactosidase | [173] |
| P. tarimensis | Carboxymethyl cellulase, β-galactosidase, arginine decarboxylase, phenylalanine deaminase | [174, 175] |
| P. telluris | Alkaline phosphatase, esterase, esterase lipase, acid phosphatase, naphtol-AS-BI-phosphohydrolase, α-galactosidase, β-galactosidase, α-glucosidase | [176] |
| P. terrae | Aesculin, casein, gelatin and starch-degrading compounds, carboxymethyl cellulase, cellulase | [177, 178] |
| P. terrigena | Β-galactosidase | [179] |
| P. tezpurensis | Detergent-stable alkaline proteases | [180] |
| P. thailandensis | Xylanase, DNase | [103] |
| P. thermoaerophilus | Chitinase | [181] |
| P. thermophilus | Β-galactosidase | [182] |
| P. thiaminolyticus | Paenibacterin, β-D-galactosidase, β-D-fucosidase | [183, 184] |
| P. tianmuensis | Battacin, β-galactosidase | [185] |
| P. tundrae | Xylan-degrading compound | [186] |
| P. turicensis  P. tylopili | Β-galactosidase  Chitinase | [187]  [188] |
| P. typhae | alkaline phosphatase, esterase (C4), esterase lipase (C8), leucine arylamidase, valine arylamidase, acid phosphatase, naphthol-AS-BI-phosphohydrolase, β-glucosidase | [189] |
| P. tyraminigenes | Antimicrobial substance | [190] |
| P. uliginis | Β-galactosidase | [149] |
| P. urinalis | Β-galactosidase, β-glucosidase | [146] |
| P. validus | Dibenzofuran-degrading compound | [191] |
| P. vulneris | Urease | [192] |
| P. wooponensis | Β-glucosidase, α-galactosidase, β-galactosidase, DNase, caseinase, acid phosphatase, leucine arylamidase, esterase (C4), esterase lipase (C8) | [193] |
| P. woosongensis | Xylanase, keratinase, mannolytic compound | [194-196] |
| P. xylanexedens | Xylan-degrading compound | [186] |
| P. xylaniclasticus | xylanolytic compound, celluloytic compound | [197] |
| P. xylanisolvens | Xylan, aesculin, casein, and urea-degrading compounds | [198] |
| P. xylanilyticus | xylanase, α-L-arabinofuranosidase, β-xylosidase, carboxymethyl cellulase, avicelase, β-glucosidase, β-galactosidase, cellobiohydrolase | [199] |
| P. yonginensis  P. sp. MAEPY1  P. sp. MAEPY2  P. sp. IHB B 3084  P. sp. LLZ1  P. sp. CGMCC5316  P. sp. A3  P. xp. Xy-2  P. sp. BD3526 | IAA, acetoin, α-galactosidase, a-chymotrypsin, β-glucosidase, β-galactosidase, β-galactopyranosidase, leucine arylamidase, alanine arylamidase, tyrosine arylamidase, L-aspartate arylamidase, L-pyrrolidonyl arylamidase, N-acetyl-D-glucosaminidase, esterase, esterase lipase  Cellulase  Cellulase  Cold-active/detergent-stable endoglucanase  Cold-active cellulose  Cyclodextrin glucanotransferase  Penisin (bacteriocin-like peptide)  Paenibacillin A  Metalloproteinase | [200]  [201]  [201]  [202]  [203]  [204]  [205]  [206]  [207] |

1. Bae JY, Kim KY, Kim JH, Lee K, Cho JC, Cha CJ. Paenibacillus aestuarii sp. nov., isolated from an estuarine wetland. Int J Syst Evol Microbiol. 2010;60:644-7.

2. Uetanabaro AP, Wahrenburg C, Hunger W, Pukall R, Spröer C, Stackebrandt E, et al. Paenibacillus agarexedens sp. nov., nom. rev., and Paenibacillus agaridevorans sp. nov. Int J Syst Evol Microbiol. 2003;53:1051-7.

3. Kim HS, Srinivasan S, Lee SS. Paenibacillus alba nov., Isolated from Peat Soil. Curr Microbiol. 2015;70:865-70.

4. Bendjama E, Loucif L, Diene SM, Michelle C, Gacemi-Kirane D, Rolain J-M. Non-contiguous finished genome sequence and description of Paucisalibacillus algeriensis sp nov. Stand Genomic Sci. 2014;9:1352-65.

5. Nakamura LK. Bacillus alginolyticus sp. nov. and Bacillus chondroitinus sp. nov., Two Alginate-Degrading Species. Int J Syst Bacteriol. 1987;37:284-6.

6. Yi T, Huang Y, Chen Y. Production of an antimicrobial peptide an5-1 in Escherichia coli and its dual mechanisms against bacteria. Chem Biol Drug Des. 2015;85:598-607.

7. Anandaraj B, Vellaichamy A, Kachman M, Selvamanikandan A, Pegu S, Murugan V. Co-production of two new peptide antibiotics by a bacterial isolate Paenibacillus alvei NP75. Biochem Biophys Res Commun. 2009;379:179-85.

8. Knolhoff AM, Zheng J, McFarland MA, Luo Y, Callahan JH, Brown EW, et al. Identification and Structural Characterization of Naturally-Occurring Broad-Spectrum Cyclic Antibiotics Isolated from Paenibacillus. J Am Soc Mass Spectrom. 2015;26:1768-79.

9. DeCrescenzo Henriksen E, Phillips DR, Peterson JBD. Polymyxin E production by P. amylolyticus. Lett Appl Microbiol. 2007;45:491-6.

10. Horn MA, Ihssen J, Matthies C, Schramm A, Acker G, Drake HL. Dechloromonas denitrificans sp. nov., Flavobacterium denitrificans sp. nov., Paenibacillus anaericanus sp. nov. and Paenibacillus terrae strain MH72, N2O-producing bacteria isolated from the gut of the earthworm Aporrectodea caliginosa. Int J Syst Evol Microbiol. 2005;55:1255-65.

11. Meehan C, Bjourson AJ, McMullan G. Paenibacillus azoreducens sp. nov., a synthetic azo dye decolorizing bacterium from industrial wastewater. Int J Syst Evol Microbiol. 2001;51:1681-5.

12. Valenzuela SV, Diaz P, Pastor FIJ. Xyn11E from Paenibacillus barcinonensis BP-23: A LppX-chaperone-dependent xylanase with potential for upgrading paper pulps. Appl Microbiol Biotechnol. 2014;98:5949-57.

13. Cerda LA, Valenzuela SV, Diaz P, Pastor FIJ. New GH16 β-glucanase from Paenibacillus barcinonensis BP-23 releases a complex pattern of mixed-linkage oligomers from barley glucan. Biotechnol Appl Biochem. 2016;63:51-6.

14. Sainz-Polo MA, González B, Pastor FIJ, Sanz-Aparicio J. Crystallization and preliminary X-ray diffraction analysis of the N-terminal domain of Paenibacillus barcinonensis xylanase 10C containing the CBM22-1-CBM22-2 tandem. Acta Crystallogr Sect F Struct Biol Commun. 2015;71:136-40.

15. Fu X, Yan Q, Yang S, Yang X, Guo Y, Jiang Z. An acidic, thermostable exochitinase with β-N-acetylglucosaminidase activity from Paenibacillus barengoltzii converting chitin to N-acetyl glucosamine. Biotechnol Biofuels. 2014;7:174.

16. Liu J, Liu Y, Yan F, Jiang Z, Yang S, Yan Q. Gene cloning, functional expression and characterisation of a novel type i pullulanase from Paenibacillus barengoltzii and its application in resistant starch production. Protein Expr Purif. 2016;121:22-30.

17. Yang S, Fu X, Yan Q, Guo Y, Liu Z, Jiang Z. Cloning, expression, purification and application of a novel chitinase from a thermophilic marine bacterium Paenibacillus barengoltzii. Food Chem. 2016;192:1041-8.

18. Wang LY, Li J, Li QX, Chen SF. Paenibacillus beijingensis Sp. Nov., a nitrogen-fixing species isolated from wheat rhizosphere soil. Antonie Van Leeuwenhoek Int J Gen Mol Microbiol. 2013;104:675-83.

19. Elo S, Suominen I, Kämpfer P, Juhanoja J, Salkinoja-Salonen M, Haahtela K. Paenibacillus borealis sp. nov., a nitrogen-fixing species isolated from spruce forest humus in Finland. Int J Syst Evol Microbiol. 2001;51:535-45.

20. Gao M, Yang H, Zhao J, Liu J, Sun YH, Wang YJ, et al. Paenibacillus brassicae sp. nov., isolated from cabbage rhizosphere in Beijing, China. Antonie Van Leeuwenhoek Int J Gen Mol Microbiol. 2013;103:647-53.

21. Yoon J-H, Yim DK, Lee J-S, Shin K-S, Sato HH, Lee ST, et al. Paenibacillus campinasensis sp. nov., a cyclodextrin-producing bacterium isolated in Brazil. Int J Syst Bacteriol. 1998;48:833-7.

22. Liu XX, Zheng XY, Liang SL, Han SY, Lin Y. Expression of the alkaline pectate lyase gene pel from paenibacillus campinasensis BL-11 in pichia pastoris. Mod Food Sci Technol. 2015;31:74-9.

23. Valverde A, Peix A, Rivas R, Velázquez E, Salazar S, Santa-Regina I, et al. Paenibacillus castaneae sp. nov., isolaled from the phyllosphere of Castanea sativa Miller. Int J Syst Evol Microbiol. 2008;58:2560-4.

24. Zhang J, Wang ZT, Yu HM, Ma Y. Paenibacillus catalpae sp. nov., isolated from the rhizosphere soil of Catalpa speciosa. Int J Syst Evol Microbiol. 2013;63:1776-81.

25. Akaracharanya A, Lorliam W, Tanasupawat S, Lee KC, Lee JS. Paenibacillus cellulositrophicus sp. nov., a cellulolytic bacterium from Thai soil. Int J Syst Evol Microbiol. 2009;59:2680-4.

26. Rivas R, Garciá-Fraile P, Mateos PF, Martínez-Molina E, Velázquez E. Paenibacillus cellulosilyticus sp. nov., a cellulolytic and xylanolytic bacterium isolated from the bract phyllosphere of Phoenix dactylifera. Int J Syst Evol Microbiol. 2006;56:2777-81.

27. Yoon JH, Seo WT, Shin YK, Kho YH, Kang KH, Park YH. Paenibacillus chinjuensis sp. nov., a novel exopolysaccharide-producing bacterium. Int J Syst Evol Microbiol. 2002;52:415-21.

28. Jami Al Ahmadi K, Tabatabaei Yazdi M, Fathi Najafi M, Shahverdi AR, Faramarzi MA, Zarrini G, et al. Isolation and characterization of a chitionolytic enzyme producing microorganism, Paenibacillus chitinolyticus JK2 from Iran. Res J Microbiol. 2008;3:395-404.

29. Mihajlovski KR, Carević MB, Dević ML, Šiler-Marinković S, Rajilić-Stojanović MD, Dimitrijević-Branković S. Lignocellulosic waste material as substrate for Avicelase production by a new strain of Paenibacillus chitinolyticus CKS1. International Biodeterioration and Biodegradation. 2015;104:426-34.

30. Mihajlovski KR, Radovanović NR, Miljković MG, Šiler-Marinković S, Rajilić-Stojanović MD, Dimitrijević-Branković SI. β-Amylase production from packaging-industry wastewater using a novel strain Paenibacillus chitinolyticus CKS 1. RSC Adv. 2015;5:90895-903.

31. Park MH, Traiwan J, Jung MY, Nam YS, Jeong JH, Kim W. Paenibacillus chungangensis sp. nov., isolated from a tidal-flat sediment. Int J Syst Evol Microbiol. 2011;61:281-5.

32. Chou JH, Lee JH, Lin MC, Chang PS, Arun AB, Young CC, et al. Paenibacillus contaminans sp. nov., isolated from a contaminated laboratory plate. Int J Syst Evol Microbiol. 2009;59:125-9.

33. Ahn JH, Kim BC, Kim BY, Kim SJ, Song J, Kwon SW, et al. Paenibacillus cucumis sp. nov. isolated from greenhouse soil. J Microbiol. 2014;52:460-4.

34. Sermsathanaswadi J, Pianwanit S, Pason P, Waeonukul R, Tachaapaikoon C, Ratanakhanokchai K, et al. The C-terminal region of xylanase domain in Xyn11A from Paenibacillus curdlanolyticus B-6 plays an important role in structural stability. Appl Microbiol Biotechnol. 2014;98:8223-33.

35. Wongratpanya K, Imjongjairak S, Waeonukul R, Sornyotha S, Phitsuwan P, Pason P, et al. Multifunctional properties of glycoside hydrolase family 43 from Paenibacillus curdlanolyticus strain B-6 including exo-β-xylosidase, endo-xylanase, and α-L-arabinofuranosidase activities. BioResources. 2015;10:2492-505.

36. Lee J-S, Lee KC, Chang Y-H, Hong SG, Oh HW, Pyun Y-R, et al. Paenibacillus daejeonensis sp. nov., a novel alkaliphilic bacterium from soil. Int J Syst Evol Microbiol. 2002;52:2107-11.

37. Zhu K, Zhang XY, Ren Z, Feng DS, Wang YD. Studies on purification and properties of antagonistic protein from bacteria SS02 of Paenibacillus daejeonensis. Shengwu Gongcheng Xuebao/Chin J Biotechnol. 2007;23:681-5.

38. Tcherpakov M, Ben-Jacob E, Gutnick DL. Paenibacillus dendritiformis sp. nov., proposal for a new pattern-forming species and its localization within a phylogenetic cluster. Int J Syst Bacteriol. 1999;49:239-46.

39. Be'er A, Ariel G, Kalisman O, Helman Y, Sirota-Madi A, Zhang HP, et al. Lethal protein produced in response to competition between sibling bacterial colonies. Proc Natl Acad Sci U S A. 2010;107:6258-63.

40. Bezza FA, Nkhalambayausi Chirwa EM. Biosurfactant from Paenibacillus dendritiformis and its application in assisting polycyclic aromatic hydrocarbon (PAH) and motor oil sludge removal from contaminated soil and sand media. Process Saf Environ Prot. 2015;98:354-64.

41. Son JS, Kang HU, Ghim SY. Paenibacillus dongdonensis sp. nov., isolated from rhizospheric soil of Elymus tsukushiensis. Int J Syst Evol Microbiol. 2014;64:2865-70.

42. Choi JH, Im WT, Yoo JS, Lee SM, Moon DS, Kim HJ, et al. Paenibacillus donghaensis sp. nov., a Xylan-degrading and nitrogen-fixing bacterium isolated from east sea sediment. J Microbiol Biotechnol. 2008;18:189-93.

43. Kim JH, Kang H, Kim W. Paenibacillus doosanensis sp. nov., isolated from soil. Int J Syst Evol Microbiol. 2014;64:1271-7.

44. Hungund B, Shyama VS, Patwardhan P, Saleh AM. Production of polyhydroxyalkanoate from paenibacillus durus BV-1 isolated from oil mill soil. J Microb Biochem Technol. 2013;5:13-7.

45. Aktuganov G, Jokela J, Kivelä H, Khalikova E, Melentjev A, Galimzianova N, et al. Isolation and identification of cyclic lipopeptides from Paenibacillus ehimensis, strain IB-X-b. J Chromatogr B Anal Technol Biomed Life Sci. 2014;973:9-16.

46. Aktuganov G, Melentjev A, Galimzianova N, Khalikova E, Korpela T, Susi P. Wide-range antifungal antagonism of Paenibacillus ehimensis IB-X-b and its dependence on chitinase and β-1,3-glucanase production. Can J Microbiol. 2008;54:577-87.

47. Naing KW, Anees M, Nguyen XH, Lee YS, Jeon SW, Kim SJ, et al. Biocontrol of Late Blight Disease (Phytophthora capsici) of Pepper and the Plant Growth Promotion by Paenibacillus ehimensis KWN38. J Phytopathol. 2014;162:367-76.

48. De Araújo NK, De Assis CF, Dos Santos ES, De Macedo GR, De Farias LF, Arimatéia Jr H, et al. Production of enzymes by paenibacillus chitinolyticus and paenibacillus ehimensis to obtain chitooligosaccharides. Appl Biochem Biotechnol. 2013;170:292-300.

49. Nguyen XH, Naing KW, Lee YS, Moon JH, Lee JH, Kim KY. Isolation and characteristics of protocatechuic acid from Paenibacillus elgii HOA73 against Botrytis cinerea on strawberry fruits. J Basic Microbiol. 2015;55:625-34.

50. Li O, Liu A, Lu C, Zheng DQ, Qian CD, Wang PM, et al. Increasing viscosity and yields of bacterial exopolysaccharides by repeatedly exposing strains to ampicillin. Carbohydr Polym. 2014;110:203-8.

51. Kim DS, Bae CY, Jeon JJ, Chun SJ, Oh HW, Hong SG, et al. Paenibacillus elgii sp. nov., with broad antimicrobial activity. Int J Syst Evol Microbiol. 2004;54:2031-5.

52. Lee YS, Nguyen XH, Cho JY, Moon JH, Kim KY. Isolation and antifungal activity of methyl 2,3-dihydroxybenzoate from Paenibacillus elgii HOA73. Microb Pathog. 2016; doi:10.1016/j.micpath.2016.01.007.

53. Carro L, Flores-Félix JD, Cerda-Castillo E, Ramírez-Bahena MH, Igual JM, Tejedor C, et al. Paenibacillus endophyticus sp. nov., isolated from nodules of Cicer arietinum. Int J Syst Evol Microbiol. 2013;63:4433-8.

54. Velázquez E, de Miguel T, Poza M, Rivas R, Rosselló-Mora R, Villa TG. Paenibacillus favisporous sp. nov., a xylanolytic bacterium isolated from cow faeces. Int J Syst Evol Microbiol. 2004;54:59-64.

55. Padilha IQM, Valenzuela SV, Grisi TCL, Diaz P, De Araújo DAM, Javier Pastor FI. A glucuronoxylan-specifi c xylanase from a new Paenibacillus favisporus strain isolated from tropical soil of Brazil. Int Microbiol. 2015;17:175-84.

56. Cao Y, Chen F, Li Y, Wei S, Wang G. Paenibacillus ferrarius sp. nov., isolated from iron mineral soil. Int J Syst Evol Microbiol. 2015;65:165-70.

57. Kim BC, Kim MN, Lee KH, Kwon SB, Bae KS, Shin KS. Paenibacillus filicis sp. nov., isolated from the rhizosphere of the fern. J Microbiol. 2009;47:524-9.

58. Chou JH, Chou YJ, Lin KY, Sheu SY, Sheu DS, Arun AB, et al. Paenibacillus fonticola sp. nov., isolated from a warm spring. Int J Syst Evol Microbiol. 2007;57:1346-50.

59. Ming H, Nie GX, Jiang HC, Yu TT, Zhou EM, Feng HG, et al. Paenibacillus frigoriresistens sp. nov., a novel psychrotroph isolated from a peat bog in heilongjiang, northern china. Antonie Van Leeuwenhoek Int J Gen Mol Microbiol. 2012;102:297-305.

60. Yoon MH, Ten LN, Im WT. Paenibacillus ginsengarvi sp. nov., isolated from soil from ginseng cultivation. Int J Syst Evol Microbiol. 2007;57:1810-4.

61. Kim MK, Kim YA, Park MJ, Yang DC. Paenibacillus ginsengihumi sp. nov., a bacterium isolated from soil in a ginseng field. Int J Syst Evol Microbiol. 2008;58:1164-8.

62. Huq MA, Kim YJ, Hoang VA, Siddiqi MZ, Yang DC. Paenibacillus ginsengiterrae sp. nov., a ginsenoside-hydrolyzing bacteria isolated from soil of ginseng field. Arch Microbiol. 2015;197:389-96.

63. Mathews SL, Pawlak JJ, Grunden AM. Isolation of Paenibacillus glucanolyticus from pulp mill sources with potential to deconstruct pulping waste. Bioresour Technol. 2014;164:100-5.

64. Suyotha W, Yano S, Itoh T, Fujimoto H, Hibi T, Tachiki T, et al. Characterization of α-1,3-glucanase isozyme from Paenibacillus glycanilyticus FH11 in a new subgroup of family 87 α-1,3-glucanase. J Biosci Bioeng. 2014;118:378-85.

65. Keita MB, Padhmananabhan R, Caputo A, Robert C, Delaporte E, Raoult D, et al. Non-contiguous finished genome sequence and description of Paenibacillus gorillae sp nov. Stand Genomic Sci. 2014;9:1031-45.

66. Vollú RE, Da Mota FF, Gomes EA, Seldin L. Cyclodextrin production and genetic characterization of cyclodextrin glucanotranferase of Paenibacillus graminis. Biotechnol Lett. 2008;30:929-35.

67. Li JB, Lu Q, Liu T, Zhou SG, Yang GQ, Zhao Y. Paenibacillus guangzhouensis sp. nov., An Fe(iii)-and humus-reducing bacterium from a forest soil. Int J Syst Evol Microbiol. 2014;64:3891-6.

68. Jeon CO, Lim JM, Lee SS, Chung BS, Park DJ, Xu LH, et al. Paenibacillus harenae sp. nov., isolated from desert sand in China. Int J Syst Evol Microbiol. 2009;59:13-7.

69. Takeda M, Suzuki I, Koizumi JI. Paenibacillus hodogayensis sp. nov., capable of degrading the polysaccharide produced by Sphaerotilus natans. Int J Syst Evol Microbiol. 2005;55:737-41.

70. Teng JLL, Woo PCY, Leung KW, Lau SKP, Wong MKM, Yuen KY. Pseudobacteraemia in a patient with neutropenic fever caused by a novel paenibacillus species: Paenibacillus hongkongensis sp. nov. Molecular Pathology. 2003;56:29-35.

71. Kim JM, Lee SH, Lee SH, Choi EJ, Jeon CO. Paenibacillus hordei sp. nov., isolated from naked barley in Korea. Antonie Van Leeuwenhoek. 2013;103:3-9.

72. Kim HS, Lee SS. Paenibacillus humi sp. nov., isolated from peat-soil. J Gen Appl Microbiol. 2014;60:23-7.

73. Vaz-Moreira I, Faria C, Nobre MF, Schumann P, Nunes OC, Manaia CM. Paenibacillus humicus sp. nov., isolated from poultry litter compost. Int J Syst Evol Microbiol. 2007;57:2267-71.

74. Tsumori H, Shimamura A, Sakurai Y, Yamakami K. Substrate specificity of mutanase of Paenibacillus humicus from fermented food. J Health Sci. 2011;57:78-81.

75. Liu Y, Liu L, Qiu F, Schumann P, Shi Y, Zou Y, et al. Paenibacillus hunanensis sp. nov., isolated from rice seeds. Int J Syst Evol Microbiol. 2010;60:1266-70.

76. Doukyu N, Kuwahara H, Aono R. Isolation of Paenibacillus illinoisensis that produces cyclodextrin glucanotransferase resistant to organic solvents. Bioscience, Biotechnology and Biochemistry. 2003;67:334-40.

77. Jung WJ, An KN, Jin YL, Park RD, Lim KT, Kim KY, et al. Biological control of damping-off caused by Rhizoctonia solani using chitinase-producing Paenibacillus illinoisensis KJA-424. Soil Biol Biochem. 2003;35:1261-4.

78. Jin HJ, Tu R, Xu F, Chen SF. Identification of nitrogen-fixing Paenibacillus from different plant rhizospheres and a novel nifH gene detected in the P. stellifer. Microbiology. 2011;80:117-24.

79. Morillo JA, Aguilera M, Ramos-Cormenzana A, Monteoliva-Sánchez M. Production of a metal-binding exopolysaccharide by Paenibacillus jamilae using two-phase olive-mill waste as fermentation substrate. Curr Microbiol. 2006;53:189-93.

80. Morillo JA, Guerra Del Águila V, Aguilera M, Ramos-Cormenzana A, Monteoliva-Sánchez M. Production and characterization of the exopolysaccharide produced by Paenibacillus jamilae grown on olive mill-waste waters. World J Microbiol Biotechnol. 2007;23:1705-10.

81. Martin NI, Hu H, Moake MM, Churey JJ, Whittal R, Worobo RW, et al. Isolation, Structural Characterization, and Properties of Mattacin (Polymyxin M), a Cyclic Peptide Antibiotic Produced byPaenibacillus kobensis M. J Biol Chem. 2003;278:13124-32.

82. Takeda M, Kamagata Y, Shinmaru S, Nishiyama T, Koizumi JI. Paenibacillus koleovorans sp. nov., able to grow on the sheath of Sphaerotilus natans. Int J Syst Evol Microbiol. 2002;52:1597-601.

83. Ko KS, Kim YS, Lee MY, Shin SY, Jung DS, Peck KR, et al. Paenibacillus konsidensis sp. nov., isolated from a patient. Int J Syst Evol Microbiol. 2008;58:2164-8.

84. Chung Y, Kim C, Hwang I, Chun J. Paenibacillus koreensis sp. nov., a new species that produces an iturin-like antifungal compound. Int J Syst Evol Microbiol. 2000;50:1495-500.

85. Xu SJ, Hong SJ, Choi W, Kim BS. Antifungal activity of Paenibacillus kribbensis strain T-9 isolated from soils against several plant pathogenic fungi. Plant Pathol J. 2014;30:102-8.

86. Garcia-Gonzalez E, Müller S, Ensle P, Süssmuth RD, Genersch E. Elucidation of sevadicin, a novel non-ribosomal peptide secondary metabolite produced by the honey bee pathogenic bacterium Paenibacillus larvae. Environ Microbiol. 2014;16:1297-309.

87. Garcia-Gonzalez E, Poppinga L, Fünfhaus A, Hertlein G, Hedtke K, Jakubowska A, et al. Paenibacillus larvae Chitin-Degrading Protein PlCBP49 Is a Key Virulence Factor in American Foulbrood of Honey Bees. PLoS Pathog. 2014;107.

88. Sood S, Steinmetz H, Beims H, Mohr KI, Stadler M, Djukic M, et al. Paenilarvins: Iturin Family Lipopeptides from the Honey Bee Pathogen Paenibacillus larvae. ChemBioChem. 2014;15:1947-55.

89. Widderich N, Hop̈pner A, Pittelkow M, Heider J, Smits SHJ, Bremer E. Biochemical properties of ectoine hydroxylases from extremophiles and their wider taxonomic distribution among microorganisms. PLoS One. 2014;94.

90. Chen SQ, Cai XH, Xie JL, Wei W, Wei DZ. Structural and biochemical properties of a novel pullulanase of Paenibacillus lautus DSM 3035. Starch. 2016; doi:10.1002/star.201500333.

91. Kittiwongwattana C, Thawai C. Paenibacillus lemnae sp. nov., an endophytic bacterium of duckweed (Lemna aequinoctialis). Int J Syst Evol Microbiol. 2015;65:107-12.

92. Sá ALB, Dias ACF, Quecine MC, Cotta SR, Fasanella CC, Andreote FD, et al. Screening of endoglucanase-producing bacteria in the saline rhizosphere of Rhizophora mangle. Braz J Microbiol. 2014;45:193-7.

93. Carro L, Flores-Félix JD, Ramírez-Bahena MH, García-Fraile P, Martínez-Hidalgo P, Igual JM, et al. Paenibacillus lupini sp. nov., Isolated from nodules of Lupinus albus. Int J Syst Evol Microbiol. 2014;64:3028-33.

94. Xu Q, Han R, Li J, Du G, Liu L, Chen J. Improving maltodextrin specificity by site-saturation engineering of subsite +1 in cyclodextrin glycosyltransferase from Paenibacillus macerans. Shengwu Gongcheng Xuebao/Chin J Biotechnol. 2014;30:98-108.

95. Hoshino T, Nakabayashi T, Hirota K, Matsuno T, Koiwa R, Fujiu S, et al. Paenibacillus macquariensis subsp. defensor subsp. nov., isolated from boreal soil. Int J Syst Evol Microbiol. 2009;59:2074-9.

96. Sharma M, Kumar A. Optimization of xylanase secretion from Paenibacillus macquariensis. Curr Trends Biotechnol Pharm. 2012;6:190-5.

97. Lee HW, Roh SW, Yim KJ, Shin NR, Lee J, Whon TW, et al. Paenibacillus marinisediminis sp. nov., a bacterium isolated from marine sediment. J Microbiol. 2013;51:312-7.

98. Bouraoui H, Rebib H, Aissa MB, Touzel JP, O'Donohue M, Manai M. Paenibacillus marinum sp. nov., a thermophilic xylanolytic bacterium isolated from a marine hot spring in Tunisia. J Basic Microbiol. 2013;53:877-83.

99. Šmerda J, Sedláček I, Páčová Z, Durnová E, Smíšková A, Havel L. Paenibacillus mendelii sp. nov., from surface-sterilized seeds of Pisum sativum L. Int J Syst Evol Microbiol. 2005;55:2351-4.

100. Khianngam S, Tanasupawat S, Lee JS, Lee KC, Akaracharanya A. Paenibacillus siamensis sp. nov., Paenibacillus septentrionalis sp. nov. and Paenibacillus montaniterrae sp. nov., xylanase-producing bacteria from Thai soils. Int J Syst Evol Microbiol. 2009;59:130-4.

101. Arora A, Krishna P, Malik V, Reddy MS. Alkalistable xylanase production by alkalitolerant Paenibacillus montaniterrae RMV1 isolated from red mud. J Basic Microbiol. 2014;54:1023-9.

102. Tang J, Qi S, Li Z, An Q, Xie M, Yang B, et al. Production, purification and application of polysaccharide-based bioflocculant by Paenibacillus mucilaginosus. Carbohydr Polym. 2014;113:463-70.

103. Khianngam S, Akaracharanya A, Tanasupawat S, Lee KC, Lee JS. Paenibacillus thailandensis sp. nov. and Paenibacillus nanensis sp. nov., xylanase-producing bacteria isolated from soil. Int J Syst Evol Microbiol. 2009;59:564-8.

104. Daane LL, Harjono I, Barns SM, Launen LA, Palleroni NJ, Haggblom MM. PAH-degradation by Paenibacillus spp. and description of Paenibacillus naphthalenovorans sp. nov., a naphthalene-degrading bacterium from the rhizosphere of salt marsh plants. Int J Syst Evol Microbiol. 2002;52:131-9.

105. Haggblom MM. Microbe degrades naphthalene. Ind Bioprocessing. 2005;27:2.

106. Lee JN, Shin NR, Jung MJ, Roh SW, Kim MS, Lee JS, et al. Paenibacillus oceanisediminis sp. nov. isolated from marine sediment. Int J Syst Evol Microbiol. 2013;63:428-34.

107. Jemli S, Messaoud EB, Ayadi-Zouari D, Naili B, Khemakhem B, Bejar S. A β-cyclodextrin glycosyltransferase from a newly isolated Paenibacillus pabuli US132 strain: Purification, properties and potential use in bread-making. Biochem Engin J. 2007;34:44-50.

108. Juarez-Jimenez B, Rodelas B, Martinez-Toledo MV, Gonzalez-Lopez J, Crognale S, Gallo AM, et al. Production of chitinolytic enzymes by a strain (BM17) of Paenibacillus pabuli isolated from crab shells samples collected in the east sector of central Tyrrhenian Sea. Int J Biol Macromol. 2008;43:27-31.

109. Ten LN, Baek SH, Im WT, Lee M, Oh HW, Lee ST. Paenibacillus panacisoli sp. nov., a xylanolytic bacterium isolated from soil in a ginseng field in South Korea. Int J Syst Evol Microbiol. 2006;56:2677-81.

110. Osman S, Satomi M, Venkateswaran K. Paenibacillus pasadenensis sp. nov. and Paenibacillus barengoltzii sp. nov., isolated from a spacecraft assembly facility. Int J Syst Evol Microbiol. 2006;56:1509-14.

111. Park DS, Jeong WJ, Lee KH, Oh HW, Kim BC, Bae KS, et al. Paenibacillus pectinilyticus sp. nov., isolated from the gut of Diestrammena apicalis. Int J Syst Evol Microbiol. 2009;59:1342-7.

112. Von Der Weid I, Alviano DS, Santos ALS, Soares RMA, Alviano CS, Seldin L. Antimicrobial activity of Paenibacillus peoriae strain NRRL BD-62 against a broad spectrum of phytopathogenic bacteria and fungi. J Appl Microbiol. 2003;95:1143-51.

113. Benardini JN, Vaishampayan PA, Schwendner P, Swanner E, Fukui Y, Osman S, et al. Paenibacillus phoenicis sp. nov., isolated from the Phoenix Lander assembly facility and a subsurface molybdenum mine. Int J Syst Evol Microbiol. 2011;61:1338-43.

114. Rivas R, Mateos PF, Martínez-Molina E, Velázquez E. Paenibacillus phyllosphaerae sp. nov., a xylanolytic bacterium isolated from the phyllosphere of Phoenix dactylifera. Int J Syst Evol Microbiol. 2005;55:743-6.

115. Yuki M, Oshima K, Suda W, Oshida Y, Kitamura K, Iida T, et al. Draft Genome Sequence of Paenibacillus pini JCM 16418T, Isolated from the Rhizosphere of Pine Tree. Genome Announcements. 2014;2:2.

116. Kim BC, Lee KH, Kim MN, Kim EM, Rhee MS, Kwon OY, et al. Paenibacillus pinihumi sp. nov., a cellulolytic bacterium isolated from the rhizosphere of Pinus densiflora. J Microbiol. 2009;47:530-5.

117. Yegorenkova IV, Tregubova KV, Ignatov VV. Paenibacillus polymyxa rhizobacteria and their synthesized exoglycans in interaction with wheat roots: Colonization and root hair deformation. Curr Microbiol. 2013;66:481-6.

118. Ishii Y, Ohshiro T, Aoi Y, Suzuki M, Izumi Y. Identification of the gene encoding a NAD(P)H-flavin oxidoreductase coupling with dibenzothiophene (DBT)-desulfurizing enzymes from the DBT-nondesulfurizing bacterium Paenibacillus polymyxa A-1. J Biosci Bioeng. 2000;90:220-2.

119. Bai X, Hu H, Chen H, Wei Q, Yang Z, Huang Q. Expression of a β-mannosidase from Paenibacillus polymyxa A-8 in Escherichia coli and characterization of the recombinant enzyme. PLoS One. 2014;9:11.

120. Rafigh SM, Yazdi AV, Vossoughi M, Safekordi AA, Ardjmand M. Optimization of culture medium and modeling of curdlan production from Paenibacillus polymyxa by RSM and ANN. Int J Biol Macromol. 2014;70:463-73.

121. Gastelum-Arellanez A, Paredes-López O, Olalde-Portugal V. Extracellular endoglucanase activity from Paenibacillus polymyxa BEb-40: production, optimization and enzymatic characterization. World J Microbiol Biotechnol. 2014;30:2953-65.

122. Dai JJ, Cheng JS, Liang YQ, Jiang T, Yuan YJ. Regulation of extracellular oxidoreduction potential enhanced (R,R)-2,3-butanediol production by Paenibacillus polymyxa CJX518. Bioresour Technol. 2014;167:433-40.

123. Eastman AW, Weselowski B, Nathoo N, Yuan Z. Complete genome sequence of Paenibacillus polymyxa CR1, a plant growth-promoting bacterium isolated from the corn rhizosphere exhibiting potential for biocontrol, biomass degradation, and biofuel production. Genome Announcements. 2014;2:1.

124. Ko EJ, Byon YY, Jee Y, Shin T, Park SC, Hahn TW, et al. Maturation of bone marrow-derived dendritic cells by a novel β-glucan purified from Paenibacillus polymyxa JB115. J Vet Sci. 2011;12:187-90.

125. Khan Z, Kim SG, Jeon YH, Khan HU, Son SH, Kim YH. A plant growth promoting rhizobacterium, Paenibacillus polymyxa strain GBR-1, suppresses root-knot nematode. Bioresour Technol. 2008;99:3016-23.

126. Gao J, Xu YY, Yang HM, Xu H, Xue F, Li S, et al. Gene cloning, expression, and characterization of an exo-inulinase from Paenibacillus polymyxa ZJ-9. Appl Biochem Biotechnol. 2014;173:1419-30.

127. Häßler T, Schieder D, Pfaller R, Faulstich M, Sieber V. Enhanced fed-batch fermentation of 2,3-butanediol by Paenibacillus polymyxa DSM 365. Bioresour Technol. 2012;124:237-44.

128. Tupinambá G, Da Silva AJR, Alviano CS, Souto-Padron T, Seldin L, Alviano DS. Antimicrobial activity of Paenibacillus polymyxa SCE2 against some mycotoxin-producing fungi. J Appl Microbiol. 2008;105:1044-53.

129. Da Mota FF, Gomes EA, Seldin L. Auxin production and detection of the gene coding for the Auxin Efflux Carrier (AEC) protein in Paenibacillus polymyxa. J Microbiol. 2008;46:257-64.

130. Lee B, Farag MA, Park HB, Kloepper JW, Lee SH, Ryu C-M. Induced Resistance by a Long-Chain Bacterial Volatile: Elicitation of Plant Systemic Defense by a C13 Volatile Produced by Paenibacillus polymyxa. PLoS One. 2012;7:11.

131. Kim HR, Park SY, Kim SB, Jeong H, Choi SK, Park SH. Inactivation of the phosphoglucomutase gene pgm in Paenibacillus polymyxa leads to overproduction of fusaricidin. J Ind Microbiol Biotechnol. 2014;41:1405-14.

132. Xu S, Bai Z, Jin B, Xiao R, Zhuang G. Bioconversion of wastewater from sweet potato starch production to Paenibacillus polymyxa biofertilizer for tea plants. Sci Rep. 2014; doi:10.1038/srep04131.

133. Deng Y, Lu Z, Bi H, Lu F, Zhang C, Bie X. Isolation and characterization of peptide antibiotics LI-F04 and polymyxin B 6 produced by Paenibacillus polymyxa strain JSa-9. Peptides. 2011;32:1917-23.

134. Deng Y, Lu Z, Lu F, Zhang C, Wang Y, Zhao H, et al. Identification of LI-F type antibiotics and di-n-butyl phthalate produced by Paenibacillus polymyxa. J Microbiol Methods. 2011;85:175-82.

135. Li J, Beatty PK, Shah S, Jensen SE. Use of PCR-targeted mutagenesis to disrupt production of fusaricidin-type antifungal antibiotics in Paenibacillus polymyxa. Appl Environ Microbiol. 2007;73:3480-9.

136. Mingchao M, Wang C, Ding Y, Li L, Shen D, Jiang X, et al. Complete genome sequence of Paenibacillus polymyxa SC2, a strain of plant growth-promoting rhizobacterium with broad-spectrum antimicrobial activity. J Bacteriol. 2011;193:311-2.

137. Li S, Zhang R, Wang Y, Zhang N, Shao J, Qiu M, et al. Promoter analysis and transcription regulation of fus gene cluster responsible for fusaricidin synthesis of Paenibacillus polymyxa SQR-21. Appl Microbiol Biotechnol. 2013;97:9479-89.

138. Wei W, Ma J, Chen SQ, Cai XH, Wei DZ. A novel cold-adapted type I pullulanase of Paenibacillus polymyxa Nws-pp2: In vivo functional expression and biochemical characterization of glucans hydrolyzates analysis. BMC Biotechnol. 2015;15:96.

139. Wiratno EN, Suharjono, Wardani AK. Phylogenetic identification of cellulolitic and butanol production bacteria based on 16S rDNA from Ranu Pani lake,east java, Indonesia. Int J ChemTech Res. 2015;8:249-58.

140. Cochrane SA, Lohans CT, Van Belkum MJ, Bels MA, Vederas JC. Studies on tridecaptin B1, a lipopeptide with activity against multidrug resistant Gram-negative bacteria. Org Biomiol Chem. 2015;13:6073-81.

141. Gerst MM, Huang E, Zhang L, Yousef AE. Development of a New Paenibacillin-Producing Strain and Testing its Usability in Improving Food Safety. J Food Sci. 2015;80:M1538-M43.

142. Vater J, Niu B, Dietel K, Borriss R. Characterization of Novel Fusaricidins Produced by Paenibacillus polymyxa-M1 Using MALDI-TOF Mass Spectrometry. J Am Soc Mass Spectrom. 2015;26:1548-58.

143. Federici BA. Insecticidal bacteria: An overwhelming success for invertebrate pathology. J Invertebr Pathol. 2005;89:30-8.

144. Romanenko LA, Tanaka N, Svetashev VI, Kalinovskaya NI. Paenibacillus profundus sp. nov., a deep sediment bacterium that produces isocoumarin and peptide antibiotics. Arch Microbiol. 2013;195:247-54.

145. Valverde A, Fterich A, Mahdhi M, Ramírez-Bahena MH, Caviedes MA, Mars M, et al. Paenibacillus prosopidis sp. nov., isolated from the nodules of Prosopis farcta. Int J Syst Evol Microbiol. 2010;60:2182-6.

146. Roux V, Fenner L, Raoult D. Paenibacillus provencensis sp. nov., isolated from human cerebrospinal fluid, and Paenibacillus urinalis sp. nov., isolated from human urine. Int J Syst Evol Microbiol. 2008;58:682-7.

147. Kim BC, Jeong WJ, Kim DY, Oh HW, Kim H, Park DS, et al. Paenibacillus pueri sp. nov., isolated from Pu'er tea. Int J Syst Evol Microbiol. 2009;59:1002-6.

148. Traiwan J, Park MH, Kim W. Paenibacillus puldeungensis sp. nov., isolated from a grassy sandbank. Int J Syst Evol Microbiol. 2011;61:670-3.

149. Behrendt U, Schumann P, Stieglmeier M, Pukall R, Augustin J, Spröer C, et al. Characterization of heterotrophic nitrifying bacteria with respiratory ammonification and denitrification activity - Description of Paenibacillus uliginis sp. nov., an inhabitant of fen peat soil and Paenibacillus purispatii sp. nov., isolated from a spacecraft assembly clean room. Syst Appl Microbiol. 2010;33:328-36.

150. Shimoyama T, Johari NB, Tsuruya A, Nair A, Nakayama T. Paenibacillus relictisesami sp. nov., isolated from sesame oil cake. Int J Syst Evol Microbiol. 2014;64:1534-9.

151. Vaz-Moreira I, Figueira V, Lopes AR, Pukall R, Spröer C, Schumann P, et al. Paenibacillus residui sp. nov., isolated from urban waste compost. Int J Syst Evol Microbiol. 2010;60:2415-9.

152. Rivas R, Gutiérrez C, Abril A, Mateos PF, Martínez-Molina E, Ventosa A, et al. Paenibacillus rhizosphaerae sp. nov., isolated from the rhizosphere of Cicer arietinum. Int J Syst Evol Microbiol. 2005;55:1305-9.

153. Baik KS, Lim CH, Choe HN, Kim EM, Seong CN. Paenibacillus rigui sp. nov., isolated from a freshwater wetland. Int J Syst Evol Microbiol. 2011;61:529-34.

154. Beneduzi A, Costa PB, Parma M, Melo IS, Bodanese-Zanettini MH, Passaglia LMP. Paenibacillus riograndensis sp. nov., a nitrogen-fixing species isolated from the rhizosphere of Triticum aestivum. Int J Syst Evol Microbiol. 2010;60:128-33.

155. Moon JC, Jung YJ, Jung JH, Jung HS, Cheong YR, Jeon CO, et al. Paenibacillus sacheonensis sp. nov., a xylanolytic and cellulolytic bacterium isolated from tidal flat sediment. Int J Syst Evol Microbiol. 2011;61:2753-7.

156. Wang L, Baek SH, Cui Y, Lee HG, Lee ST. Paenibacillus sediminis sp. nov., a xylanolytic bacterium isolated from a tidal flat. Int J Syst Evol Microbiol. 2012;62:1284-8.

157. Xiang WW, Wang GJ, Wang Yt, Yao R, Zhang FJ, Wang R, et al. Paenibacillus selenii sp. nov., isolated from selenium mineral soil. Int J Syst Evol Microbiol. 2014;64:2662-7.

158. Yao R, Wang R, Wang D, Su J, Zheng SX, Wang GJ. Paenibacillus selenitireducens sp. nov., a selenite-reducing bacterium isolated from a selenium mineral soil. Int J Syst Evol Microbiol. 2014;64:805-11.

159. Mishra AK, Lagier JC, Rivet R, Raoult D, Fournier PE. Non-contiguous finished genome sequence and description of Paenibacillus senegalensis sp. nov. Stand Genomic Sci. 2012;7:70-81.

160. Šmerda J, Sedláček I, Páčová Z, Krejčí E, Havel L. Paenibacillus sepulcri sp. nov., isolated from biodeteriorated mural paintings in the Servilia tomb. Int J Syst Evol Microbiol. 2006;56:2341-4.

161. Jiang B, Zhao X, Liu J, Fu L, Yang C, Hu X. Paenibacillus shenyangensis sp. nov., a bioflocculant-producing species isolated from soil under a peach tree. Int J Syst Evol Microbiol. 2015;65:220-4.

162. Tonouchi A, Tazawa D, Fujita T. Paenibacillus shirakamiensis sp. nov., isolated from the trunk surface of a japanese oak (quercus crispula). Int J Syst Evol Microbiol. 2014;64:1763-9.

163. Park MJ, Kim HB, An DS, Yang HC, Oh ST, Chung HJ, et al. Paenibacillus soli sp. nov., a xylanolytic bacterium isolated from soil. Int J Syst Evol Microbiol. 2007;57:146-50.

164. Hong YY, Ma YC, Zhou YG, Gao F, Liu HC, Chen SF. Paenibacillus sonchi sp. nov., a nitrogen-fixing species isolated from the rhizosphere of Sonchus oleraceus. Int J Syst Evol Microbiol. 2009;59:2656-61.

165. Kim KK, Lee KC, Yu H, Ryoo S, Park Y, Lee JS. Paenibacillus sputi sp. nov., isolated from the sputum of a patient with pulmonary disease. Int J Syst Evol Microbiol. 2010;60:2371-6.

166. Suominen I, Spröer C, Kämpfer P, Rainey FA, Lounatmaa K, Salkinoja-Salonen M. Paenibacillus stellifer sp. nov., a cyclodextrin-producing species isolated from paperboard. Int J Syst Evol Microbiol. 2003;53:1369-74.

167. Guo XQ, Gu JY, Yu YJ, Zhang WB, He LY, Sheng XF. Paenibacillus susongensis sp. nov., a mineral-weathering bacterium. Int J Syst Evol Microbiol. 2014;64:3958-63.

168. Lee JJ, Yang DH, Ko YS, Park JK, Im EY, Kim JY, et al. Paenibacillus swuensis sp. nov., a bacterium isolated from soil. J Microbiol. 2014;52:106-10.

169. Shagol CC, Krishnamoorthy R, Kim K, Sundaram S, Sa T. Arsenic-tolerant plant-growth-promoting bacteria isolated from arsenic-polluted soils in South Korea. Environ Sci Pollut Res. 2014;21:9356-65.

170. Lee FL, Tien CJ, Tai CJ, Wang LT, Liu YC, Chern LL. Paenibacillus taichungensis sp. nov., from soil in Taiwan. Int J Syst Evol Microbiol. 2008;58:2640-5.

171. Wu YF, Wu QL, Liu SJ. Paenibacillus taihuensis sp. nov., isolated from an eutrophic lake. Int J Syst Evol Microbiol. 2013;63:3652-8.

172. Lee FL, Kuo HP, Tai CJ, Yokota A, Lo CC. Paenibacillus taiwanensis sp. nov., isolated from soil in Taiwan. Int J Syst Evol Microbiol. 2007;57:1351-4.

173. Xie JB, Zhang LH, Zhou YG, Liu HC, Chen SF. Paenibacillus taohuashanense sp. nov., a nitrogen-fixing species isolated from rhizosphere soil of the root of Caragana kansuensis Pojark. Antonie Van Leeuwenhoek Int J Gen Mol Microbiol. 2012;102:735-41.

174. Wang MX, Yang M, Zhou GL, Luo XS, Zhang L, Tang Y, et al. Paenibacillus tarimensis sp. nov., isolated from sand in Xinjiang, China. Int J Syst Evol Microbiol. 2008;58:2081-5.

175. Raddadi N, Cherif A, Daffonchio D, Fava F. Halo-alkalitolerant and thermostable cellulases with improved tolerance to ionic liquids and organic solvents from Paenibacillus tarimensis isolated from the Chott El Fejej, Sahara desert, Tunisia. Bioresour Technol. 2013;150:121-8.

176. Lee JC, Kim CJ, Yoon KH. Paenibacillus telluris sp. nov., a novel phosphate-solubilizing bacterium isolated from soil. J Microbiol. 2011;49:617-21.

177. Liang YL, Zhang Z, Wu M, Wu Y, Feng JX. Isolation, screening, and identification of cellulolytic bacteria from natural reserves in the subtropical region of China and optimization of cellulase production by Paenibacillus terrae ME27-1. BioMed Res Int. 2014; doi:10.1155/2014/512497.

178. Yoon JH, Oh HM, Yoon BD, Kang KH, Park YH. Paenibacillus kribbensis sp. nov. and Paenibacillus terrae sp. nov., bioflocculants for efficient harvesting of algal cells. Int J Syst Evol Microbiol. 2003;53:295-301.

179. Xie CH, Yokota A. Paenibacillus terrigena sp. nov., isolated from soil. Int J Syst Evol Microbiol. 2007;57:70-2.

180. Rai SK, Roy JK, Mukherjee AK. Characterisation of a detergent-stable alkaline protease from a novel thermophilic strain Paenibacillus tezpurensis sp. nov. AS-S24-II. Appl Microbiol Biotechnol. 2010;85:1437-50.

181. Ueda J, Kurosawa N. Characterization of an extracellular thermophilic chitinase from Paenibacillus thermoaerophilus strain TC22-2b isolated from compost. World J Microbiol Biotechnol. 2015;31:135-43.

182. Zhou Y, Gao S, Wei DQ, Yang LL, Huang X, He J, et al. Paenibacillus thermophilus sp. nov., a novel bacterium isolated from a sediment of hot spring in Fujian province, China. Antonie Van Leeuwenhoek Int J Gen Mol Microbiol. 2012;102:601-9.

183. Huang E, Yousef AE. Paenibacterin, a novel broad-spectrum lipopeptide antibiotic, neutralises endotoxins and promotes survival in a murine model of Pseudomonas aeruginosa-induced sepsis. Int J Antimicro Agents. 2014;44:74-7.

184. Benešová E, Lipovová P, Dvořáková H, Králová B. β-D-Galactosidase from Paenibacillus thiaminolyticus catalyzing transfucosylation reactions. Glycobiology. 2010;20:442-51.

185. Wu XC, Fang HH, Qian CD, Wen YP, Shen XB, Li O, et al. Paenibacillus tianmuensis sp. nov., isolated from soil. Int J Syst Evol Microbiol. 2011;61:1133-7.

186. Nelson DM, Glawe AJ, Labeda DP, Cann IKO, Mackie RI. Paenibacillus tundrae sp. nov. and Paenibacillus xylanexedens sp. nov., psychrotolerant, xylan-degrading bacteria from Alaskan tundra. Int J Syst Evol Microbiol. 2009;59:1708-14.

187. Bosshard PP, Zbinden R, Altwegg M. Paenibacillus turicensis sp. nov., a novel bacterium harbouring heterogeneities between 16S rRNA genes. Int J Syst Evol Microbiol. 2002;52:2241-9.

188. Kuisiene N, Raugalas J, Spröer C, Kroppenstedt RM, Stuknyte M, Chitavichius D. Paenibacillus tylopili sp.nov., a chitinolytic bacterium isolated from the mycorhizosphere of Tylopilus felleus. Folia Microbiol (Praha). 2008;53:433-7.

189. Kong BH, Liu QF, Liu M, Liu Y, Liu L, Li CL, et al. Paenibacillus typhae sp. nov., isolated from roots of Typha angustifolia L. Int J Syst Evol Microbiol. 2013;63:1037-44.

190. Mah JH, Chang YH, Hwang HJ. Paenibacillus tyraminigenes sp. nov. isolated from Myeolchi-jeotgal, a traditional Korean salted and fermented anchovy. Int J Food Microbiol. 2008;127:209-14.

191. Iida T, Nakamura K, Izumi A, Mukouzaka Y, Kudo T. Isolation and characterization of a gene cluster for dibenzofuran degradation in a new dibenzofuran-utilizing bacterium, Paenibacillus sp. strain YK5. Arch Microbiol. 2006;184:305-15.

192. Glaeser SP, Falsen E, Busse HJ, Kämpfer P. Paenibacillus vulneris sp. nov., isolated from a necrotic wound. Int J Syst Evol Microbiol. 2013;63:777-82.

193. Baik KS, Choe HN, Park SC, Kim EM, Seong CN. Paenibacillus wooponensis sp. nov., isolated from wetland freshwater. Int J Syst Evol Microbiol. 2011;61:2763-8.

194. Yoon KH. Mannanolytic enzyme activity of Paenibacillus woosongensis. Korean J Microbiol. 2010;46:397-400.

195. Yoon KH. Cloning and Characterization of Xylanase Gene from Paenibacillus woosongensis. Korean J Microbiol. 2012;48:141-6.

196. Paul T, Halder SK, Das A, Bera S, Maity C, Mandal A, et al. Exploitation of chicken feather waste as a plant growth promoting agent using keratinase producing novel isolate Paenibacillus woosongensis TKB2. Biocatal Agric Biotechnol. 2013;2:50-7.

197. Tachaapaikoon C, Tanasupawat S, Pason P, Sornyotha S, Waeonukul R, Kyu KL, et al. Paenibacillus xylaniclasticus sp. nov., a xylanolytic-cellulolytic bacterium isolated from sludge in an anaerobic digester. J Microbiol. 2012;50:394-400.

198. Khianngam S, Tanasupawat S, Akaracharanya A, Kim KK, Lee KC, Lee JS. Paenibacillus xylanisolvens sp. nov., a xylan-degrading bacterium from soil. Int J Syst Evol Microbiol. 2011;61:160-4.

199. Rivas R, Mateos PF, Martínez-Molina E, Velázquez E. Paenibacillus xylanilyticus sp. nov., an airborne xylanolytic bacterium. Int J Syst Evol Microbiol. 2005;55:405-8.

200. Sukweenadhi J, Kim YJ, Lee KJ, Koh SC, Hoang VA, Nguyen NL, et al. Paenibacillus yonginensis sp. nov., a potential plant growth promoting bacterium isolated from humus soil of Yongin forest. Antonie Van Leeuwenhoek. 2014;106:935-45.

201. Chien PCT, Yoo HS, Dykes GA, Lee SM. Isolation and characterization of cellulose degrading ability in Paenibacillus isolates from landfill leachate. Malaysia J Microbiol. 2015;11:185-94.

202. Dhar H, Kasana RC, Dutt S, Gulati A. Cloning and expression of low temperature active endoglucanase EG5C from Paenibacillus sp. IHB B 3084. Int J Biol Macromol. 2015;81:259-66.

203. Hu D, Ju X, Li L, Hu C, Yan L, Wu T, et al. Improved in situ saccharification of cellulose pretreated by dimethyl sulfoxide/ionic liquid using cellulase from a newly isolated Paenibacillus sp. LLZ1. Bioresour Technol. 2016;201:8-14.

204. Yu X, Yang J, Li B, Yuan H. High efficiency transformation of stevioside into a single mono-glycosylated product using a cyclodextrin glucanotransferase from Paenibacillus sp. CGMCC 5316. World J Microbiol Biotechnol. 2015;31:1983-91.

205. Baindara P, Chaudhry V, Mittal G, Liao LM, Matos CO, Khatri N, et al. Characterization of the antimicrobial peptide penisin, a class Ia novel lantibiotic from Paenibacillus sp. strain A3. Antimicrob Agents Chemother. 2016;60:580-91.

206. Bian X, Shao M, Pan H, Wang K, Huang S, Wu X, et al. Paenibacillin A, a new 2(1 H)-pyrazinone ring-containing natural product from the endophytic bacterium Paenibacillus sp. Xy-2. Nat Prod Res. 2016;30:125-30.

207. Hang F, Wang Q, Hong Q, Liu P, Wu Z, Liu Z, et al. Purification and characterization of a novel milk-clotting metalloproteinase from Paenibacillus spp. BD3526. Int J Biol Macromol. 2016;85:547-54.
